# X-H Bond Insertion Promoted by Heterogeneous Dirhodium Metal–Organic Cage with Alkynes as Safe Carbene Precursors

**DOI:** 10.3390/molecules28020608

**Published:** 2023-01-06

**Authors:** Lianfen Chen, Chaoyi Zhao, Weixian Mo, Chunsheng Li, Xiaoming Lin

**Affiliations:** 1 Guangdong Provincial Key Laboratory of Environmental Health and Land Resource, School of Environmental and Chemical Engineering, Zhaoqing University, Zhaoqing 526061, China; 2 Laboratory of Theoretical Chemistry of Environment, Ministry of Education, Guangzhou Key Laboratory of Materials for Energy Conversion and Storage, School of Chemistry, South China Normal University, Guangzhou 510006, China

**Keywords:** heterogeneous catalysis, alkyne, carbene, dirhodium, X-H insertion

## Abstract

A facile and efficient methodology for the generation of the C-X (X = Si, B) bond through a carbene insertion process was demonstrated using a dirhodium metal–organic cage, MOC-Rh-**1**, as a heterogeneous catalyst. A series of functionalized alkynes were utilized as safe carbene precursors to furnish Si-H and B-H insertion products in moderate to excellent yields. These reactions featured a high atom-economy, a broad substrate scope, and mild reaction conditions. Moreover, the as-prepared MOC-Rh-**1** catalyst was recovered easily from the reaction system by simple centrifugation and reused for ten runs without a significant loss in activity, which made good use of the valuable precious metal rhodium.

## 1. Introduction

Dinuclear rhodium(II) tetracarboxylates have attracted great interest for many years, which were proven to be exceptionally active and highly versatile catalysts for carbene and nitrene transfer reactions [1,2,3,4,5]. Rhodium(II) complexes, especially the chiral rhodium(II) carboxylic acids, are indispensable catalysts in many processes for the production of pharmaceuticals and fine chemicals, including cyclopropanations, X-H insertions, ylide transformations, and so on [6,7,8]. Recently, dirhodium(II) complexes even found use in photocatalysis for hydrogen evolution and carbon dioxide reduction [9,10,11]. Despite high activity and selectivity, the unavoidable high cost, the difficulty in recovering and recycling, and the metal contamination of pharmaceuticals became the main factors that limited the application of dirhodium catalysts in the chemical industry.

To address these issues, an efficient solution is to immobilize homogeneous dirhodium catalysts into/onto heterogeneous support materials. A series of materials have been applied as the host, such as mesoporous silica, crystalline nanocellulose, coordination polymers, metal–organic frameworks (MOFs), and metal–organic cages (MOCs) [12,13,14,15] Porous materials based on MOCs have received much attention by acting not only as heterogeneous catalysts but also as building blocks of infinite MOFs [16,17,18,19,20,21,22]. Herein, we employed a metal–organic cage, as we previously reported [14], to heterogenize dirhodium catalysts by the self-assembly of Rh_2_(OAc)_4_ with ditopic carboxylic ligands. 

Organosilicon and organoboron compounds are extensively used in the fields of organic synthesis, materials science, and organometallic chemistry. Much effort has been devoted to developing efficient synthetic methodologies to gain structurally diverse organosilicon and organoboron compounds. The construction of Si-C bonds can be realized by alkyne hydrosilylation [23,24], a sila-Heck type process [25], or silyl conjugated addition [26]. As for organoboron compounds, several protocols, including the hydroborations of olefins and C-H bond borylations, offer straightforward access to establish B-C bond [27,28,29]. In addition to the above-mentioned methods, the insertion of carbene species into the X-H (X = Si, B) bond stands for another highly efficient route to construct Si-C and B-C bonds [30,31]. The past few decades have witnessed the tremendous development of X-H insertions. Among various carbene precursors, diazo compounds are one of the most traditional and popular kinds, which featured mild reaction conditions, high reactivity and selectivity. However, the danger of explosion and dimerization tendency limited the wide application of diazo compounds. 

**Figure 1:**
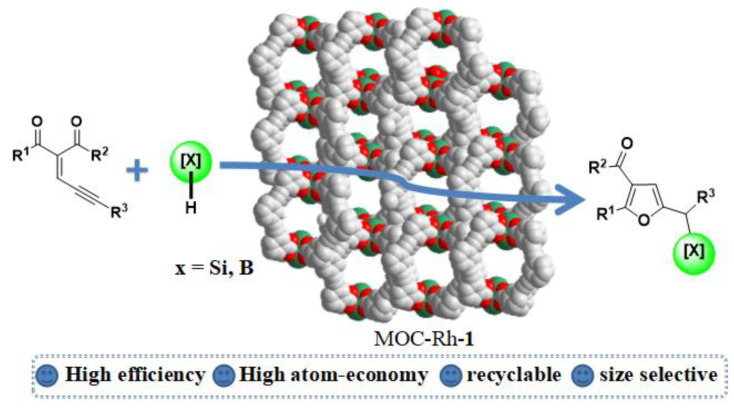
MOC-Rh-**1** promoted X-H insertions (X = Si, B).

Alkynes, as emerging non-diazo carbene precursors, have been gaining much attention recently [32,33]. Increasing numbers of carbene transfer reactions are being reported, starting from functionalized alkynes under the catalysis of transition metals. Dirhodium complexes have shown high reactivity and selectivity in reactions with alkynes as carbene precursors, especially asymmetric processes [34,35,36,37]. However, to the best of our knowledge there is no report on the heterogeneous version. Herein, we demonstrated the insertions of alkyne-derived carbene into Si-H and B-H bonds in the presence of a dirhodium-based metal–organic cage (MOC-Rh-**1**) as a heterogeneous catalyst (Figure 1). The reactions feature a mild reaction condition, high efficiency with up to 97% isolated yield, and a broad substrate scope. The catalyst showed excellent recyclability and could be reused for ten runs. Moreover, size selectivity was observed in the MOC-Rh-**1**-promoted B-H insertion. 

## 2. Results and Discussion

To investigate the catalytic activity of MOC-Rh-**1** in the carbene transfer reaction via the non-diazo approach, conjugated enynones were employed as carbene precursors. In our initial study, enynone **1a** and trisubstituted silane **2a** were chosen as model substrates in the presence of 1 mol% of MOC-Rh-**1**. The reaction was conducted in toluene at room temperature under ambient atmosphere. Delightfully, the desired Si-H insertion product **3a** was obtained in 84% yield after 1 h (Table 1, entry 1). To improve the reaction efficiency, several other solvents were then explored. CH_2_Cl_2_ and CHCl_3_ led to comparable yields with that of toluene (entries 2 and 3). 1,4-Dioxane, acetone, and tetrahydrofuran (THF) were proved to be inferior solvent with reduced yields (entries 4–6). The reaction in THF only gave rise to **3a** in 38% yield, which might be ascribed to the existence of the active C-H bond disturbing the Si-H insertion process. When it came to 1,2-dichloroethane (DCE) as the solvent, the isolated yield of the expected product could be enhanced to 89% (entry 7). Frustratingly, when we tried to lower the catalyst loading to 0.5 mol%, the yield decreased to 82% (entry 8). The homogeneous mode of this reaction was also carried out with Rh_2_(CH_3_COO)_4_, Rh_2_(OPiv)_4,_ and Rh_2_(CF_3_COO)_4_ as catalysts (entries 9–11). The reaction functioned well and was completed within 5 min, with the highest yield being 91% for Rh_2_(OPiv)_4_. The homogeneous Rh_2_(CH_3_COO)_4_ was quite efficient, even when the catalyst loading was lowered to 0.05 mol%, giving 85% yield in 30 min (entry 12). As for MOC-Rh-**1**, only 35% yield was obtained under the same reaction conditions (entry 13). These results indicated that the heterogeneous MOC-Rh-**1** catalyst was not as effective as the homogeneous analogues, which is a common phenomenon in a heterogeneous catalysis system. Control experiments indicated that the reaction did not occur in the absence of catalysts (entry 14).

**Scheme 1:**
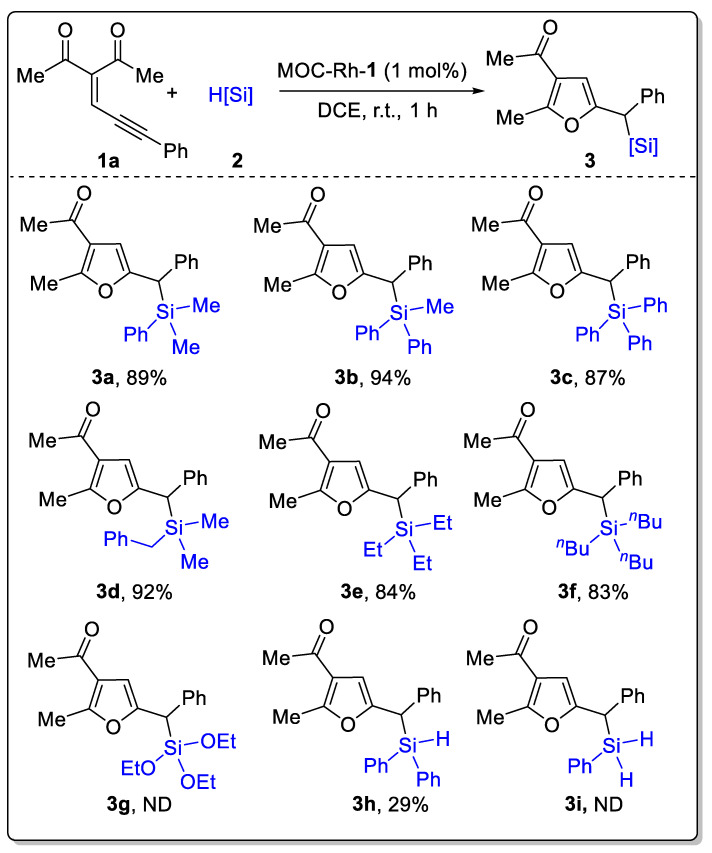
Substrate scope for the Si-H insertion reactions of enynone **1a** with various silanes **2**. The reactions were performed at r.t. for 1 h under air with 0.2 mmol of **1a**, 2.0 equiv. of **2**, and 1 mol% of MOC-Rh-**1**. ND: not detected.

With the optimized reaction conditions in hand, we set out to explore the substrate scope of this Si-H insertion. The heterogeneous MOC-Rh-**1** catalytic system proved to be effective with a variety of trisubstituted silanes. As illustrated in Scheme 1, the combination of standard substrates **1a** and **2a** (PhSiMe_2_H) could give rise to **3a** in 89% yield. It is noteworthy that the bulky silanes Ph_2_SiMeH and Ph_3_SiH also functioned well to generate products **3b** and **3c** in excellent yields, thus demonstrating that the catalytic efficiency was not limited by the pore size of MOC-Rh-**1**. Silane **2d** tethered with benzyl group could successfully give **3d** in 92% yield. When the rhodium carbenoid was trapped by less sterically hindered trialkylsilanes Et_3_SiH and *^n^*Bu_3_SiH, the desired products **3e** and **3f** were obtained in 84% and 83% yields, respectively. However, the silane bearing electron-donating ethoxyl moiety led to a complex system with no desired product detected (**3g**). Except for the above trisubstituted silanes, the disubstituted substrate was examined as well. Similar to the catalytic results in the homogeneous reactions reported by zhu and co-workers [34]_,_ Ph_2_SiH_2_ showed passable activity to give **3h** in 29% yield. As for mono-substituted silane PhSiH_3_, no reaction took place in 24 h, and a messy mixture was observed in further prolonged time.

**Scheme 2:**
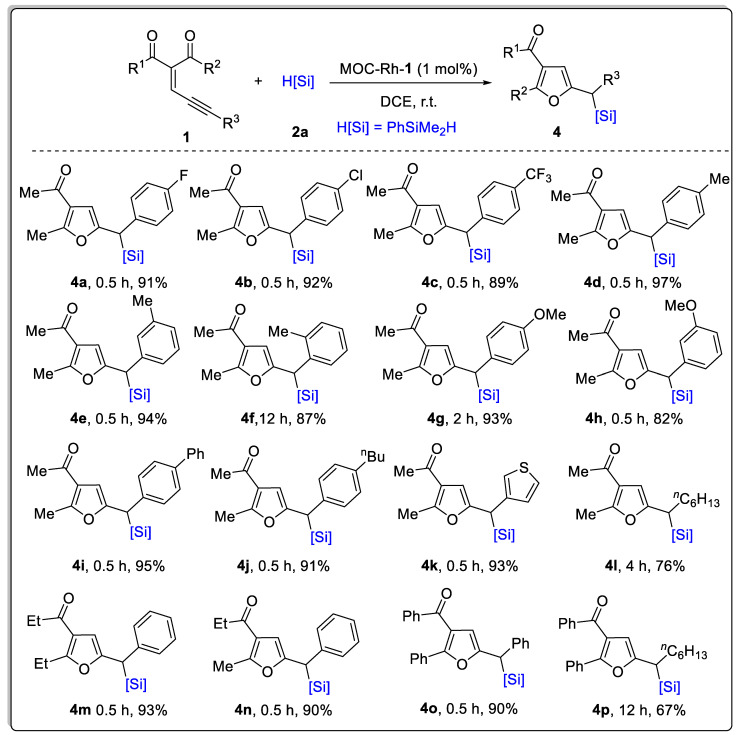
Substrate scope for the Si-H insertion reactions of various enynones **1** with **2a**, H[Si] = PhSiMe_2_H. The reactions were performed at r.t. for 1 h under air with 0.2 mmol of **1**, 2.0 equiv. of **2a** and 1 mol% of MOC-Rh-**1**. Isolated yield.

To further demonstrate the practicality and scalability of the present heterogeneous protocol, we moved on to investigate the reactions of various enynones **1** with the standard silane **2a** (Scheme 2). In the presence of 1 mol% MOC-Rh-**1**, enynones containing the halogen group smoothly participated in the reaction with **2a**, furnishing the organosilicon products in excellent yields (**4a** and **4b**). This process was also compatible with the strong electron-withdrawing -CF_3_ group (**4c**, 89%). The steric effect was studied by comparing the reactivities of enynones with the methyl group substituted on different positions. It was illustrated that the less hindered *para*- and *meta*-substituted substrates could smoothly afford the expected products in high yields in 0.5 h (97% for **4d** and 94% for **4e**). As for the *ortho*-substituted one, the reaction took much longer time to give product **4f** in a decreased yield (87%). When the electron-donating methoxyl group was introduced, the reaction also went well to furnish the products. However, the activity was slighted effected, as illustrated by the longer reaction time for **4g** (2 h) and relatively lower yield for **4h** (82%). Enynone substituted with phenyl or *^n^*Bu group served as an effective substrate as well, producing the Si-H insertion products **4i** and **4j** in 95% and 91% yields, respectively. Except for the phenyl group, the thiofuran ring was also well-tolerated to give **4k** in 93% yield. When it came to the alkyl group, the yield declined to 76% (**4l**). Furthermore, the propionyl- and benzoyl-substituted enynones could be utilized as active substrates, which all successfully transformed into the corresponding products in excellent yields (**4m**–**4o**). However, enynones with alkyl moiety only led to a 67% yield (**4p**). The results of **4l** and **4p** indicated that substrates with the alkyl group on alkyne moiety might be inferior candidates for this reaction.

**Figure 2:**
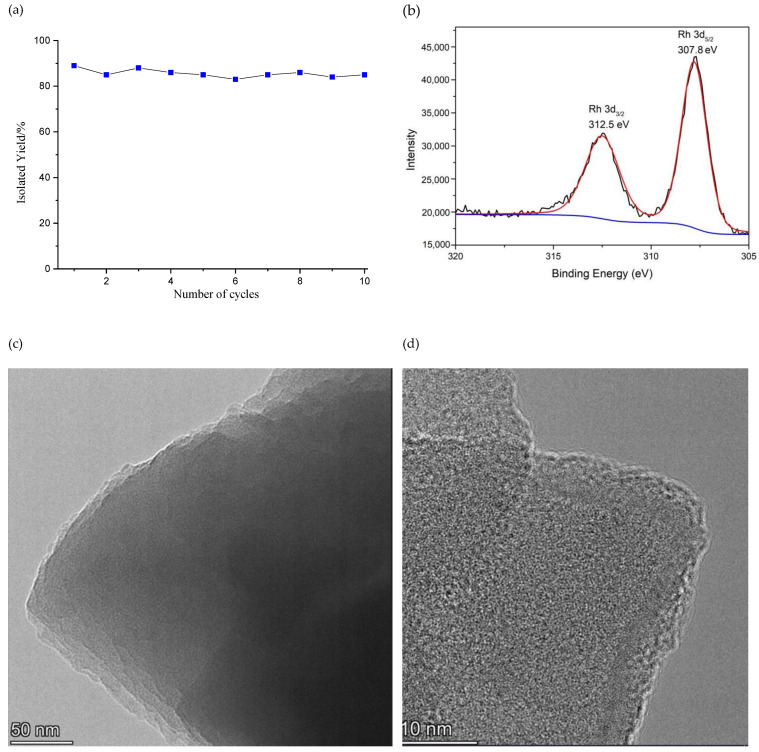
(**a**) Recycling experiment results; (**b**) XPS of MOC-Rh-**1** after catalysis; and (**c**,**d**) TEM images of MOC-Rh-**1** after catalysis.

The recyclability of precious metal is of great importance in the heterogeneous catalysis system. Ten successive Si-H insertion reactions of functionalized alkyne **1a** with silane **2a** were carried out to evaluate the reusability of MOC-Rh-**1**. Due to the heterogeneous nature and high catalytic activity of this dirhodium catalyst, the regeneration operation is quite simple and feasible. After each reaction cycle, MOC-Rh-**1** was separated from the solution by centrifugation and air-dried for further use without activation under vacuum. Remarkably, the MOC-Rh-**1** catalyst could be recycled for at least ten runs without significant loss of activity, showing 83–89% yields (Figure 2a).

To evaluate the heterogeneity of the catalyst, a hot filtration experiment was carried out by removing the MOC-Rh-**1** catalyst after 15 min of the reaction, and 53% conversion was observed. Then, the filtrate was allowed to stand at room temperature for another 45 min, giving 65% conversion with a 12% increase. The inductively coupled plasma optical emission spectrometer (ICP-OES) measurement of the reaction filtrate indicated that 1.0% of the total Rh content in the catalyst was leached into the solution, thus leading to the 12% increased conversion. It is reasonable to conclude that MOC-Rh-**1** was basically heterogeneous in this process. The valence state and morphology of MOC-Rh-**1** after catalysis were also investigated to figure out the form of existence of the catalyst. X-ray photo-electron spectroscopy (XPS) indicated Rh in MOC-Rh-**1** after catalysis remained +2 valence by displaying Rh 3d_3/2_ and 3d_5/2_ peaks at 312.5 and 307.8 eV, respectively (Figure 2b). Transmission electron microscopy (TEM) showed that the catalyst was uniformly arranged without observation of Rh(0) nanopaticles (Figure 2c,d). Therefore, it should be the Rh(II) in MOC-Rh-**1** that mainly acted as a catalytic active site.

**Scheme 3:**
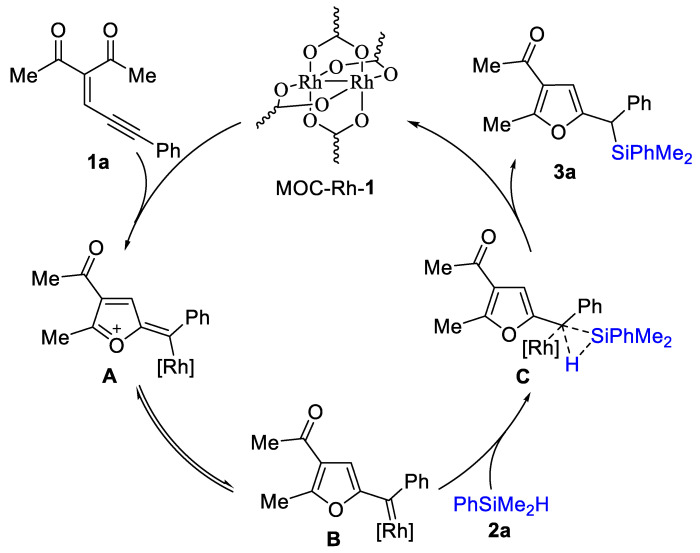
A plausible reaction mechanism.

Based on literature reports and the above investigation results, a possible reaction mechanism for the MOC-Rh-**1**-promoted Si-H insertion was proposed with standard substrates **1a** and **2a** as examples (Scheme 3). Firstly, the alkyne group of **1a** was activated by the Rh(II) sites in MOC-Rh-**1**, which was then attacked by the carbonyl group to undergo a *5-exo-dig* cyclization. Furanium vinyl-metal intermediate **A** was generated through the above process, which was resonant with 2-furanyl metal-carbene **B**. Then, the carbene intermediate **B** was trapped by silane **2a** to give intermediate **C**, followed by protodemetallation to furnish the final Si-H insertion product **3a** with regeneration of the catalyst.

**Scheme 4:**
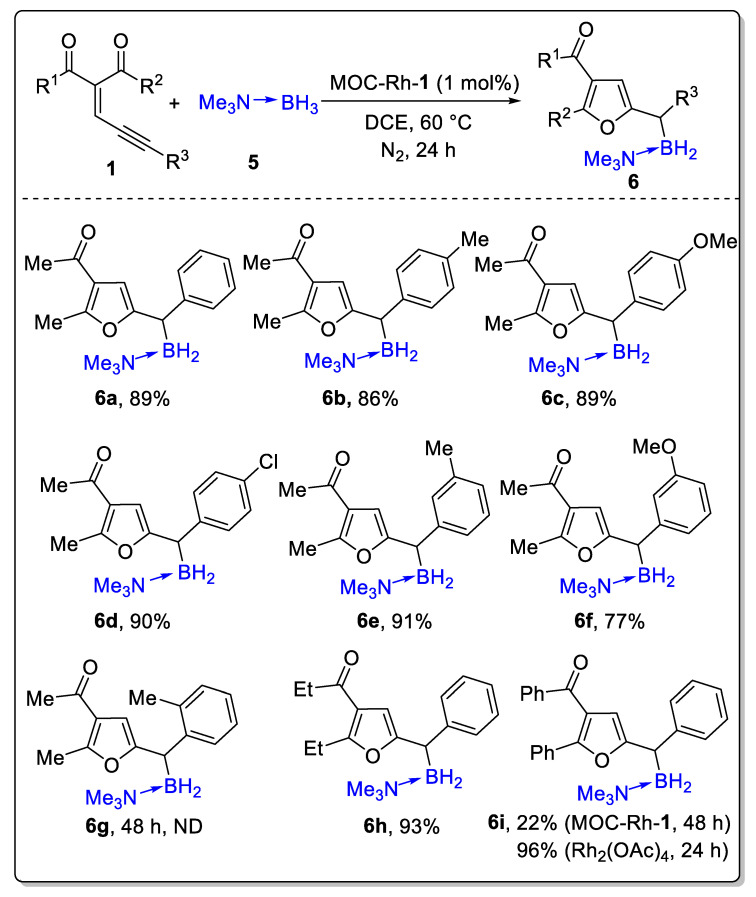
Substrate scope for the B-H insertion reactions of various enynones with trimethylamine borane **5**. The reactions were performed at 60 °C for 24 h under N_2_ atmosphere with 0.2 mmol of **1**, 2.0 equiv. of **5** and 1 mol% of MOC-Rh-**1** unless otherwise noted. Isolated yield.

Except for the Si-H bond, the insertion of carbene species generated from alkyne precursors into the B-H bond was explored as well (Scheme 4). A variety of enynones were subjected to the reaction with trimethylamine borane **5** in the presence of MOC-Rh-**1**. The reactions were conducted in DCE at 60 °C under an N_2_ atmosphere. Furan-2-ylmethylborane product **6a** was smoothly generated in 89% yield. 4-Methyl and 4-methoxyl-substituted enynones were both successfully converted into the desired products in high yields (**6b** and **6c**). Enynone with the electron-withdrawing chloro group on the *para*-position of the aryl ring served as an effective substrate to give **6d** in 90% yield. When the methyl and methoxyl moiety were introduced onto the *meta*-position, the corresponding borane adducts **6e** and **6f** could be smoothly generated. It is noted that no desired product was detected when it came to *ortho*-substituted enynone, which might be due to the steric hindrance effect (**6g**). R^1^ and R^2^ could also be ethyl moiety, furnishing **6h** in 93% yield. When bulky phenyl groups were tethered on the carbonyl moiety, the reaction efficiency dramatically decreased to give **6i** in only 22% yield in 48 h. To shed light on the reason for the low activity, Rh_2_(OAc)_4_ was employed in the B-H insertion process as a contrast to the heterogeneous porous MOC-Rh-**1** catalyst. The homogeneous version displayed high efficiency with 96% yield, illustrating that the low yield was unlikely due to the substrate itself. Therefore, the porous catalyst MOC-Rh-**1** displayed size selectivity in the B-H insertion reaction. Substrates with smaller sizes resulted in a higher yield (**6a** for example), whereas that with a larger size led to a lower yield (**6i**, 22% yield). It might be that the channels in MOC-Rh-**1** limited the transportation of larger substrates, leading to less active sites being accessible and thus a loss of activity.

## 3. Conclusions

We disclosed the transformations of the functionalized alkynes as carbene precursors under the catalysis of a metal–organic cage (MOC-Rh-**1**), which contains dirhodium clusters as both building blocks and active sites. The heterogeneous and porous MOC-Rh-**1** demonstrated high efficiency in promoting the Si-H insertion of alkyne-derived carbene intermediate into silanes. Being compatible with various enynones and silanes, this protocol displayed a broad substrate scope as well as mild reaction conditions and high atom-economy. What is more, MOC-Rh-**1** could be reused for ten runs without a significant loss of activity. The basic heterogeneity of the catalyst was verified by a hot filtration experiment and an ICP test. The XPS and TEM results indicated that the Rh species in MOC-Rh-**1** remained +2 valence after catalysis. Furthermore, MOC-Rh-**1** was effective in terms of carbene insertion into B-H bonds as well, giving borane adducts in up to 93% yield. It is noted that due to the porosity of MOC-Rh-**1**, size selective catalysis was observed in the B-H insertion reactions. MOC-Rh-**1** exhibited potential in the heterogeneous catalysis involving carbene species derived from various precursors.

## 4. Materials and Methods

### 4.1. General

All the reagents were obtained from commercial sources and used directly without further purification unless otherwise noted. The solvent dimethylacetamide used in the synthesis of MOC-Rh-**1** is GC pure (99.8%). The ligand 3,3′-[1,3-phenylenebis(ethyne-2,1-diyl)] dibenzoic acid (H_2_pbeddb) was prepared according to the literature [38]. The enynone substrates were prepared following the reported procedures [39]. The ^1^H NMR and ^13^C NMR spectra were recorded on Bruker AVANCE III 400 MHz or 500 MHz. The ^1^H NMR and ^13^C NMR chemical shifts were determined relative to internal standard TMS at *δ* 0.0. Chemical shifts (*δ*) are reported in ppm, and coupling constants (*J*) are in Hertz (Hz). ICP spectroscopy was conducted on PE Avio ICP-OES spectrometer. TEM images were recorded on Talos F200S G2 spectrometer. XPS was performed on a ULVAC PHI Quantera microprobe. Binding energies (BE) were calibrated by setting the measured BE of C 1s to 284.65 eV.

### 4.2. Preparation of MOC-Rh-**1** Catalyst

MOC-Rh-**1** was prepared according to our previous report with a slight modification [33]**:** H_2_pbeddb (18.3 mg, 0.05 mmol), Rh_2_(OAc)_4_ (8.8 mg, 0.02 mmol), Na_2_CO_3_ (6.0 mg 0.057 mmol), and dimethylacetamide (DMAC, 4 mL) were placed in a glass vial and heated at 100 °C in a preheated oven. After 24 h, green block crystals were obtained. The solids were collected, washed with DMAC and acetone, and activated under vacuum at 100 °C for 12 h before catalysis.

### 4.3. General Procedure for the Catalytic Si-H Insertion Reactions

Enynone (0.2 mmol) and silane (0.4 mmol) were added to the suspension of MOC-Rh-**1** (4.0 mg, 0.002 mmol, and 1 mol%) in 1,2-dichloroethane (2 mL) The whole mixture was stirred under air at room temperature until the full consumption of the enynone substrate (monitored by TLC) took place. The reaction mixture was filtrated through a pad of silica gel and purified by chromatography (petroleum ether/ethyl acetate) to yield the desired Si-H insertion product. The NMR data of the products are listed in the Supplementary Materials.

### 4.4. General Procedure for the Recycling Experiment

A mixture of **1a** (424 mg, 2 mmol), PhSiMe_2_H (4 mmol), and activated MOC-Rh-**1** (40.0 mg, 1 mol%) in 1,2-dichloroethane (20 mL) was stirred under air at room temperature for 1 h. The catalyst was separated by centrifugation, washed with ethyl acetate (5 mL × 3), dried under air, and reused in the consecutive run. The resulting residue was purified by chromatography to determine the isolated yield.

### 4.5. Procedure for Hot Filtration

Enynone (0.2 mmol) and silane (0.4 mmol) were added to the suspension of MOC-Rh-**1** (4.0 mg, 0.002 mmol, and 1 mol%) in 1,2-dichloroethane (2 mL). The whole mixture was stirred under air at room temperature for 15 min. Then, the reaction mixture was filtrated with a filtration membrane (0.45 mm), and the filtrate was allowed to stir for another 45 min.

### 4.6. Procedure for ICP-OES Experiment

Enynone (0.6 mmol) and silane (1.2 mmol) were added to the suspension of MOC-Rh-**1** (12.0 mg, 0.006 mmol, and 1 mol%, containing ca. 2.460 mg of Rh) in 1,2-dichloroethane (6 mL). The whole mixture was stirred under air at room temperature until full consumption of enynone. Then, the reaction mixture was filtrated with a filtration membrane (0.45 mm), and the filtrate was digested by acid before ICP-OES evaluation. The measured Rh content was 0.0242 mg, and the leached Rh content was 1.0%.

### 4.7. General Procedure for the Catalytic B-H Insertion Reactions

Enynone (0.2 mmol) and trimethylamine borane **5** (0.4 mmol) were added to the suspension of MOC-Rh-**1** (4.0 mg, 0.002 mmol, and 1 mol%) in 1,2-dichloroethane (2 mL). The whole mixture was stirred under N_2_ at 60 °C until the full consumption of the enynone substrate (monitored by TLC) was achieved. The reaction mixture was filtrated through a pad of silica gel and purified by chromatography to yield the desired B-H insertion product.The NMR data of the products are listed in the Supplementary Materials.

## Figures and Tables

**Table 1:** Optimization of the reaction conditions ^a^.

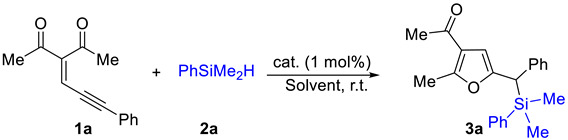			
Entry	Catalyst	Solvent	Yield (%) ^b^
1	MOC-Rh-**1**	Toluene	84
2	MOC-Rh-**1**	CH_2_Cl_2_	81
3	MOC-Rh-**1**	CHCl_3_	85
4	MOC-Rh-**1**	1,4-dioxane	74
5	MOC-Rh-**1**	Acetone	62
6	MOC-Rh-**1**	THF	38
7	MOC-Rh-**1**	DCE	89
8 ^c^	MOC-Rh-**1**	DCE	82
9 ^d^	Rh_2_(CH_3_COO)_4_	DCE	89
10 ^d^	Rh_2_(OPiv)_4_	DCE	91
11 ^d^	Rh_2_(CF_3_COO)_4_	DCE	82
12 ^e^	Rh_2_(CH_3_COO)_4_	DCE	85
13 ^e^	MOC-Rh-**1**	DCE	35
14	none	DCE	NR

^a^ The reaction was performed at r. t. for 1 h under air with 0.2 mmol of **1a**, 2.0 equiv. of **2a,** and 1 mol% of catalyst. [**1a**] = 0.1 M; THF: tetrahydrofuran, and DCE: 1,2-dichloroethane. OPiv: pivalate, NR: no reaction. ^b^ Isolated yield. ^c^ 0.5 mol% of catalyst. ^d^ reaction time: 5 min. ^e^ 0.05 mol% of catalyst, 30 min.

## Data Availability

Not applicable.

## Supplementary Materials

The following supporting information can be downloaded at https://www.mdpi.com/article/10.3390/molecules28020608/s1, the NMR data and spectra of the catalytic products.

## Sample Availability

A sample of MOC-Rh-**1** is unavailable from the authors upon reasonable request.
