# Supplementary material for: X-H Bond Insertion Promoted by Heterogeneous Dirhodium Metal–Organic Cage with Alkynes as Safe Carbene Precursors
Source: Molecules. 2023 Jan 6;28(2):608. doi: 10.3390/molecules28020608 (PMC9865382; doi:10.3390/molecules28020608)
Supplement: Supplementary file 1 [file molecules-28-00608-s001.zip › molecules-2108186-supplementary.pdf]

Supplementary Materials

X-H Bond Insertion Promoted by Heterogeneous Dirhodium Metal–Organic Cage with Alkynes as Safe Carbene Precursors

Lianfen Chen [1,*], Chaoyi Zhao [1], Weixian Mo [1], Chunsheng Li [1] and Xiaoming Lin [2,*]

[1] Guangdong Provincial Key Laboratory of Environmental Health and Land Resource, School of Environmental and Chemical Engineering, Zhaoqing University, Zhaoqing, Guangdong 526061, China

[2] Laboratory of Theoretical Chemistry of Environment, Ministry of Education, Guangzhou Key Laboratory of Materials for Energy Conversion and Storage, School of Chemistry, South China Normal University, Guangzhou 510006, China

* Correspondence: chenlianfen@zqu.edu.cn (C. L.); linxm@scnu.edu.cn (L. X.)

1. NMR data of the products

1-(5-((dimethyl(phenyl)silyl)(phenyl)methyl)-2-methylfuran-3-yl)ethan-1-one (**3a**): yellow oil, 89% yield. ^{1}H NMR (400 MHz, $CDCl_3$) δ 7.33-7.24 (m, 5H), 7.18 (m, 2H), 7.13-7.07 (m, 1H), 7.03-6.99 (m, 2H), 6.16 (s, 1H), 3.70 (s, 1H), 2.51 (s,3H), 2.28 (s, 3H), 0.32 (s, 6H). ^{13}C NMR (*101 MHz*, $CDCl_3$) δ 194.30, 156.75, 154.10, 139.67, 136.50, 134.23, 129.46, 128.32, 128.09, 127.66, 125.53, 122.25, 106.47, 38.27, 29.18, 14.46, -3.60, -3.80.

1-(2-methyl-5-((methyldiphenylsilyl)(phenyl)methyl)furan-3-yl)ethan-1-one (3b): yellow oil, 94% yield. ^{1}H NMR (400 MHz, $CDCl_3$) δ 7.45-7.26 (m, 10H), 7.20- 7.08 (m, 3H), 6.99-6.97 (m, 2H), 6.12 (s, 1H), 4.11 (s, 1H), 2.46 (s, 3H), 2.26 (s, 3H), 0.58 (s, 3H). ^{13}C NMR (*101 MHz*, $CDCl_3$) δ 194.30, 156.77, 153.54, 139.20, 135.19, 135.10, 134.92, 134.67, 129.64, 129.57, 128.55, 128.23, 127.73, 127.71, 125.66, 122.24, 107.18, 36.83, 29.09, 14.33, -4.48.

1-(2-methyl-5-(phenyl(triphenylsilyl)methyl)furan-3-yl)ethan-1-one (3c): yellow oil, 87% yield. ^{1}H NMR (400 MHz, $CDCl_3$) δ 7.44-7.39 (m, 3H), 7.36-7.27 (m, 12H), 7.15-7.12(m, 3H), 6.99-6.96 (m, 2H), 6.04 (s, 1H), 4.45 (s, 1H), 2.40 (s, 3H), 2.25 (s, 3H). ^{13}C NMR (*101 MHz*, $CDCl_3$) δ 194.45, 156.70, 153.16, 138.51, 136.47, 133.03, 129.77, 129.37, 128.19, 127.69, 125.92, 122.19, 107.56, 36.33, 29.10, 14.23.

1-(5-((benzyldimethylsilyl)(phenyl)methyl)-2-methylfuran-3-yl)ethan-1-one (3d): light yellow oil, 92% yield. ^{1}H NMR (500 MHz, $CDCl_3$) δ 7.45-7.42 (m, 2H), 7.35-7.21 (m, 5H), 7.24-7.21 (m, 1H), 7.08-7.06(m, 2H), 6.40 (s, 1H), 3.71 (s, 1H), 2.72 (s, 3H), 2.52 (s, 3H), 2.33- 2.24 (m, 2H), 0.20 (s, 3H), 0.12 (s, 3H).^{13}C NMR (126 MHz, $CDCl_3$) δ *1*94.24, 156.82, 154.32, 139.90, 139.17, 128.52, 128.28, 128.26, 127.96, 125.58, 124.30, 122.33, 106.30, 77.34, 77.08, 76.83, 37.11, 29.16, 24.45, 14.48, -3.92, -4.07.

1-(2-methyl-5-(phenyl(triethylsilyl)methyl)furan-3-yl)ethan-1-one (3e): light yellow oil, 84% yield. ^{1}H NMR (500 MHz, $CDCl_3$) δ 7.27-7.24 (m, 2H), 7.20-7.18 (m, 2H), 7.16-7.13 (m, 1H), 6.22 (s, 1H), 3.61 (s, 1H), 2.57 (s, 3H), 2.35 (s, 3H), 0.87 (t, *J* =7.9 Hz, 9H), 0.74-0.37 (m, 6H). ^{13}C NMR (126 MHz, $CDCl_3$) δ 194.30, 156.57, 154.81, 140.34, 128.40, 127.91, 125.33, 122.27, 105.67, 35.30, 29.12, 14.44, 7.24, 2.97.

1-(2-methyl-5-(phenyl(tributylsilyl)methyl)furan-3-yl)ethan-1-one (3f): light yellow oil, 83% yield. ^{1}H NMR (500 MHz, $CDCl_3$) δ 7.27-7.24 (m, 2H), 7.20-7.11 (m, 3H), 6.21 (s, 1H), 3.60 (s, 1H), 2.56 (s, 3H), 2.35 (s, 3H), 1.8-1.24 (m, 6H), 1.19-1.15 (m, 6H), 0.83 (t, *J* =7.3 Hz, 9H), 0.59-

0.52 (m, 6H). ^{13}C NMR (126 MHz, $CDCl_3$) δ 194.35, 156.50, 154.89, 140.37, 128.36, 127.95, 125.31, 122.22, 105.63, 35.89, 29.13, 26.72, 26.69, 25.80, 14.43, 13.67, 11.56.

1-(5-((diphenylsilyl)(phenyl)methyl)-2-methylfuran-3-yl)ethan-1-one (3h): light yellow oil, 29% yield. ^{1}H NMR (500 MHz, $CDCl_3$) δ 7.44-7.41(m, 2H), 7.37-7.26 (m, 6H), 7.25-7,22 (m, 2H), 7.20-7.10 (m, 3H), 7.07-7.04 (m, 2H), 6.23 (s, 1H), 5.13-5.12 (m, 1H), 4.14-4.13 (m, 1H), 2.46 (s, 3H), 2.27 (s, 3H). ^{13}C NMR (126 MHz, $CDCl_3$) δ 194.43, 157.23, 153.00, 138.97, 135.68, 135.63, 132.05, 131.96, 130.13, 130.04, 128.53, 128.47, 128.00, 127.92, 125.96, 122.29, 107.45, 35.04, 29.16, 14.41.

1-(5-((dimethyl(phenyl)silyl)(4-fluorophenyl)methyl)-2-methylfuran-3-yl)ethan-1-one (4a): light yellow oil, 91% yield. ^{1}H NMR (500 MHz, $CDCl_3$) δ 7.379-7.37 (m, 1H), 7.32-7.31 (m, 4H), 7.01-6.94 (m, 2H), 6.93-6.88 (m, 2H), 6.17 (s, 1H), 3.70 (s, 1H), 2.55 (s, 3H), 2.34 (s, 3H), 0.35 (s, 3H), 0.34 (s, 3H). ^{13}C NMR (126 MHz, $CDCl_3$) δ194.21, 161.02 (d, J = 243.6 Hz), 156.82, 153.86, 136.17, 135.31 (d, J = 3.2 Hz), 134.15, 129.50, 129.34, 129.28, 127.67, 122.23, 115.02 (d, J = 21.2 Hz), 106.42, 37.42, 29.10, 14.39, -3.86, -3.89. ^{19}F NMR (471 MHz, CDCl3) δ -117.92.

1-(5-((4-chlorophenyl)(dimethyl(phenyl)silyl)methyl)-2-methylfuran-3-yl)ethan-1-one (4b): yellow oil, 92% yield. ^{1}H NMR (500 MHz, $CDCl_3$) δ 7.40-7.35(m, 1H), 7.32-7.31 (m, 4H), 7.18-7.17 (m, 2H), 6.94-6.93 (m, 2H), 6.18 (s, 1H), 3.70(s, 1H), 2.55 (s, 3H), 2.33 (s, 3H), 0.35 (s, 3H), 0.34 (s, 3H). ^{13}C NMR (126 MHz, $CDCl_3$) δ 194.18, 156.88, 153.49, 138.26, 136.00, 134.15, 131.20, 129.57, 129.27, 128.34, 127.70, 122.24, 106.61, 37.72, 29.12, 14.40, -3.87, -3.89.

1-(5-((dimethyl(phenyl)silyl)(4-(trifluoromethyl)phenyl)methyl)-2-methylfuran-3-yl)ethan-1-one (4c). yellow oil, 89% yield. ^{1}H NMR (500 MHz, $CDCl_3$) δ 7.48 (d, J =8.1 Hz, 1H), 7.44-7.37 (m, 0H), 7.34 (d, J =4.5 Hz, 2H), 7.13 (d, J =8.0 Hz, 1H), 6.24 (s, 1H), 3.83 (s, 1H), 2.58 (s, 1H), 2.37 (s, 1H), 0.38 (d, J =2.7 Hz, 4H).^{13}C NMR (126 MHz, $CDCl_3$) δ 194.13, 157.02, 152.96, 144.11, 135.70, 134.12, 129.69, 128.11, 127.75, 127.64 (q, J(C-F)=31.1 Hz)125.13 (q, J(C-F)=3.7 Hz), 122.27, 106.92, 38.50, 29.11, 14.39, -3.93, -4.00.

1-(5-((dimethyl(phenyl)silyl)(*p*-tolyl)methyl)-2-methylfuran-3-yl)ethan-1-one (4d): yellow oil, 97% yield. ^{1}H NMR (500 MHz, $CDCl_3$) δ 7.41-7.30 (m, 5H), 7.06-7.04 (m, 2H), 6.98-6.90 (m, 2H), 6.17 (s, 1H), 3.70 (s, 1H), 2.55 (s, 3H), 2.33 (s, 3H), 2.32 (s, 3H), 0.36 (s, 6H). ^{13}C NMR (126 MHz, $CDCl_3$) δ 194.35, 156.65, 154.34, 136.68, 136.50, 134.93, 134.19, 129.35, 128.99, 127.98, 127.59, 122.19, 106.24, 37.71, 29.11, 20.97, 14.40, -3.55, -3.87.

1-(5-((dimethyl(phenyl)silyl)(*m*-tolyl)methyl)-2-methylfuran-3-yl)ethan-1-one (4e): yellow oil, 94% yield. ^{1}H NMR (500 MHz, $CDCl_3$) δ 7.38-7.24 (m, 5H), 7.10-7.07 (m, 1H), 6.94-6.93(m, 1H), 6.83-6.82 (m, 1H), 6.76 (s, 1H), 6.15 (s, 1H), 3.65 (s, 1H), 2.52 (s, 3H), 2.30(s, 3H), 2.24 (s, 3H), 0.32-0.31 (m, 6H). ^{13}C NMR (126 MHz, $CDCl_3$) δ 194.35, 156.69, 154.16, 139.47, 137.73, 136.60, 134.22, 129.38, 128.94, 128.13, 127.55, 126.24, 125.08, 122.23, 106.39, 38.09, 29.13, 21.48, 14.43, -3.65, -3.87.

1-(5-((dimethyl(phenyl)silyl)(*o*-tolyl)methyl)-2-methylfuran-3-yl)ethan-1-one (4f): yellow oil, 87% yield. ^{1}H NMR (500 MHz, $CDCl_3$) δ 8.46-8.29 (m, 5H), 8.26-8.22 (m, 1H), 8.20- 8.06 (m, 3H), 7.18 (s, 1H), 5.00 (s, 1H), 3.57 (s, 3H), 3.35 (s, 3H), 3.14 (s, 3H), 1.43 (s, 3H), 1.40 (s, 3H). ^{13}C NMR (126 MHz, $CDCl_3$) δ 194.39, 156.59, 154.58, 137.96, 136.76, 135.06, 134.12, 130.49, 129.41, 128.57, 127.61, 125.92, 125.53, 122.19, 106.07, 33.05, 29.14, 20.23, 14.43, -3.31, -3.82.

1-(5-((dimethyl(phenyl)silyl)(4-methoxyphenyl)methyl)-2-methylfuran-3-yl)ethan-1-one (4g): yellow oil, 93% yield. ^{1}H NMR (500 MHz, $CDCl_3$) δ 7.40-7.28 (m, 5H), 6.97 (d, J =8.7 Hz, 2H), 6.79 (d, J =8.7 Hz, 2H), 6.15 (s,1H), 3.78 (s, 3H), 3.67 (s,1H), 2.55 (s, 3H), 2.33 (s, 3H), 0.35-0.34 (m, 6H).^{13}C NMR (126 MHz, $CDCl_3$) δ 194.39, 157.57, 156.68, 154.48, 136.64, 134.20, 131.59, 129.36, 129.02, 127.61, 122.18, 113.75, 106.12, 55.22, 37.11, 29.12, 14.42, -3.59, -3.85.

1-(5-((dimethyl(phenyl)silyl)(3-methoxyphenyl)methyl)-2-methylfuran-3-yl)ethan-1-one (4h): yellow oil, 82% yield. ^{1}H NMR (500 MHz, $CDCl_3$) δ 7.38-7.29 (m, 5H), 7.14 (t, J =7.9 Hz,1H), 6.71-6.69(m, 1H), 6.65-6.63 (m, 1H), 6.55 -6.54 (m, 1H), 6.19 (s, 1H), 3.70 (s, 1H), 3.67 (s, 3H), 2.55 (s, 3H), 2.33 (s, 3H), 0.36-0.35 (m, 6H). ^{13}C NMR (126 MHz, $CDCl_3$) δ 194.34, 159.44, 156.76, 153.89, 141.20, 136.53, 134.20, 129.41, 129.16, 127.61, 122.20, 120.51, 113.79, 110.97, 106.48, 55.01, 38.29, 29.11, 14.41, -3.71, -3.82.

1-(5-([1,1'-biphenyl]-4-yl(dimethyl(phenyl)silyl)methyl)-2-methylfuran-3-yl)ethan-1-one (4i): yellow oil, 95% yield. ^{1}H NMR (500 MHz, $CDCl_3$) δ 7.62 (d, J =7.6 Hz, 2H), 7.50 (d, J =7.2 Hz,

2H), 7.45 (t, J =7.6 Hz, 2H), 7.43 – 7.30 (m, 3H), 7.15-7.13 (m, 3H), 6.25 (s, 1H), 3.81 (s, 3H), 2.60 (s, 3H), 2.37 (s, 3H), 0.42 (s, 6H). ^{13}C NMR (126 MHz, $CDCl_3$) δ 194.31, 156.81, 153.96, 140.88, 138.84, 138.32, 136.45, 134.24, 129.49, 128.79, 128.46, 127.67, 127.11, 126.94, 126.91, 122.28, 106.52, 37.98, 29.16, 14.46, -3.61, -3.78.

1-(5-((4-butylphenyl)(dimethyl(phenyl)silyl)methyl)-2-methylfuran-3-yl)ethan-1-one (4j): yellow oil, 91% yield. ^{1}H NMR (400 MHz, $CDCl_3$) δ 7.39-7.28 (m, 5H), 7.06 (d, J =7.9 Hz, 2H), 6.97 (d, J =7.9 Hz, 2H), 6.19 (s, 1H), 3.71 (s, 1H), 2.61-2.56 (m, 5H), 2.34 (s, 3H), 1.65-1.57 (m, 2H), 1.42-1.33(m, 2H), 0.96 (t, J =7.3 Hz, 1H), 0.37 (s, 3H), 0.36 (s, 3H). ^{13}C NMR (*101 MHz*, $CDCl_3$) δ 194.35, 156.64, 154.34, 139.98, 136.69, 136.64, 134.20, 129.34, 128.31, 127.92, 127.57, 122.20, 106.22, 37.76, 35.19, 33.65, 29.14, 22.39, 14.42, 14.03, -3.58, -3.85.

1-(5-((dimethyl(phenyl)silyl)(thiophen-3-yl)methyl)-2-methylfuran-3-yl)ethan-1-one (4k): yellow oil, 93% yield. ^{1}H NMR (500 MHz, $CDCl_3$) δ 7.38-7.32 (m, 1H), 7.31-7.26 (m, 4H), 7.17 (dd, J =5.0, 3.0 Hz, 1H), 6.79-6.73 (m, 2H), 6.10 (s, 1H), 3.86 (s, 1H), 2.53 (s, 3H), 2.31 (s, 3H), 0.33 (s, 3H), 0.30 (s, 3H). ^{13}C NMR (126 MHz, $CDCl_3$) δ 194.33, 156.57, 153.78, 139.02, 136.43, 134.09, 129.44, 128.21, 127.63, 124.98, 122.19, 119.69, 105.65, 33.70, 29.13, 14.40, -3.91.

1-(5-(1-(dimethyl(phenyl)silyl)heptyl)-2-methylfuran-3-yl)ethan-1-one (4l): yellow oil, 76% yield. ^{1}H NMR (500 MHz, $CDCl_3$) δ 7.46-7.41 (m, 2H), 7.38-7.30 (m, 3H), 5.98 (s, 1H), 2.50 (s, 3H), 2.35 (s, 3H), 2.25 (dd, J =11.4, 4.0 Hz, 1H), 1.73-1.48 (m, 2H), 1.40-1.07 (m, 8H), 0.84 (t, J =7.0 Hz, 3H), 0.32 (s, 3H), 0.25 (s, 3H). ^{13}C NMR (126 MHz, $CDCl_3$) δ 194.48, 156.06, 155.69, 137.26, 133.89, 129.17, 127.66, 122.17, 104.31, 31.63, 29.28, 29.13, 28.93, 28.27, 22.61, 14.34, 14.05, -3.96, -4.93.

1-(5-((dimethyl(phenyl)silyl)(phenyl)methyl)-2-ethylfuran-3-yl)propan-1-one (4m): yellow oil, 93% yield. ^{1}H NMR (500 MHz, $CDCl_3$) δ 7.35-7.25 (m, 5H), 7.21-7.18 (m, 2H), 7.13-7.10 (m, 1H), 7.04-7.02 (m, 2H), 6.13 (s, 1H), 3.71 (s, 1H), 2.99 (q, J =7.6 Hz, 2H), 2.62 (q, J =7.3 Hz, 2H), 1.22 (t, J =7.6 Hz, 3H), 1.10 (t, J =7.3 Hz, 3H), 0.32 (s, 6H). ^{13}C NMR (126 MHz, $CDCl_3$) δ 197.33, 161.54, 154.04, 139.80, 136.57, 134.13, 129.36, 128.27, 128.05, 127.60, 125.45, 120.60, 105.80, 38.43, 34.39, 21.66, 12.15, 7.93, -3.60, -3.85.

1-(5-((dimethyl(phenyl)silyl)(phenyl)methyl)-2-methylfuran-3-yl)propan-1-one (4n): yellow oil, 90% yield. ^{1}H NMR (400 MHz, $CDCl_3$) δ 7.44-7.35 (m, 5H), 7.27-7.25 (m, 2H), 7.20-7.17(m, 1H), 7.09 (d, J =7.9 Hz, 2H), 6.19 (s, 1H), 3.78 (s, 1H), 3.07-3.01 (q, J =7.6 Hz, 2H), 2.36 (s, 3H), 1.30-1.27 (t, J =7.5 Hz, 3H), 0.39 (s, 6H). ^{13}C NMR (*101 MHz*, $CDCl_3$) δ 194.36, 161.66, 154.13, 139.76, 136.54, 134.14, 129.39, 128.29, 128.05, 127.63, 125.48, 121.21, 106.28, 38.40, 29.21, 21.67, 12.14, -3.58, -3.83.

(5-((dimethyl(phenyl)silyl)(phenyl)methyl)-2-phenylfuran-3-yl)(phenyl)methanone (4o): yellow oil, 90% yield. ^{1}H NMR (400 MHz, $CDCl_3$) δ 7.83-7.80 (m, 2H), 7.75-7.71(m, 2H), 7.53-7.51 (m, 1H), 7.46-7.28 (m, 12H), 7.24-7.10 (m, 3H), 6.29 (s, 1H), 3.94 (s, 1H), 0.580.27 (m, 6H). ^{13}C NMR (*101 MHz*, $CDCl_3$) δ 192.02, 155.22, 154.23, 139.33, 138.28, 136.51, 134.23, 132.77, 130.09, 129.80, 129.50, 128.64, 128.45, 128.38, 128.33, 128.19, 127.80, 127.17, 125.67, 121.73, 109.97, 38.78, -3.40, -3.93

(5-(1-(dimethyl(phenyl)silyl)heptyl)-2-phenylfuran-3-yl)(phenyl)methanone (4p): yellow oil,76% yield. ^{1}H NMR (500 MHz, $CDCl_3$) δ 7.79-7.72 (m, 2H), 7.66- 7.57 (m, 2H), 7.51-7.44 (m, 3H), 7.39-7.32 (m, 5H), 7.28-7.21 (m, 3H), 6.04 (s, 1H), 2.40 (dd, J =11.7, 3.6 Hz, 1H), 1.77-1.73 (m, 1H), 1.68-1.62(m, 1H), 1.41-1.37(m, 1H), 1.28-1.18 (m, 8H), 0.83 (t, J =7.0 Hz, 3H), 0.41 (s, 3H), 0.36 (s, 3H). ^{13}C NMR (126 MHz, $CDCl_3$) δ 192.19, 156.89, 153.65, 138.46, 137.20, 133.93, 132.60, 130.25, 129.76, 129.25, 128.35, 128.22, 127.82, 127.00, 121.66, 31.67, 29.81, 29.45, 28.98, 28.28, 22.65, 14.08, -4.02, -4.50.

1-(5-(trimethylamine-boranyl(phenyl)methyl)-2-methylfuran-3-yl)ethan-1-one (6a): light yellow oil,89%. ^{1}H NMR (500 MHz, $CDCl_3$) δ 7.30-7.25 (m, 2H), 7.22 (t, J =7.5 Hz, 2H), 7.06 (t, J =7.3 Hz, 1H), 6.29 (s, 1H), 3.35 (t, J =5.3 Hz, 1H), 2.49 (s, 3H), 2.46 (s, 9H), 2.35 (s, 3H).^{13}C NMR (126 MHz, $CDCl_3$) δ 194.87, 160.82, 156.04, 147.34, 128.20 (d, J =1.4 Hz), 124.49, 122.10, 104.54, 52.56, 37.89, 29.14, 14.50.

1-(5-(trimethylamine-boranyl(p-tolyl)methyl)-2-methylfuran-3-yl)ethan-1-one (6b): colorless oil, **86%.** ^{1}H NMR (500 MHz, $CDCl_3$) δ 7.18 (d, J =8.1 Hz, 2H), 7.04 (d, J =7.8 Hz, 2H), 6.27 (s, 1H), 3.32 (t, J =5.3 Hz, 1H), 2.49 (s, 3H), 2.47 (s, 9H), 2.35 (s, 3H), 2.27 (s, 3H). ^{13}C NMR (126 MHz, $CDCl_3$) δ 194.98, 161.07, 156.05, 144.14, 133.80, 128.96, 128.07, 122.06, 104.35, 52.58, 37.10, 29.17, 20.97, 14.54.

1-(5-(trimethylamine-boranyl(4-methoxyphenyl)methyl)-2-methylfuran-3-yl)ethan-1-one (6c): colorless oil, 89%. ^{1}H NMR (500 MHz, $CDCl_3$) δ 7.20 (d, J =8.6 Hz, 2H), 6.78 (d, J =8.7 Hz, 2H), 6.22 (s, 1H), 3.74 (s, 3H), 2.49 (s, 3H), 2.45 (s, 9H), 2.33 (s, 3H). ^{13}C NMR (126 MHz, $CDCl_3$) δ 194.94, 161.23, 156.85, 156.00, 139.27, 129.05, 122.06, 113.71, 104.15, 77.40, 77.15, 76.89, 55.22, 52.57, 36.70, 29.17, 14.53.

1-(5-(trimethylamine-boranyl(4-chlorophenyl)methyl)-2-methylfuran-3-yl)ethan-1-one (6d): light yellow oil, 90%. ^{1}H NMR (500 MHz, $CDCl_3$) δ 7.23-7.16 (m, 4H), 6.28 (s, 1H), 3.33 (t, J =5.3 Hz, 1H), 2.50 (s, 3H), 2.49 (s, 9H), 2.36 (s, 3H).^{13}C NMR (126 MHz, $CDCl_3$) δ 194.82, 160.13, 156.30, 145.92, 130.00, 129.45, 128.25, 122.10, 104.82, 52.63, 36.86, 29.19, 14.52.

1-(5-(trimethylamine-boranyl(m-tolyl))methyl)-2-methylfuran-3-yl)ethan-1-one (6e): light yellow oil, 91%. ^{1}H NMR (500 MHz, $CDCl_3$) δ 7.13-7.-7 (m, 3H), 6.88-6.86 (m, 1H), 6.29 (s, 1H), 3.31 (s, 1H), 2.50 (s, 3H), 2.46 (s, 9H), 2.35 (s, 3H), 2.30 (s, 3H). ^{13}C NMR (126 MHz, $CDCl_3$) δ 194.91, 160.93, 156.03, 147.20, 137.56, 128.91, 128.07, 125.32, 125.25, 122.12, 104.52, 77.36, 77.11, 76.85, 52.56, 37.40, 29.16, 21.57, 14.53.

1-(5-(trimethylamine-boranyl(3-methoxyphenyl)methyl)-2-methylfuran-3-yl)ethan-1-one (6f): light yellow oil, 77%, ^{1}H NMR (500 MHz, $CDCl_3$) δ 7.14 (t, J =7.9 Hz, 2H), 6.91-6.84 (m, 2H), 6.62 (dd, J =8.1, 1.7 Hz, 1H), 6.29 (s, 1H), 3.77 (s, 3H), 3.33 (t, J =5.2 Hz, 1H), 2.49 (s, 3H), 2.47 (s, 9H), 2.35 (s, 3H). ^{13}C NMR (126 MHz, $CDCl_3$) δ 194.95, 160.56, 159.53, 156.13, 149.08, 129.06, 122.06, 120.82, 114.15, 109.56, 104.63, 77.34, 77.08, 76.83, 55.08, 52.58, 37.75, 29.18, 14.54.

1-(5-(trimethylamine-boranyl(phenyl)methyl)-2-ethylfuran-3-yl)propan-1-one (6h): light yellow oil, 93%. ^{1}H NMR (500 MHz, $CDCl_3$) δ 7.29 (d, J =6.6 Hz, 2H), 7.22 (t, J =7.7 Hz, 2H), 7.06 (t, J =7.3 Hz, 1H),), 6.27 (s, 1H), 3.38 (t, J =5.3 Hz, 1H), 3.01-2.90 (m, 2H), 2.71 (q, J =7.3 Hz, 2H), 2.47 (s, 9H), 1.18 (t, J =7.5 Hz, 3H), 1.12 (t, J =7.3 Hz, 3H).^{13}C NMR (126 MHz, $CDCl_3$) δ 197.84, 161.01, 160.45, 147.31, 128.16, 124.42, 120.50, 103.95, 77.32, 77.07, 76.81, 52.52, 38.00, 34.29, 21.72, 12.37, 7.98.

(5-(trimethylamine-boranyl(phenyl)methyl)-2-phenylfuran-3-yl)(phenyl)methanone (6i):light yellow oil, 22%. ^{1}H NMR (500 MHz, $CDCl_3$) δ 7.82 (d, J =8.3 Hz, 2H), 7.67 (d, J =8.2 Hz, 2H), 7.47 (t, J =7.4 Hz, 1H), 7.39 – 7.32 (m, 4H), 7.29 – 7.17 (m, 5H), 7.12-7.06 (m, 1H), 6.38 (s, 1H), 3.50 (t, J =5.3 Hz, 1H), 2.48 (s, 9H). ^{13}C NMR (126 MHz, $CDCl_3$) 192.53, 161.96, 153.67, 147.07, 138.58, 132.48, 130.57, 129.77, 128.27, 128.24, 128.20, 128.14, 127.23, 124.54, 121.85, 108.38, 77.37, 77.11, 76.86, 52.55, 38.20.

2. NMR spectra of the products

1-(5-((dimethyl(phenyl)silyl)(phenyl)methyl)-2-methylfuran-3-yl)ethan-1-one(3a)

^{1}H NMR

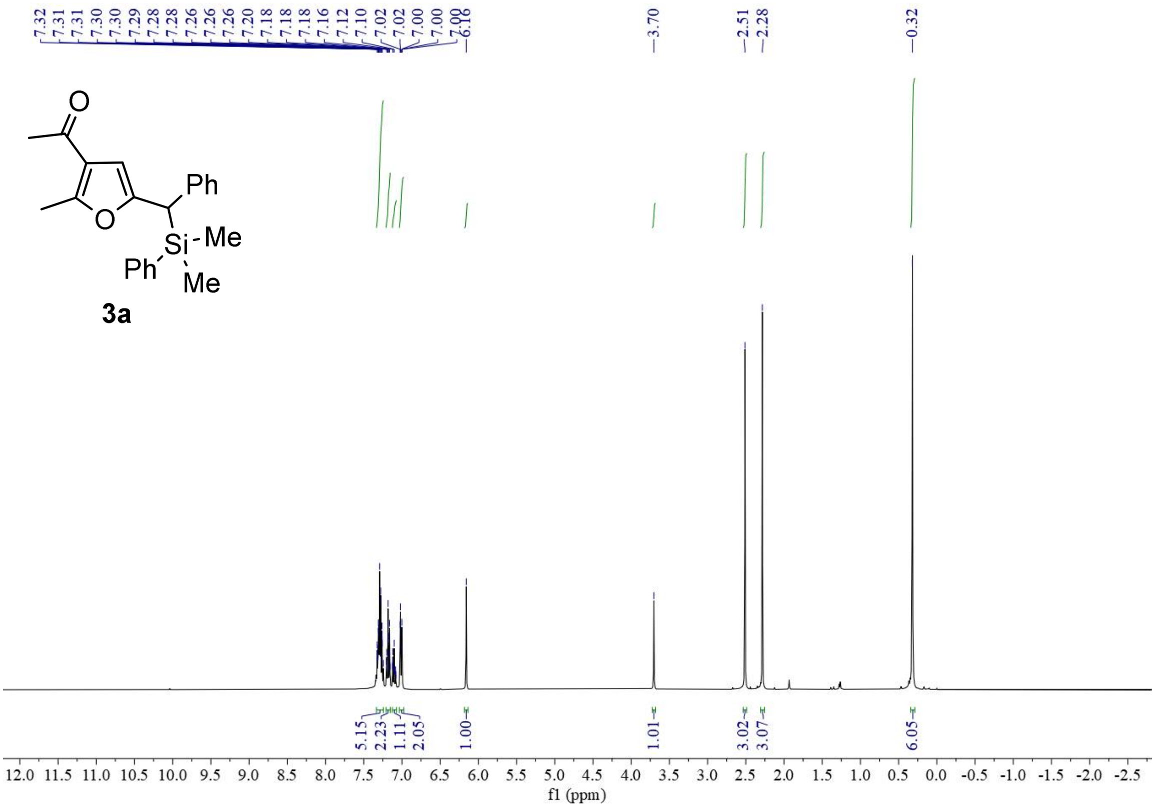

^{13}C NMR

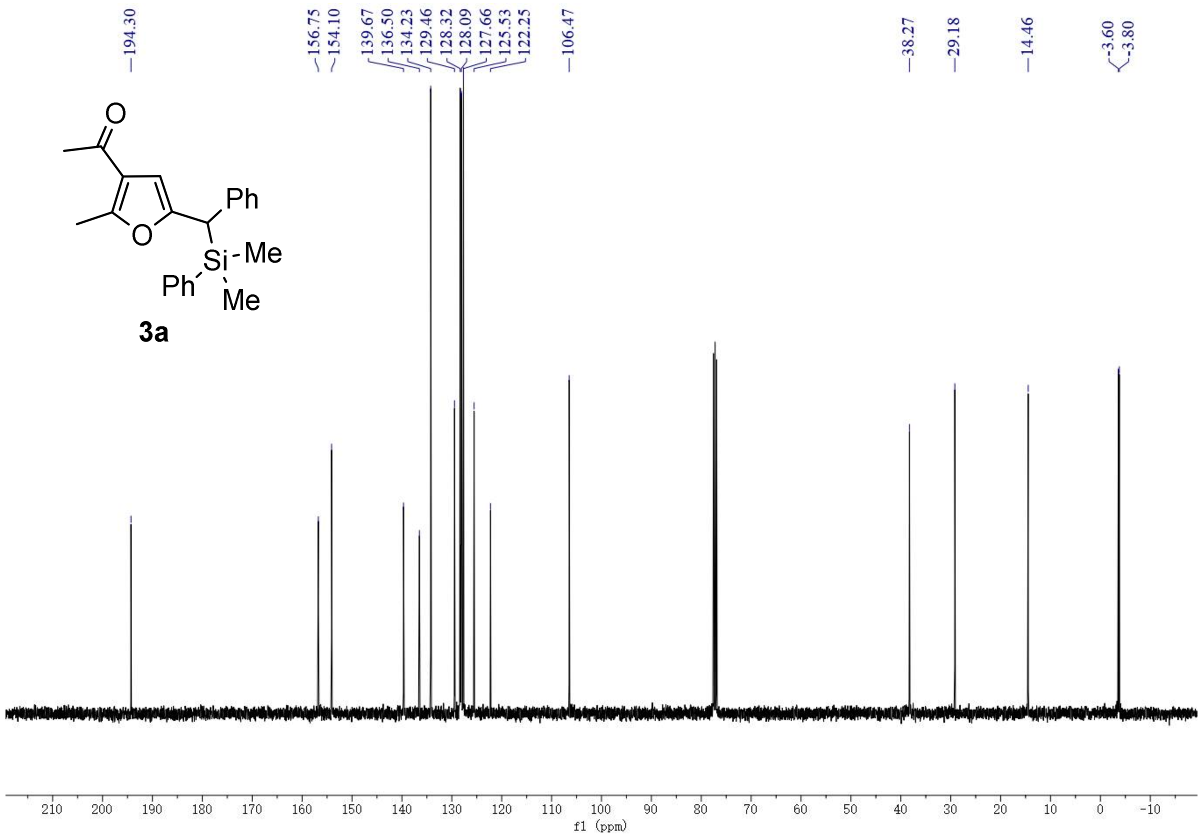

1-(2-methyl-5-((methyldiphenylsilyl)(phenyl)methyl)furan-3-yl)ethan-1-one (3b)

^{1}H NMR

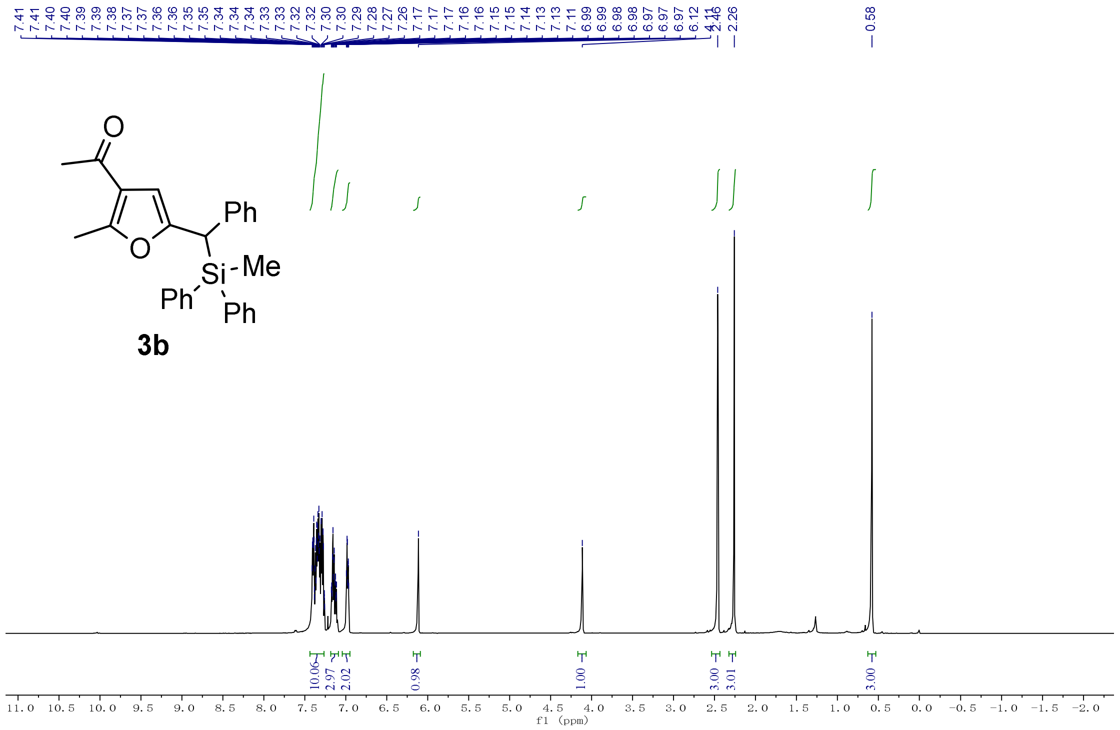

^{13}C NMR

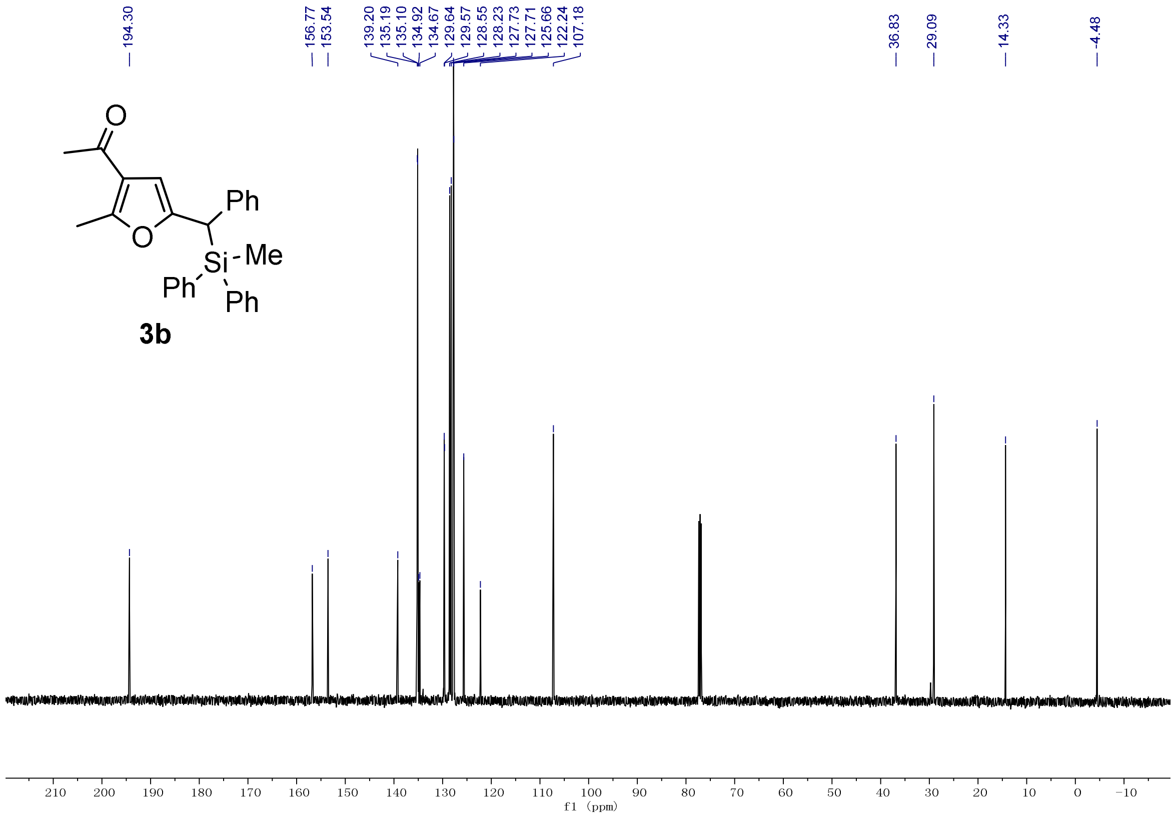

1-(2-methyl-5-(phenyl(triphenylsilyl)methyl)furan-3-yl)ethan-1-one (3c)

^{1}H NMR

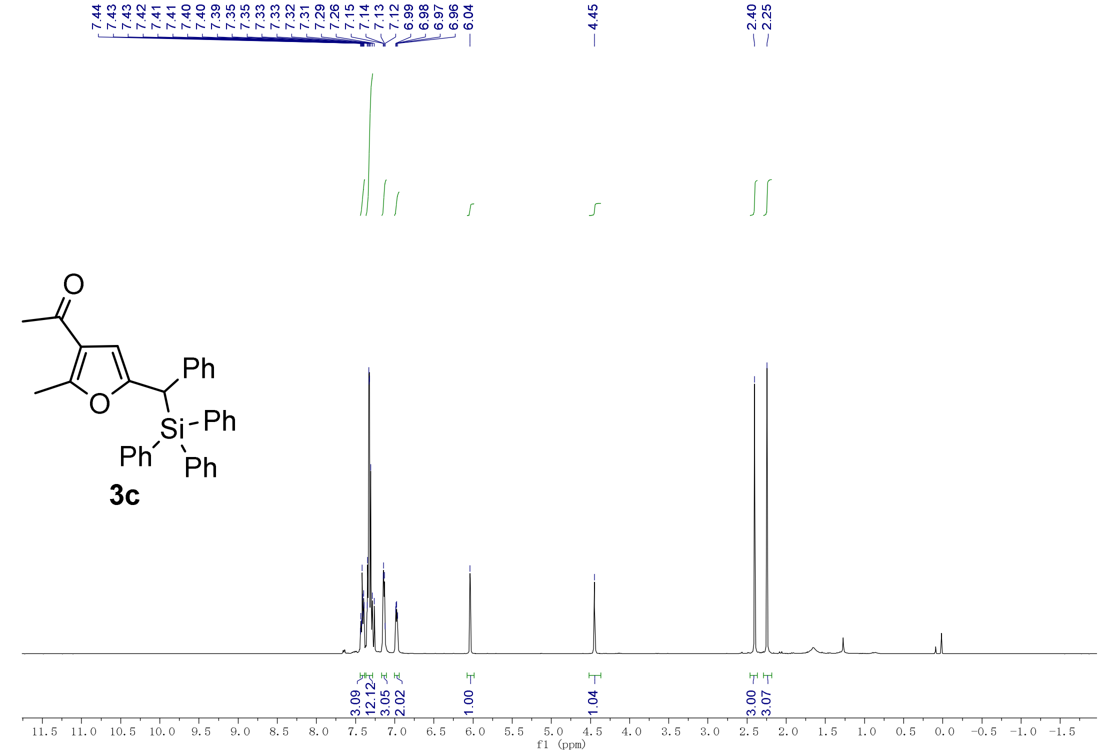
7.44 7.43 7.43 7.42 7.41 7.41 7.40 7.40 7.39 7.35 7.35 7.33 7.33 7.32 7.31 7.29 7.26 7.15 7.14 7.13 7.12 6.99 6.98 6.97 6.96 6.04
4.45
2.40 2.25
Ph
Si
Ph
Ph
Ph
O
3c
3.09 12.12 3.05 2.02 1.00 1.04 3.00 3.07
f1 (ppm)

^{13}C NMR

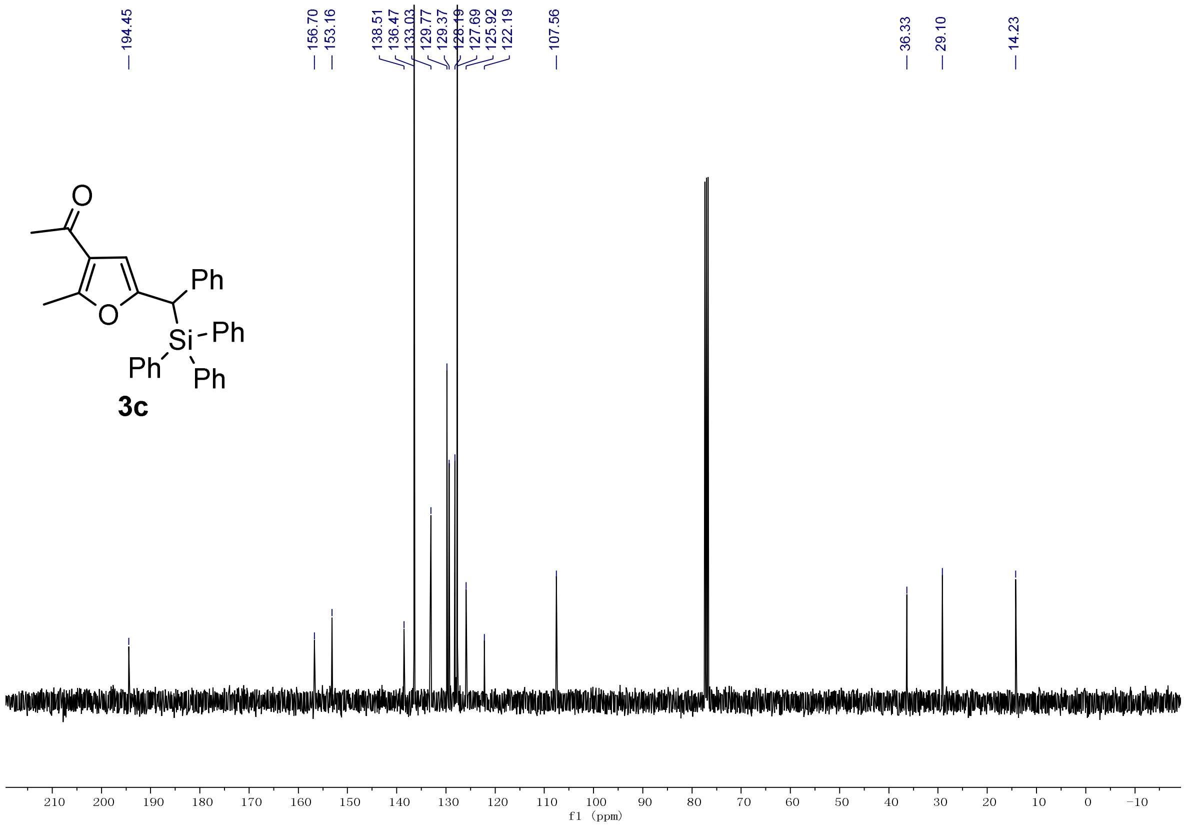
194.45
156.70 153.16
138.51 136.47 133.03 129.77 129.37 128.19 127.69 125.92 122.19
107.56
36.33 29.10 14.23
Ph
Si
Ph
Ph
Ph
O
3c
f1 (ppm)

1-(5-((benzyldimethylsilyl)(phenyl)methyl)-2-methylfuran-3-yl)ethan-1-one (3d)

^{1}H NMR

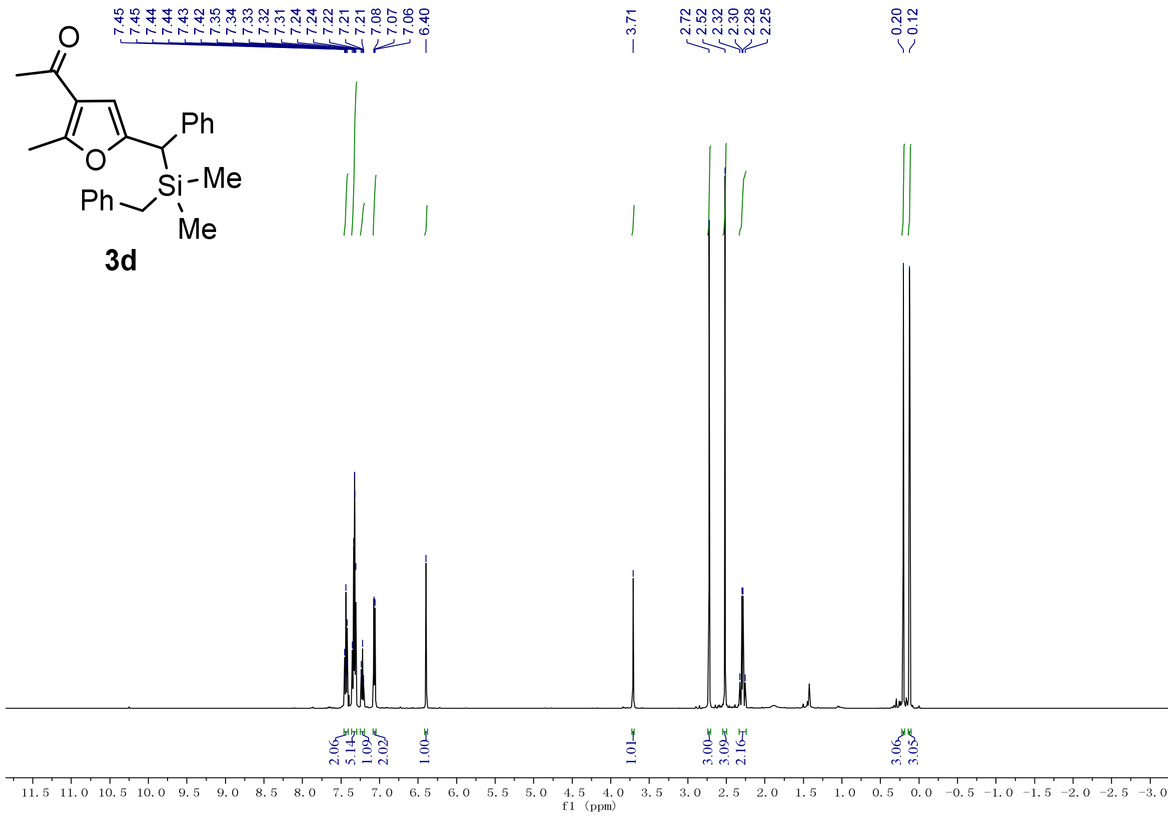

^{13}C NMR

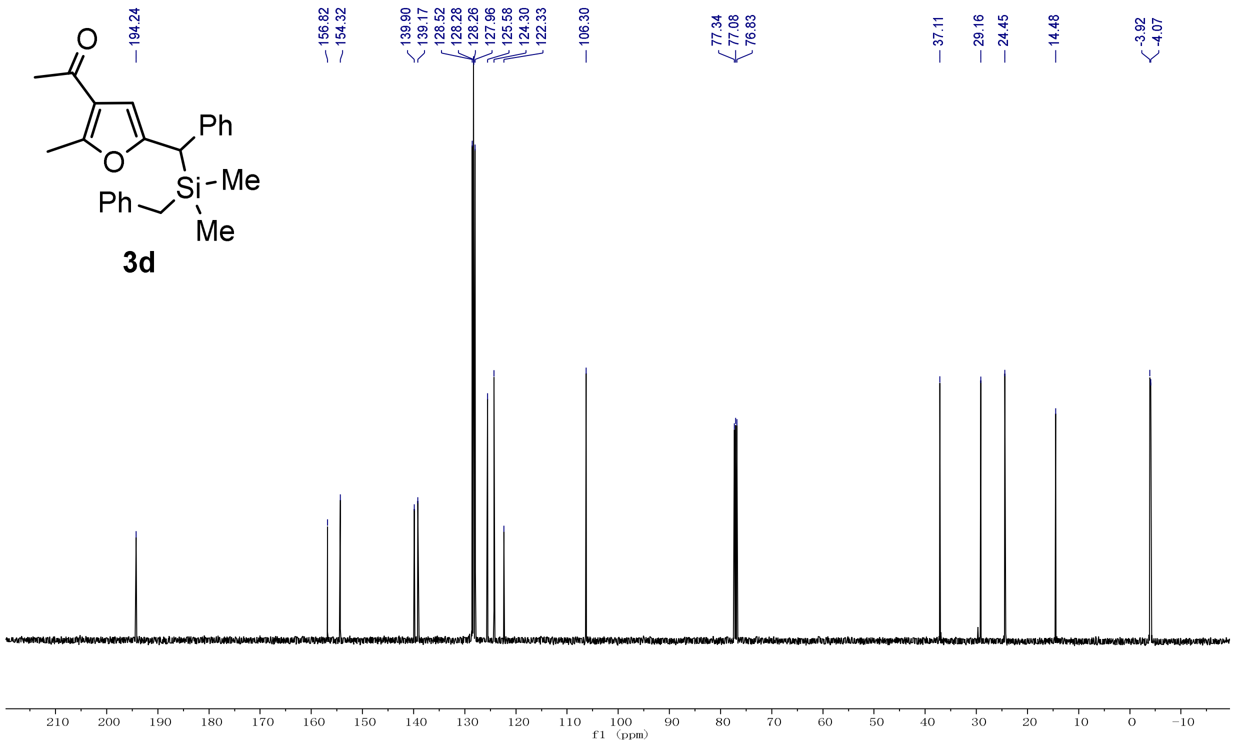

1-(2-methyl-5-(phenyl(triethylsilyl)methyl)furan-3-yl)ethan-1-one (3e)

^{1}H NMR

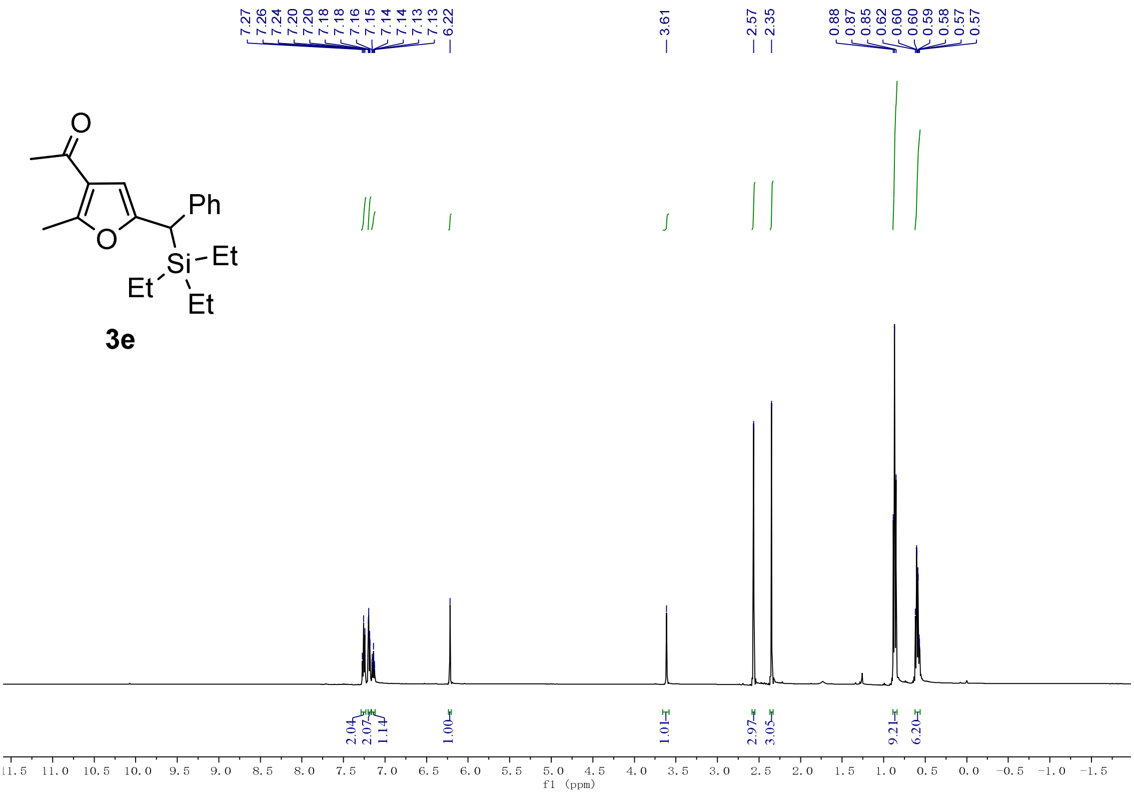

^{13}C NMR

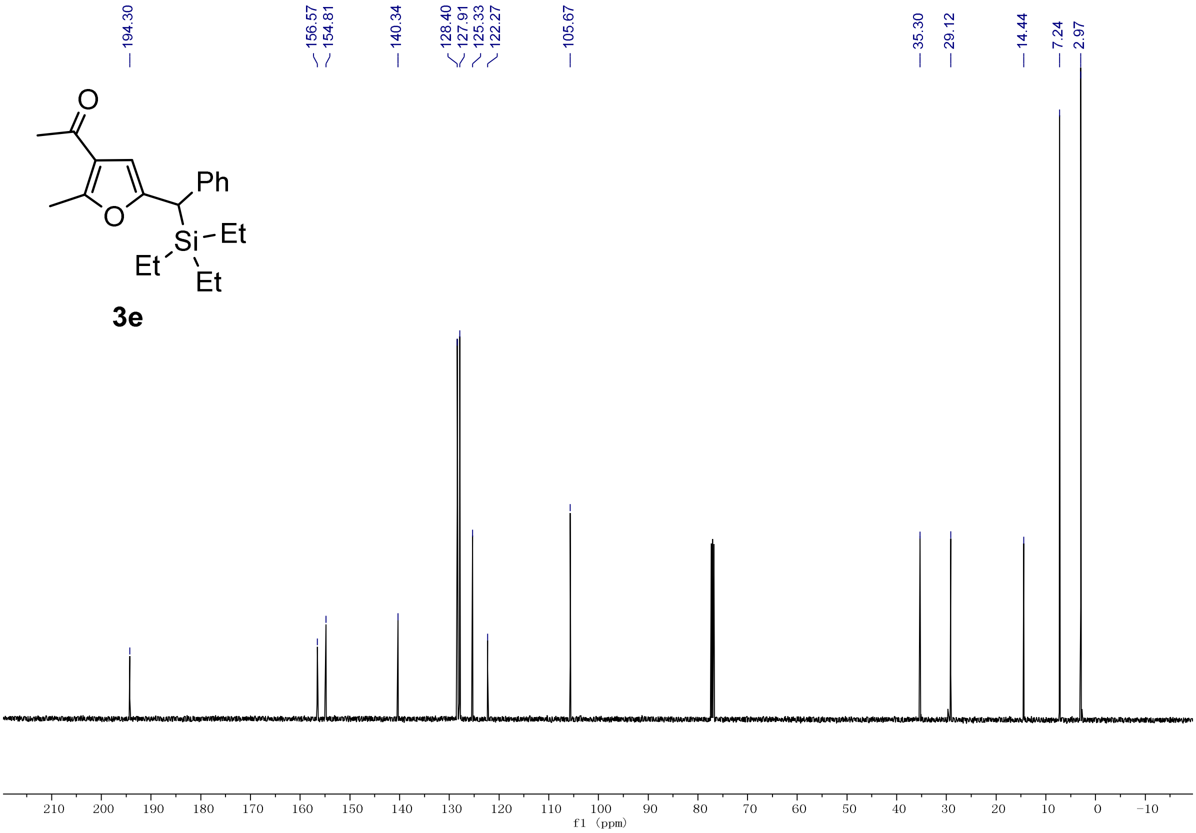

1-(2-methyl-5-(phenyl(tributylsilyl)methyl)furan-3-yl)ethan-1-one (3f).

^{1}H NMR

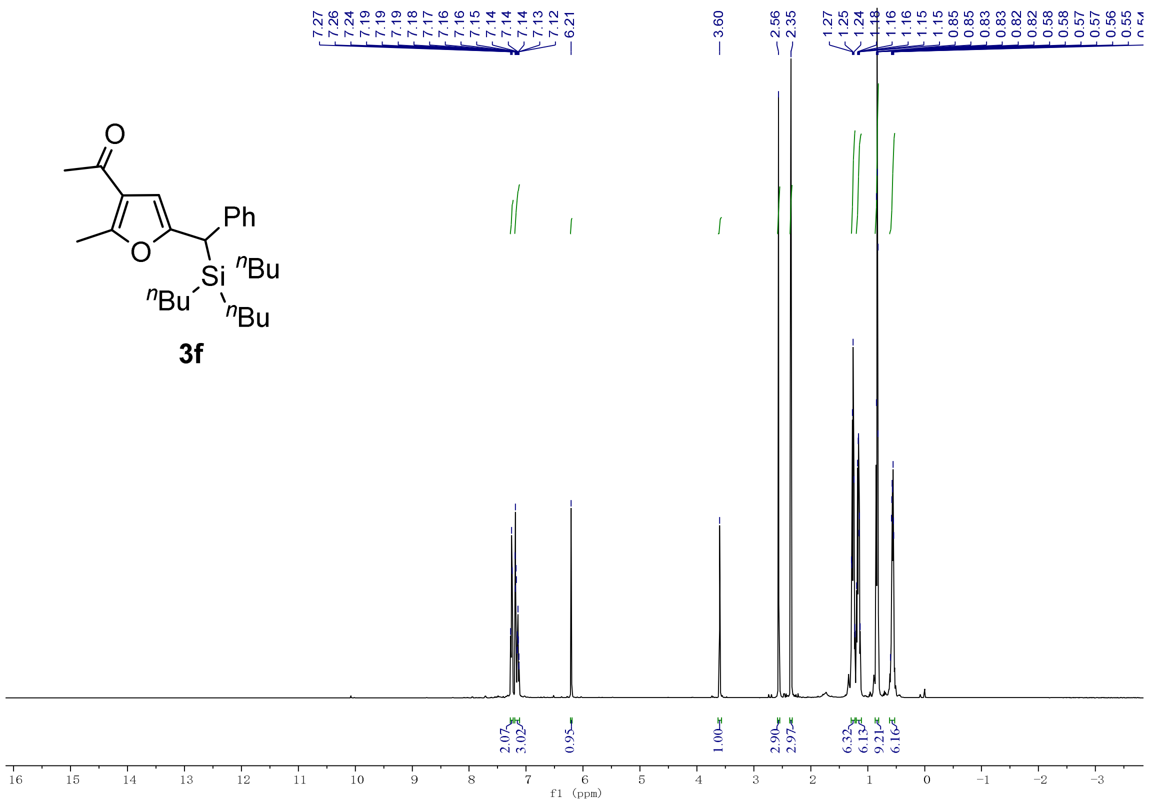

^{13}C NMR

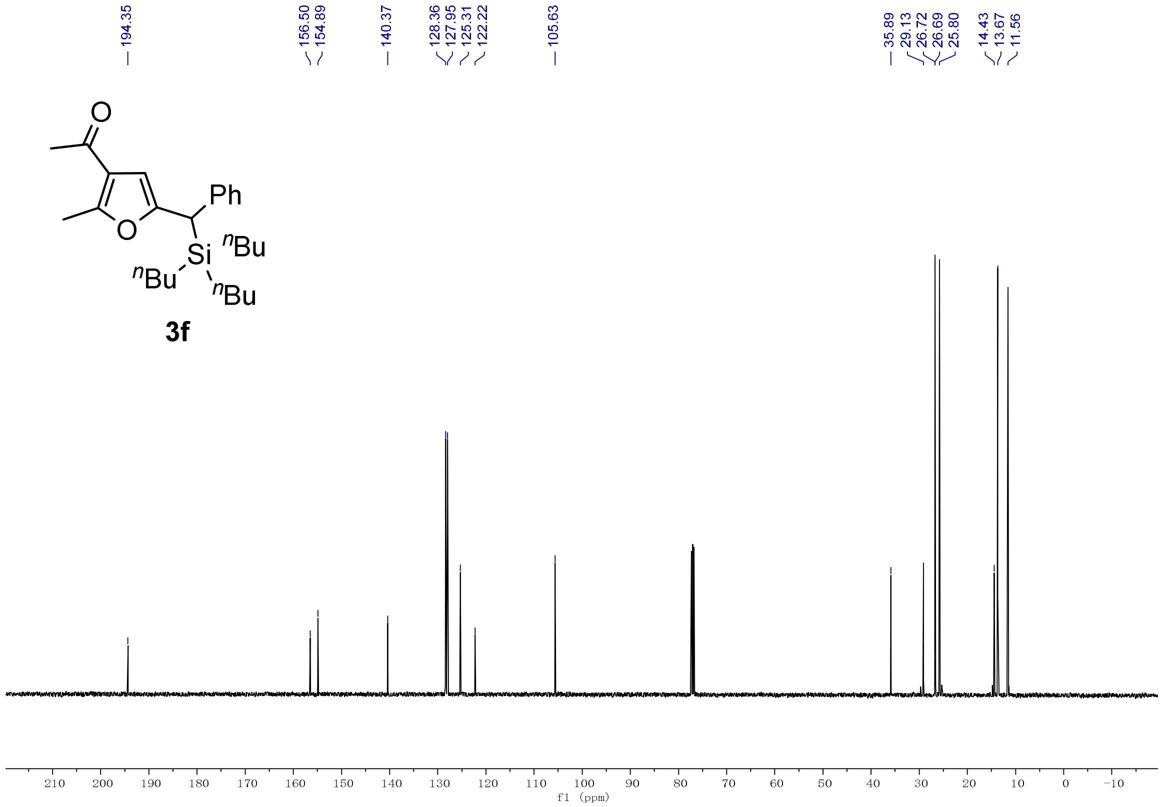

1-(5-((diphenylsilyl)(phenyl)methyl)-2-methylfuran-3-yl)ethan-1-one (3h).

^{1}H NMR

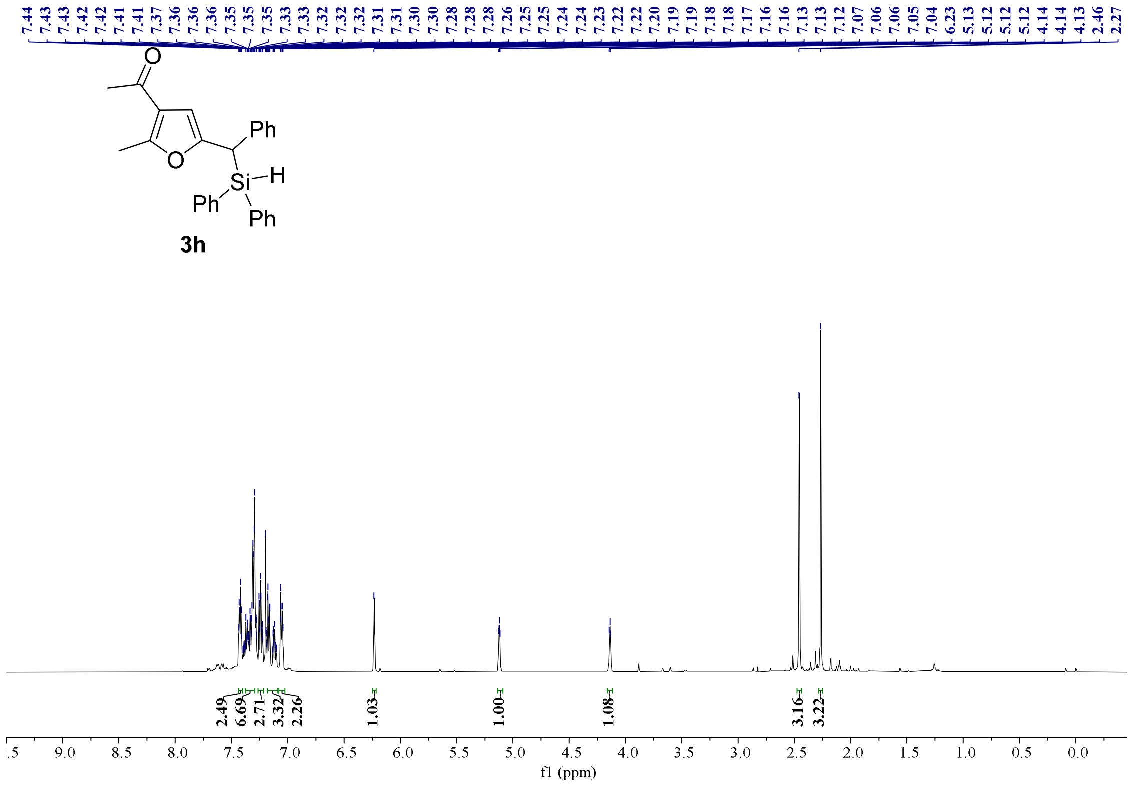

^{13}C NMR

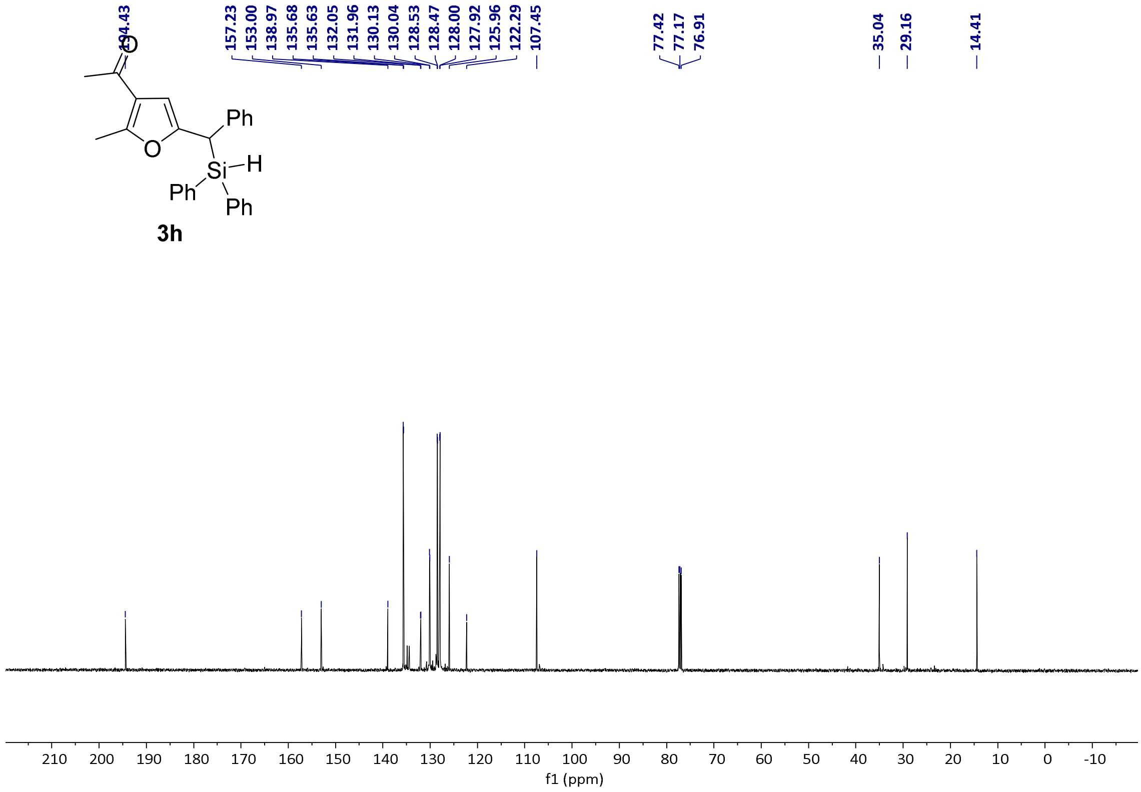

1-(5-((dimethyl(phenyl)silyl)(4-fluorophenyl)methyl)-2-methylfuran-3-yl)ethan-1-one (4a) .

^{1}H NMR

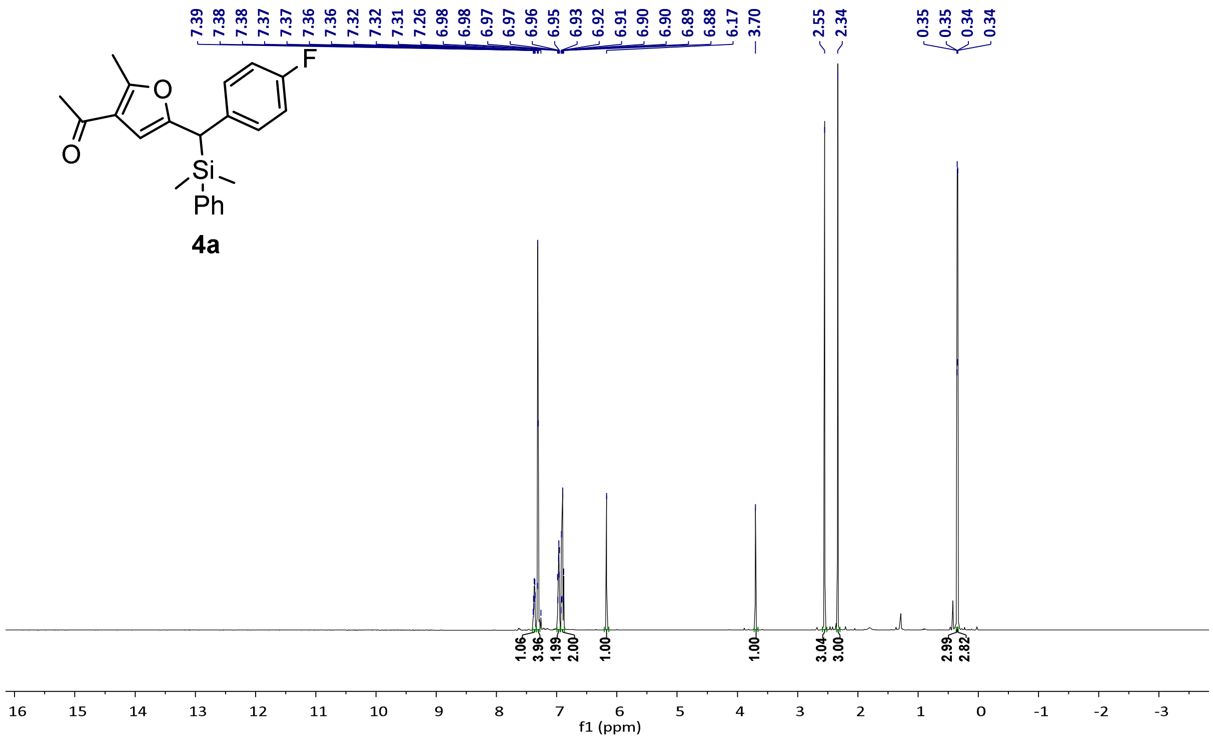

^{13}C NMR

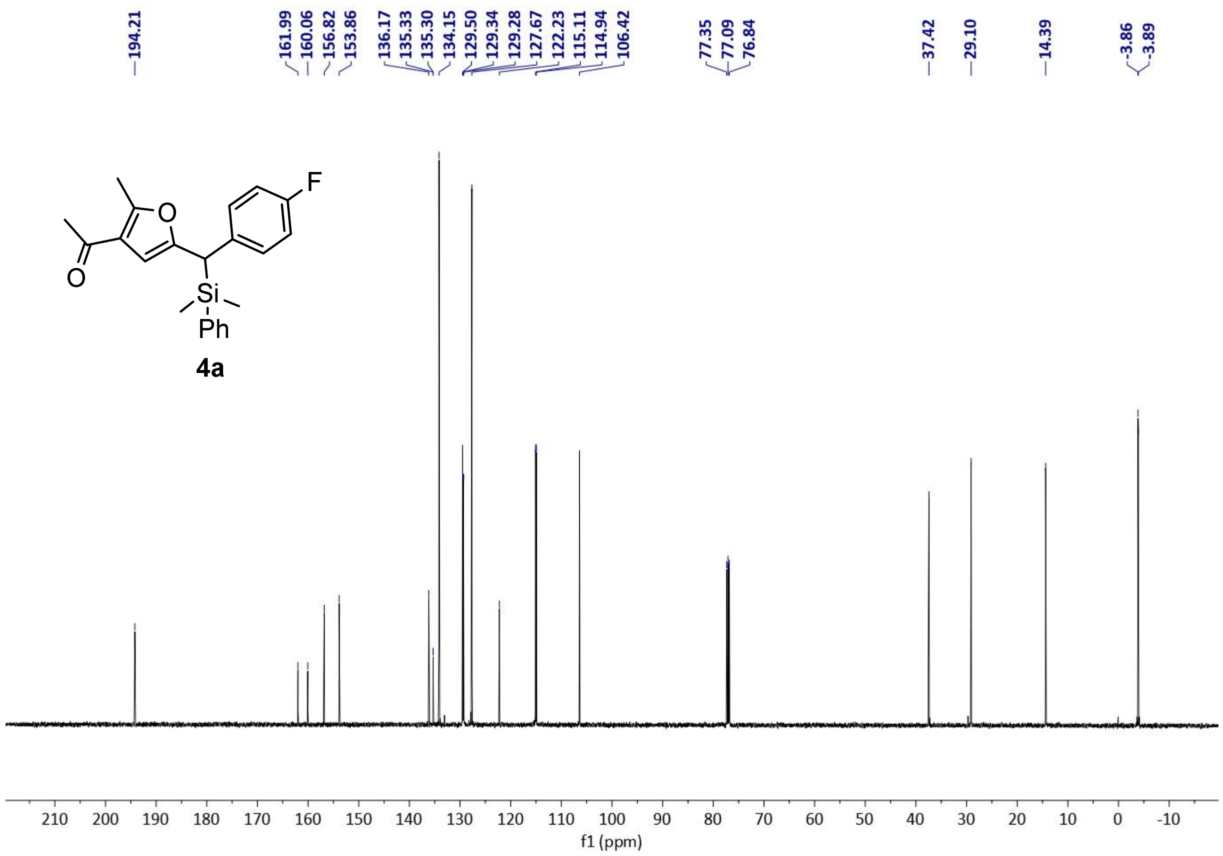

194.21
161.99
160.06
156.82
153.86
136.17
135.33
135.30
134.15
129.50
129.34
129.28
127.67
122.23
115.11
114.94
106.42
77.35
77.09
76.84
37.42
29.10
14.39
-3.86
-3.89
F
O
O
Si
Ph
4a
210 200 190 180 170 160 150 140 130 120 110 100 90 80 70 60 50 40 30 20 10 0 -10
f1 (ppm)

^{19}F NMR

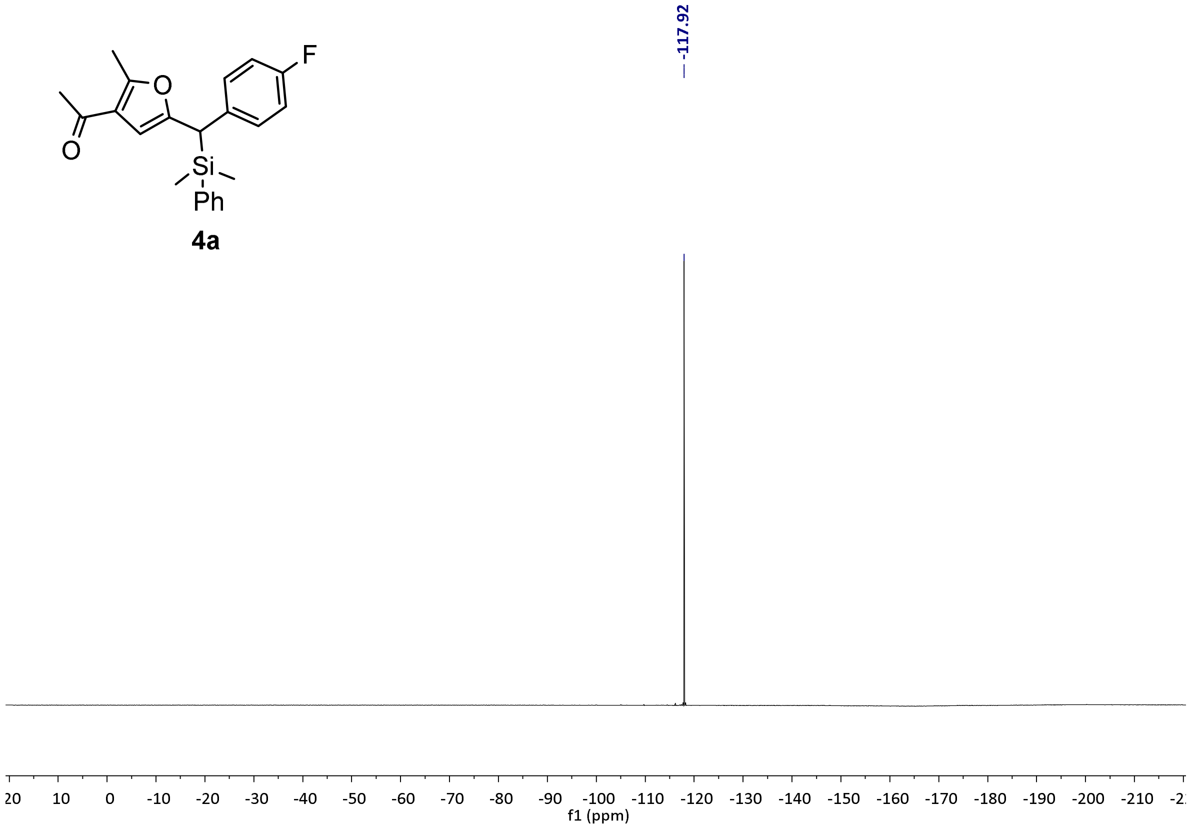

-117.92
F
O
O
Si
Ph
4a
20 10 0 -10 -20 -30 -40 -50 -60 -70 -80 -90 -100 -110 -120 -130 -140 -150 -160 -170 -180 -190 -200 -210
f1 (ppm)

1-(5-((4-chlorophenyl)(dimethyl(phenyl)silyl)methyl)-2-methylfuran-3-yl)ethan-1-one (4b)

^{1}H NMR

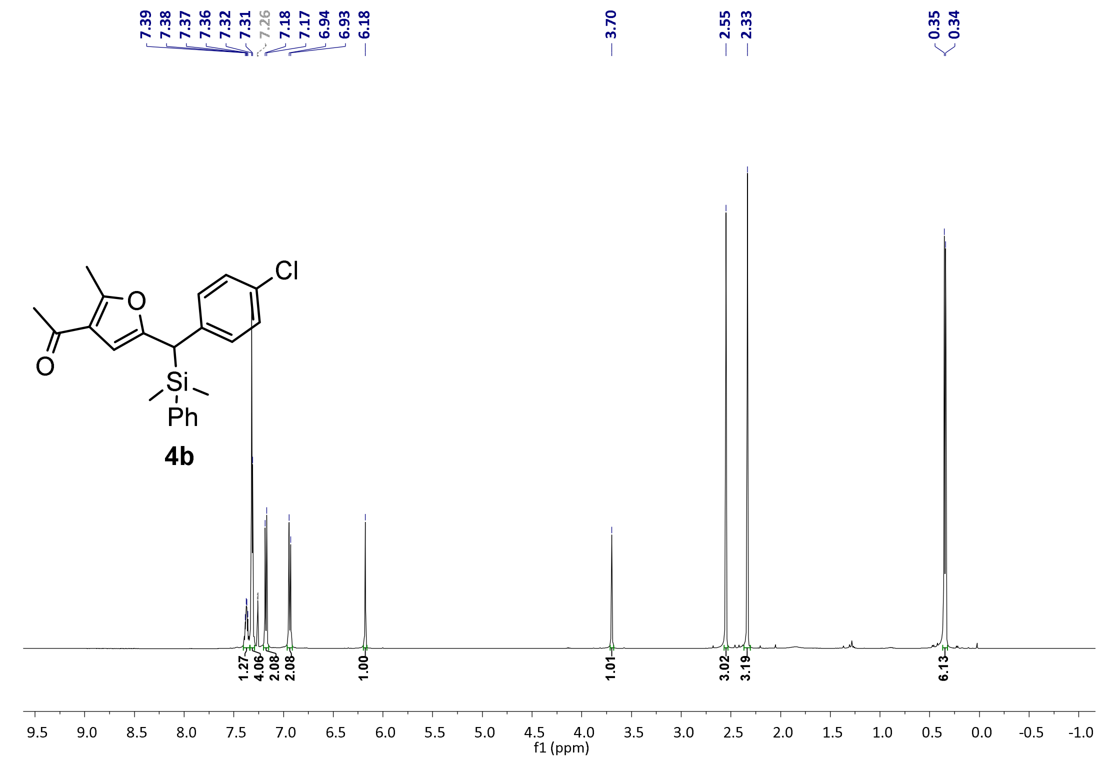
7.39 7.38 7.37 7.36 7.32 7.31 7.26 7.18 7.17 6.94 6.93 6.18
3.70
2.55 2.33
0.35 0.34
Cl
O
Si
Ph
4b
1.27 4.06 2.08 2.08 1.00 1.01 3.02 3.19 6.13
9.5 9.0 8.5 8.0 7.5 7.0 6.5 6.0 5.5 5.0 4.5 4.0 3.5 3.0 2.5 2.0 1.5 1.0 0.5 0.0 -0.5 -1.0
f1 (ppm)

^{13}C NMR

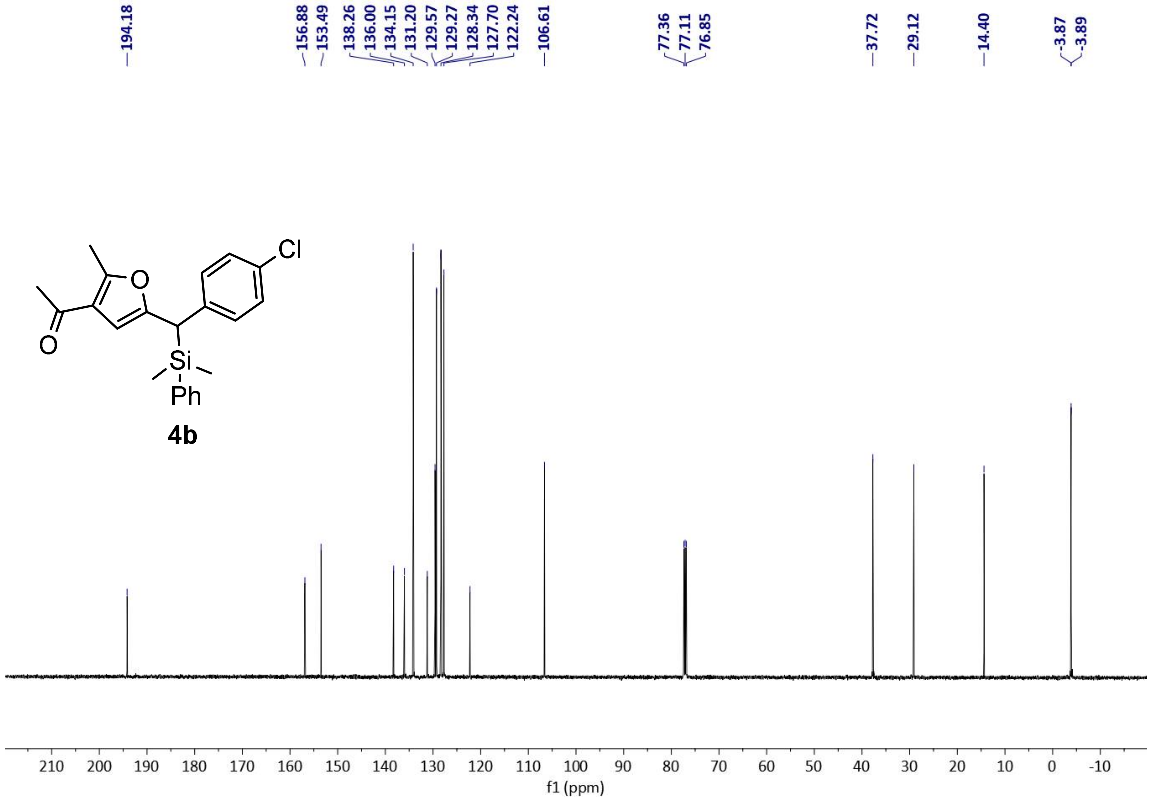
194.18
156.88 153.49 138.26 136.00 134.15 131.20 129.57 129.27 128.34 127.70 122.24 106.61
77.36 77.11 76.85
37.72 29.12 14.40 -3.87 -3.89
Cl
O
Si
Ph
4b
210 200 190 180 170 160 150 140 130 120 110 100 90 80 70 60 50 40 30 20 10 0 -10
f1 (ppm)

1-(5-((dimethyl(phenyl)silyl)(4-(trifluoromethyl)phenyl)methyl)-2-methylfuran-3-yl)ethan-1-one (4c)

^{1}H NMR

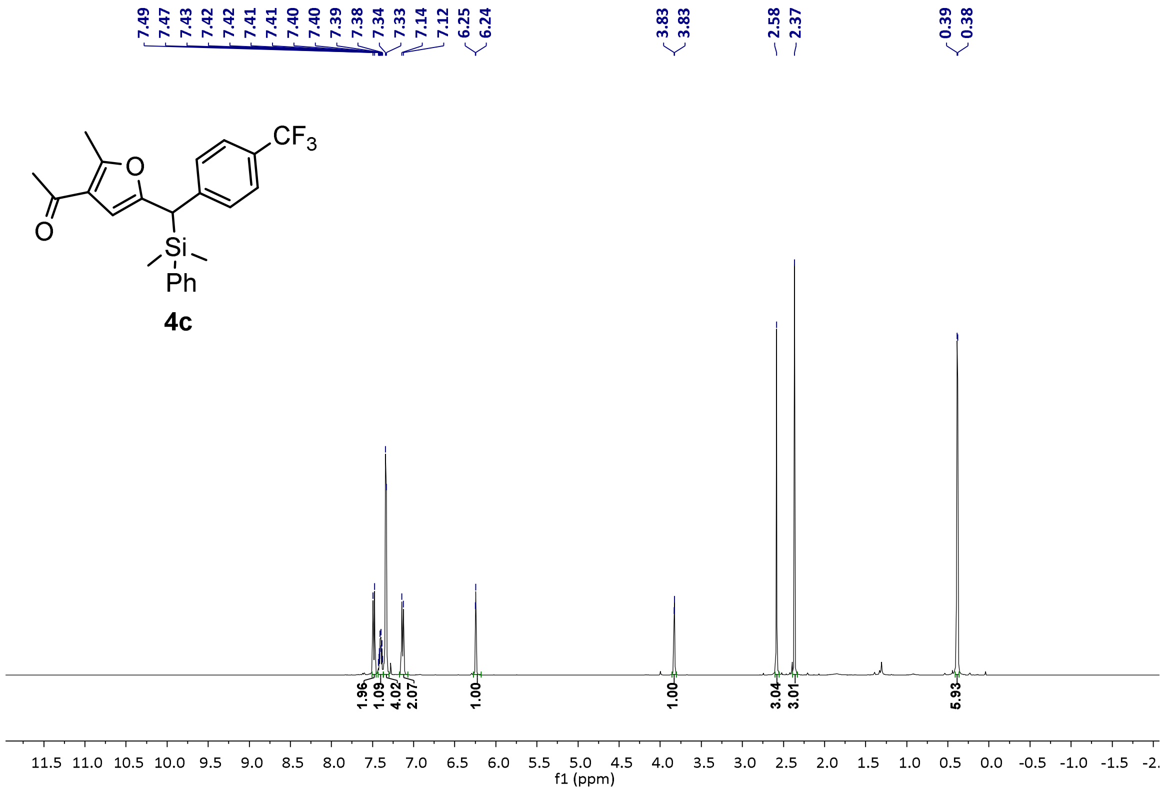

^{13}C NMR

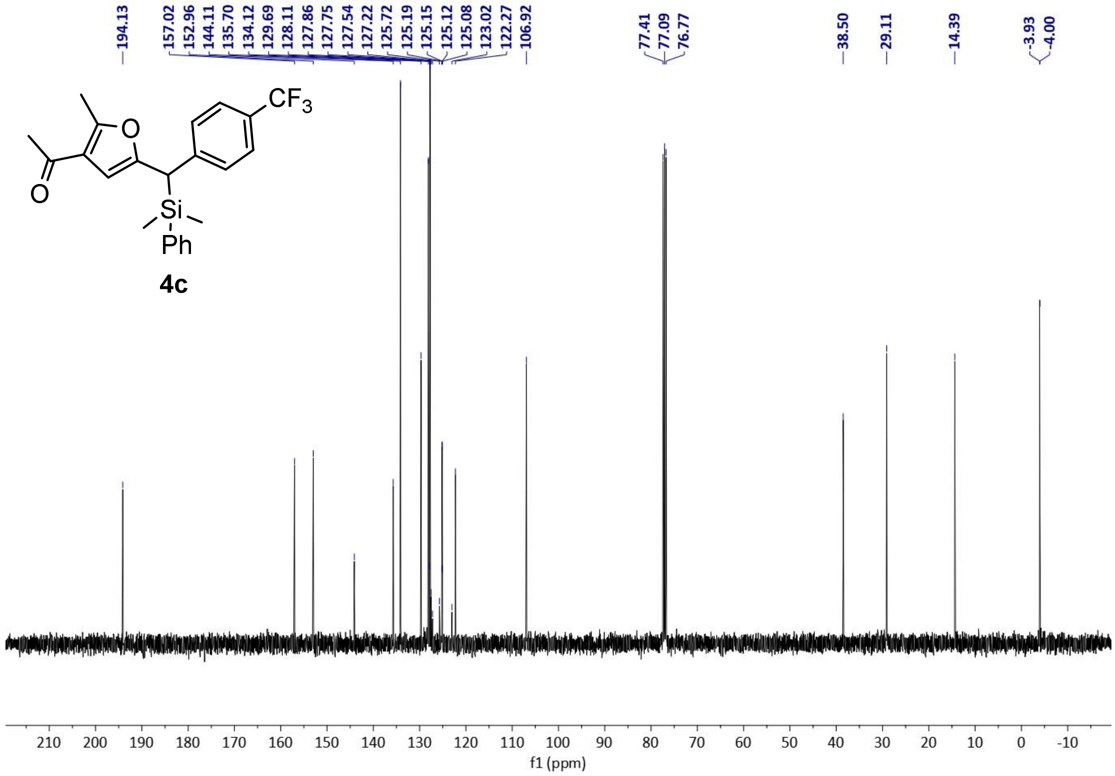

^{19}F NMR

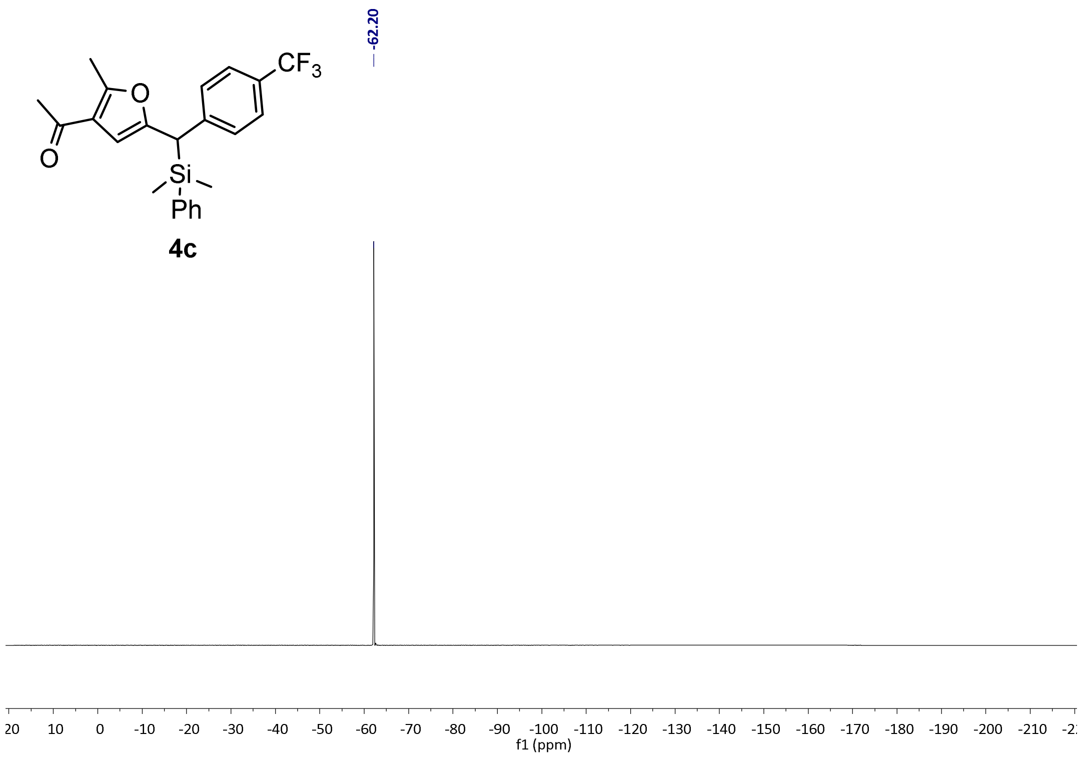

CF3
O
O
Si
Ph
4c
-62.20
20 10 0 -10 -20 -30 -40 -50 -60 -70 -80 -90 -100 -110 -120 -130 -140 -150 -160 -170 -180 -190 -200 -210
f1 (ppm)

1-(5-((dimethyl(phenyl)silyl)(*p*-tolyl)methyl)-2-methylfuran-3-yl)ethan-1-one (4d)

^{1}H NMR

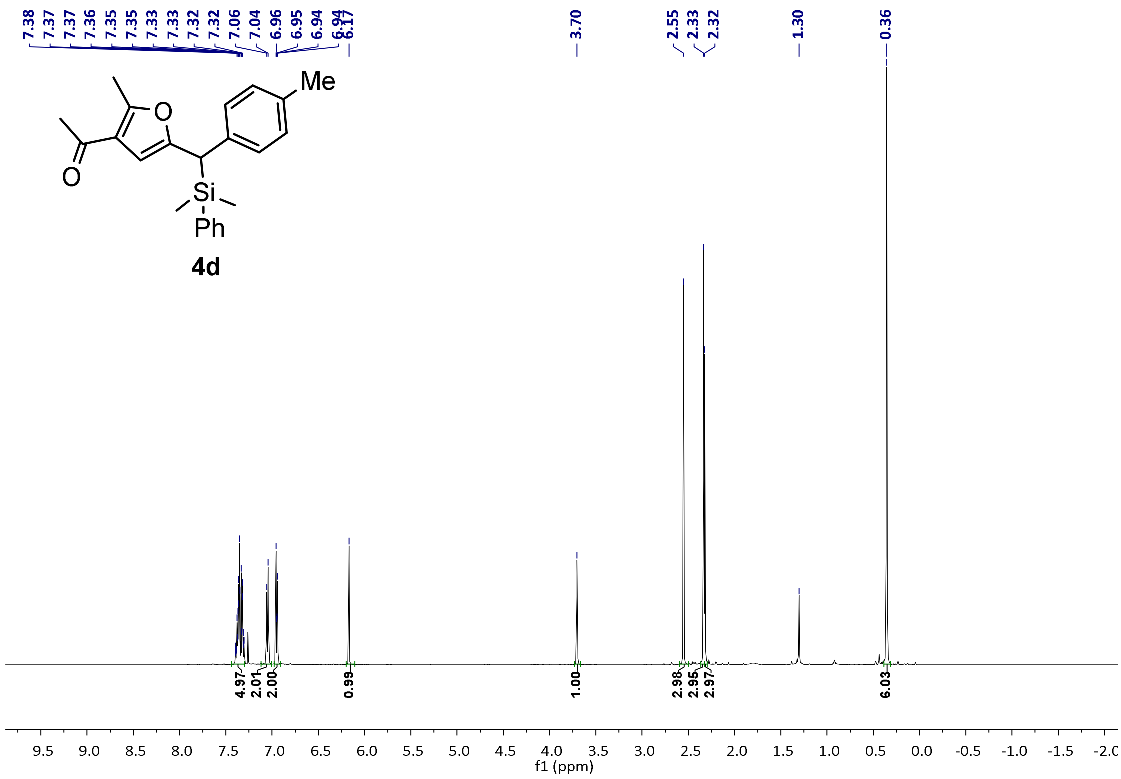

^{13}C NMR

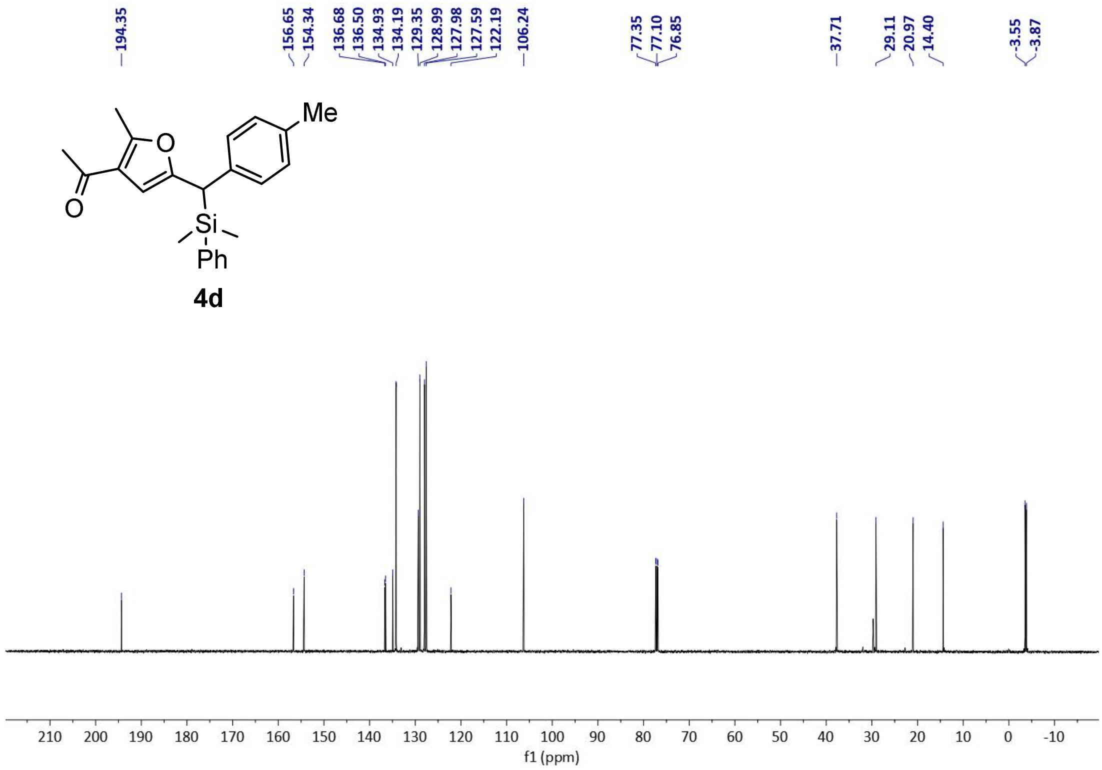

1-(5-((dimethyl(phenyl)silyl)(m-tolyl)methyl)-2-methylfuran-3-yl)ethan-1-one (4e)

^{1}H NMR

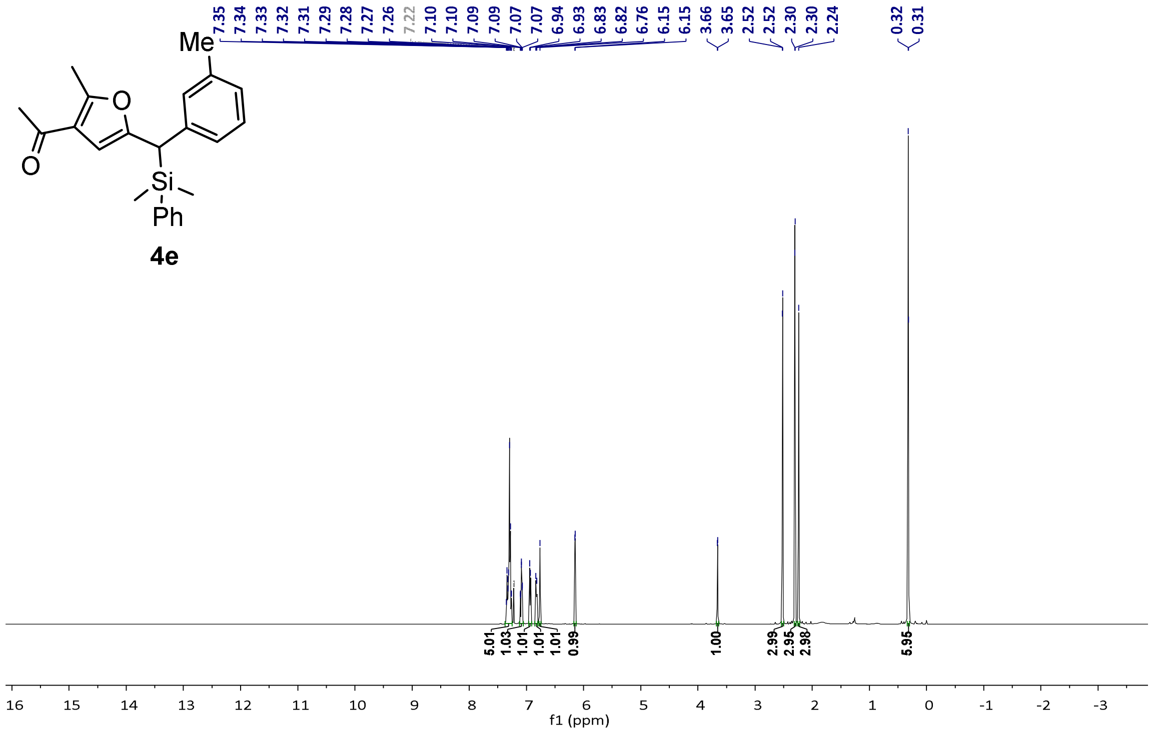

^{13}C NMR

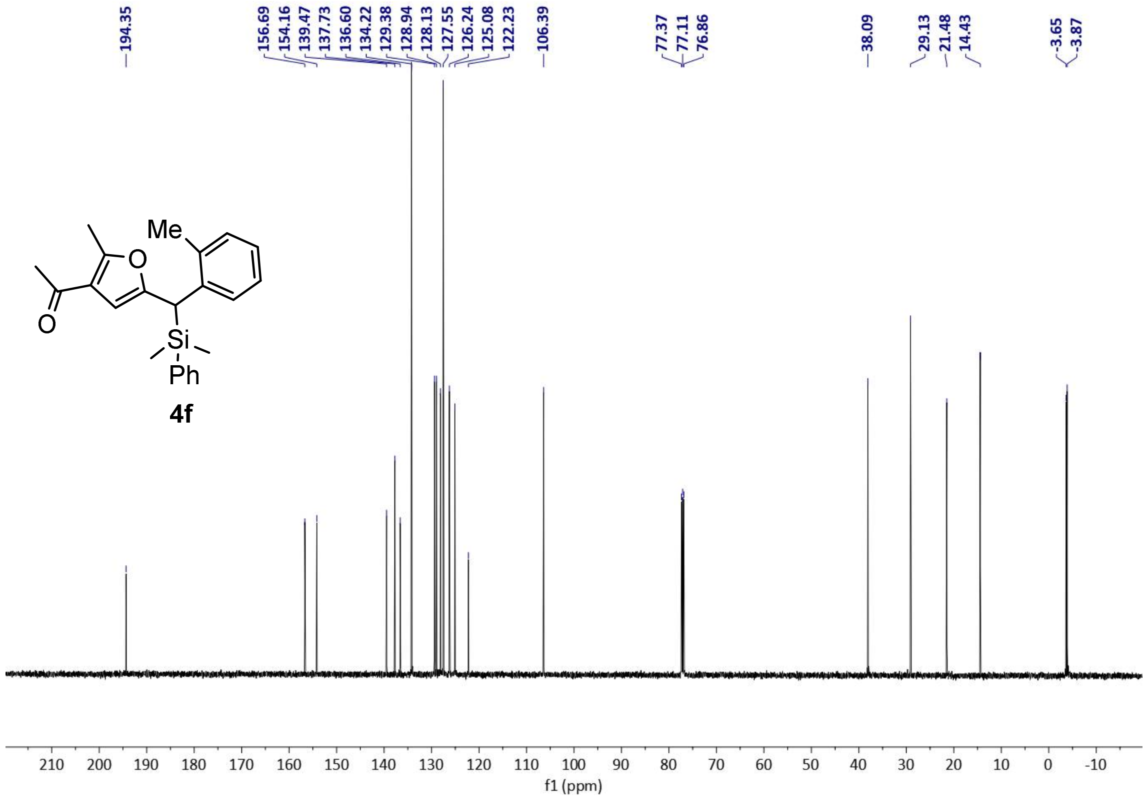

1-(5-((dimethyl(phenyl)silyl)(*o*-tolyl)methyl)-2-methylfuran-3-yl)ethan-1-one (4f)

^{1}H NMR

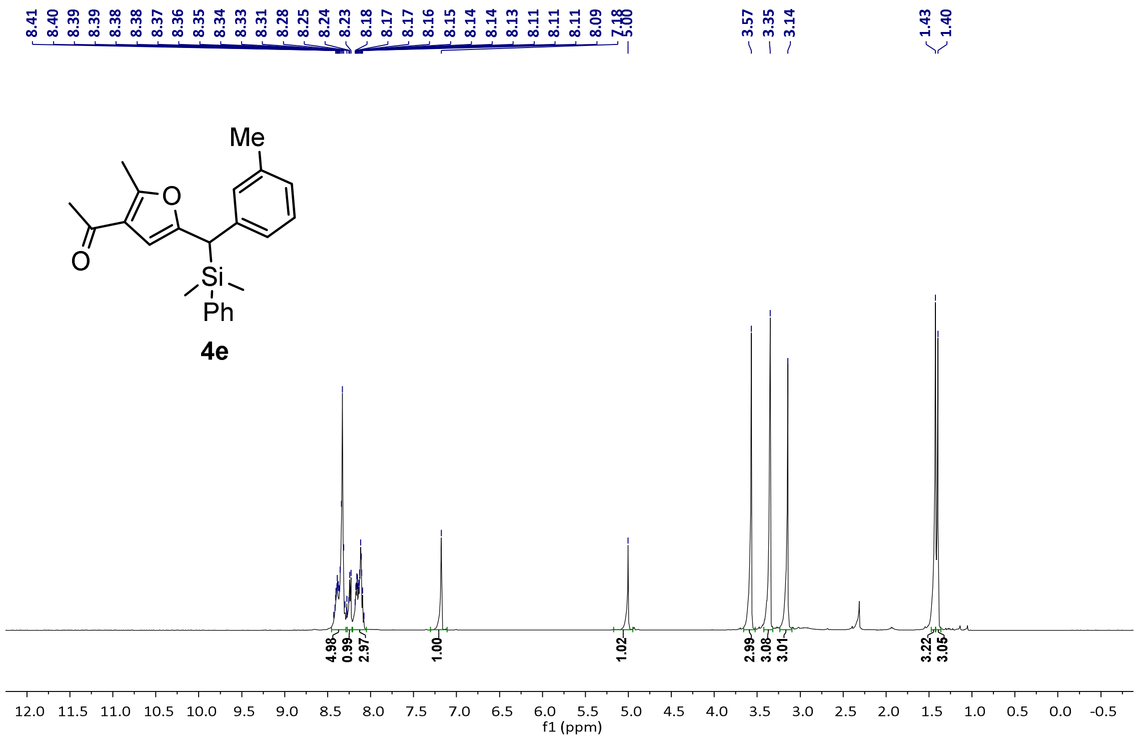

^{13}C NMR

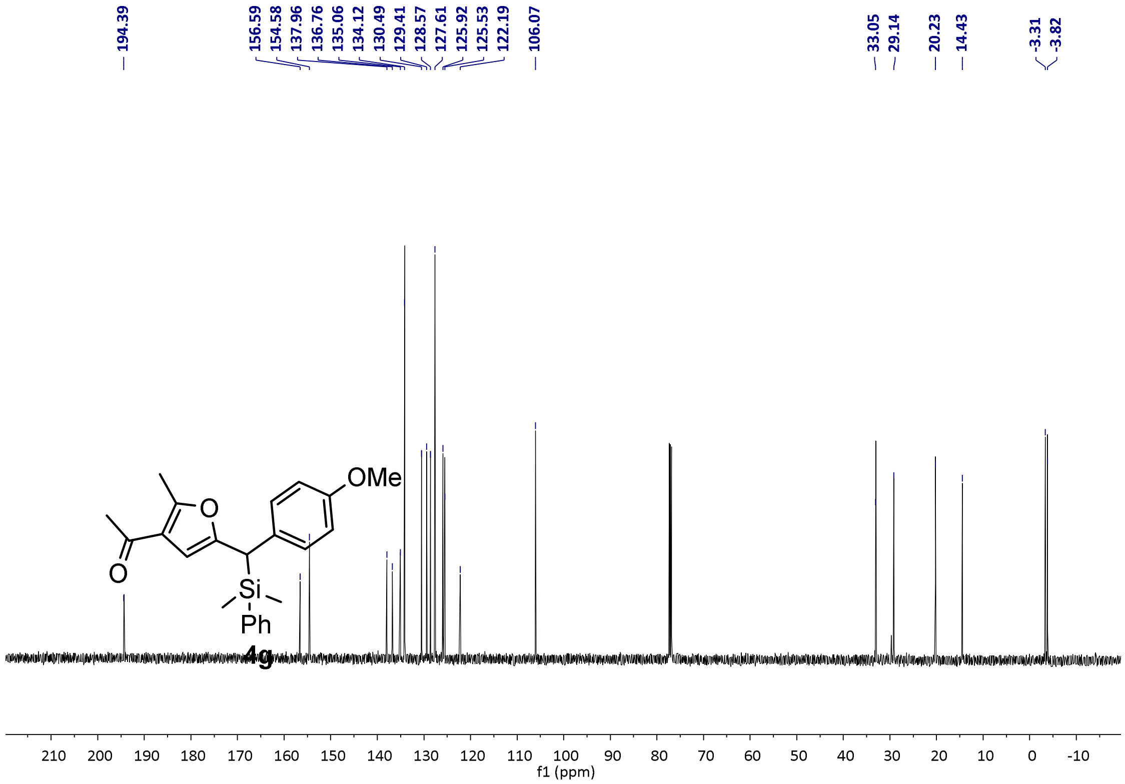

1-(5-((dimethyl(phenyl)silyl)(4-methoxyphenyl)methyl)-2-methylfuran-3-yl)ethan-1-one (4g)

^{1}H NMR

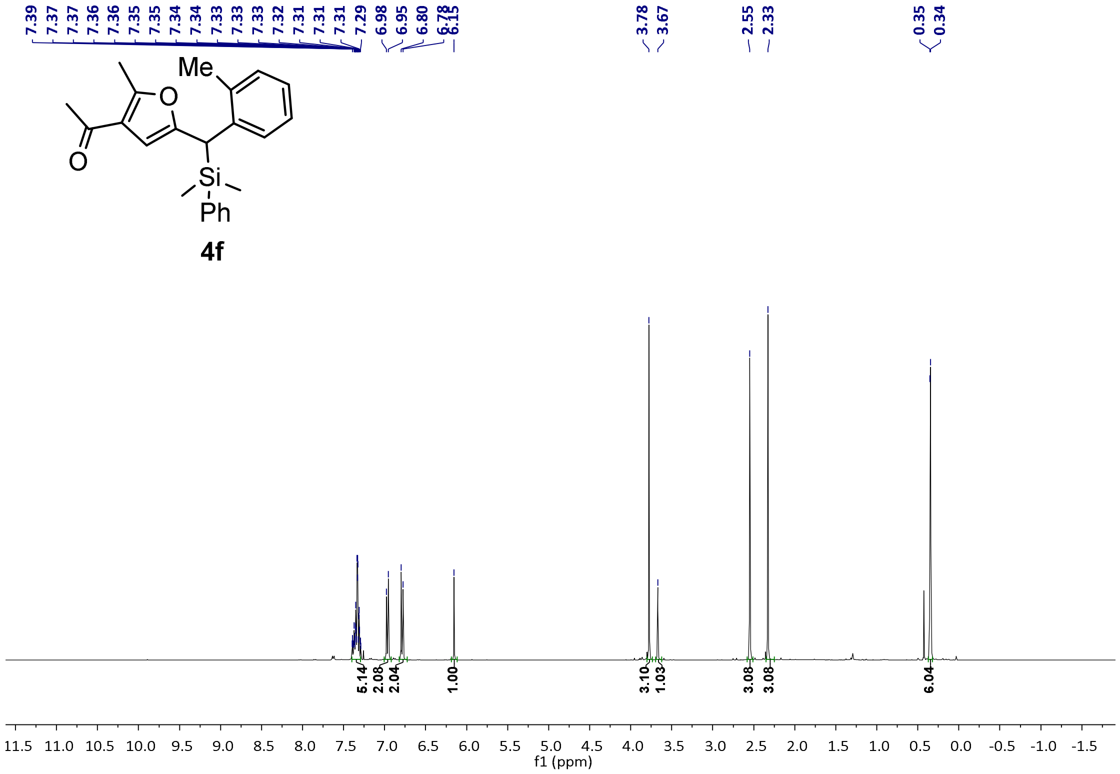

^{13}C NMR

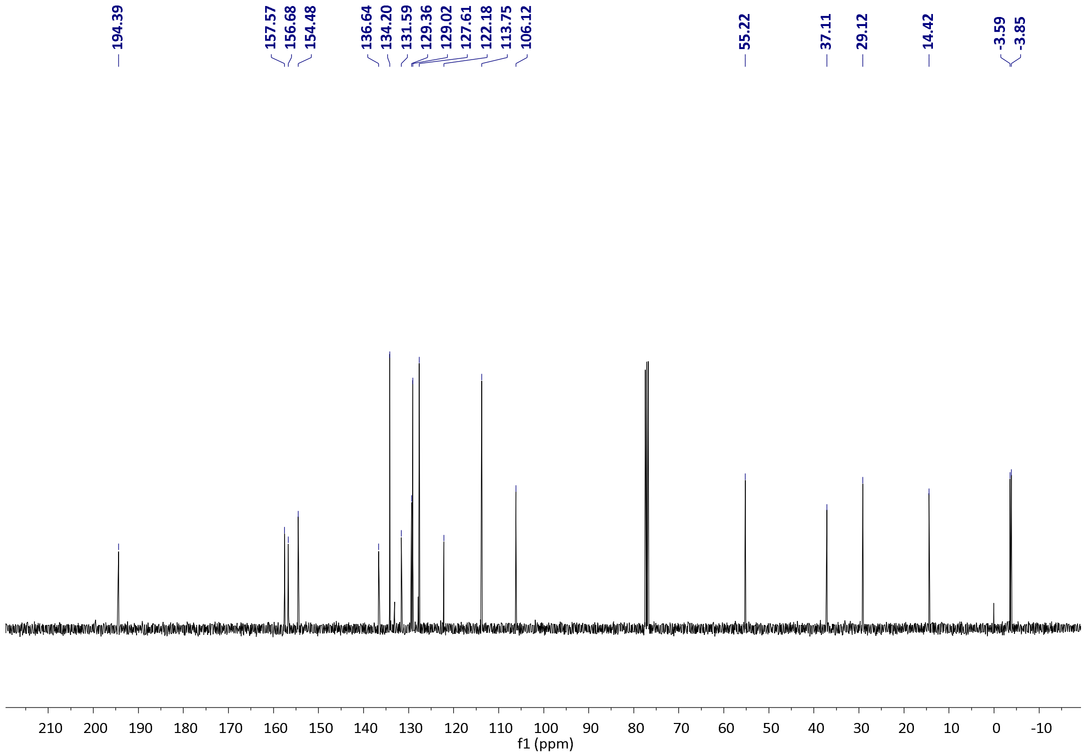

1-(5-((dimethyl(phenyl)silyl)(3-methoxyphenyl)methyl)-2-methylfuran-3-yl)ethan-1-one (4h)

^{1}H NMR

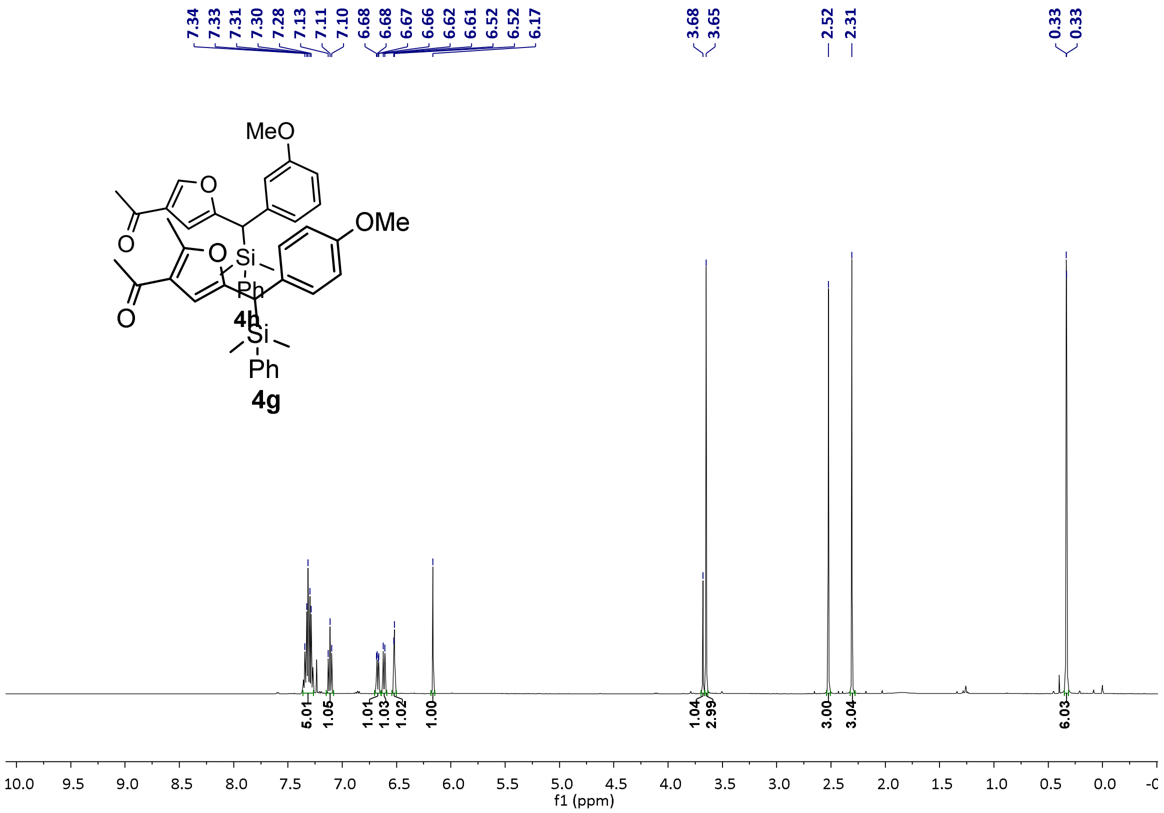

^{13}C NMR

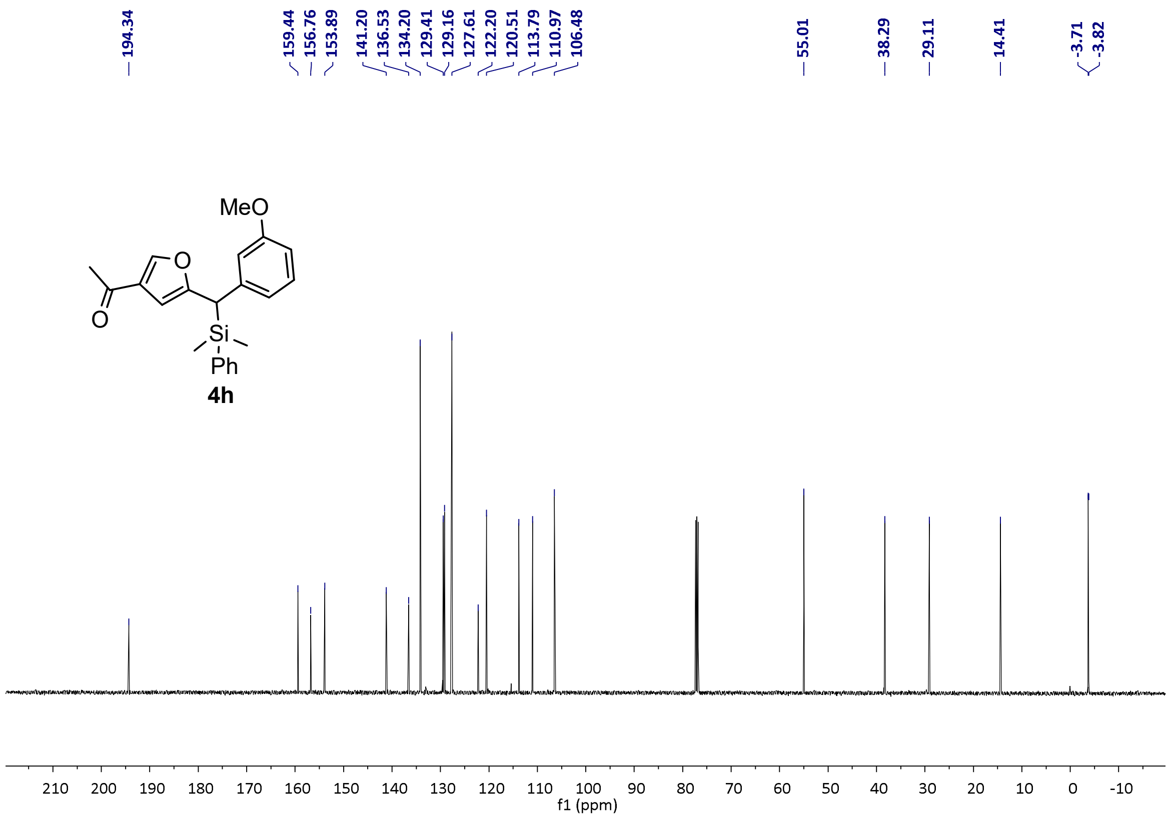

1-(5-([1,1'-biphenyl]-4-yl(dimethyl(phenyl)silyl)methyl)-2-methylfuran-3-yl)ethan-1-one (4i)

^{1}H NMR

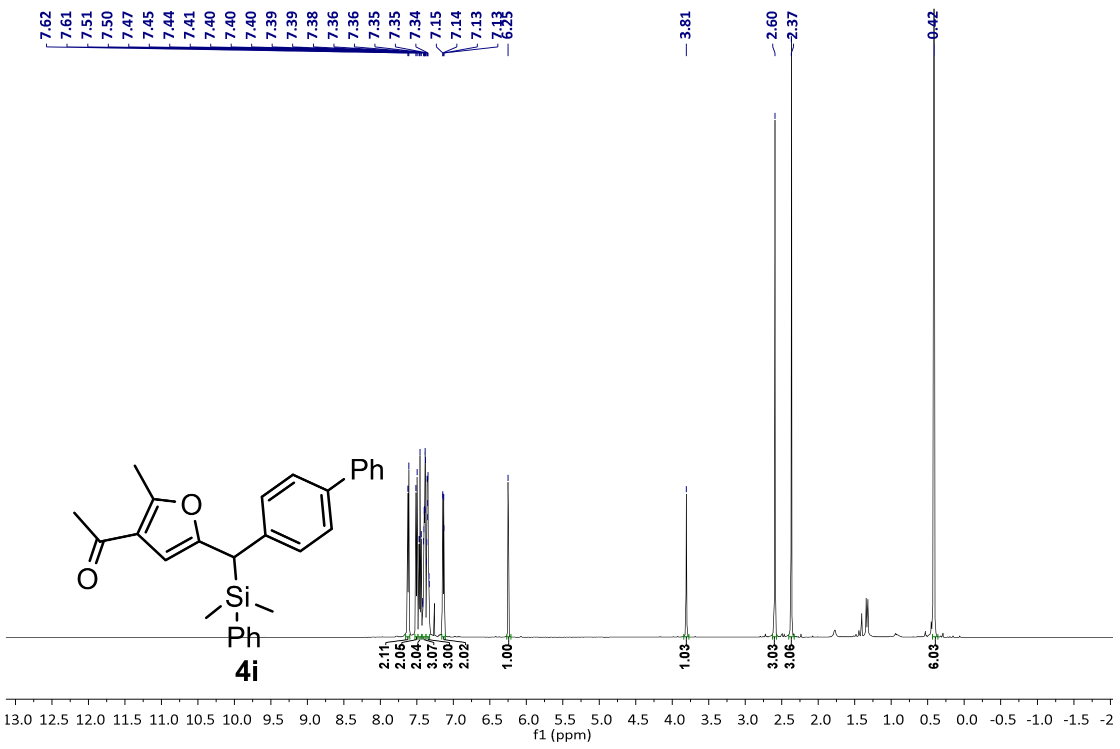

^{13}C NMR

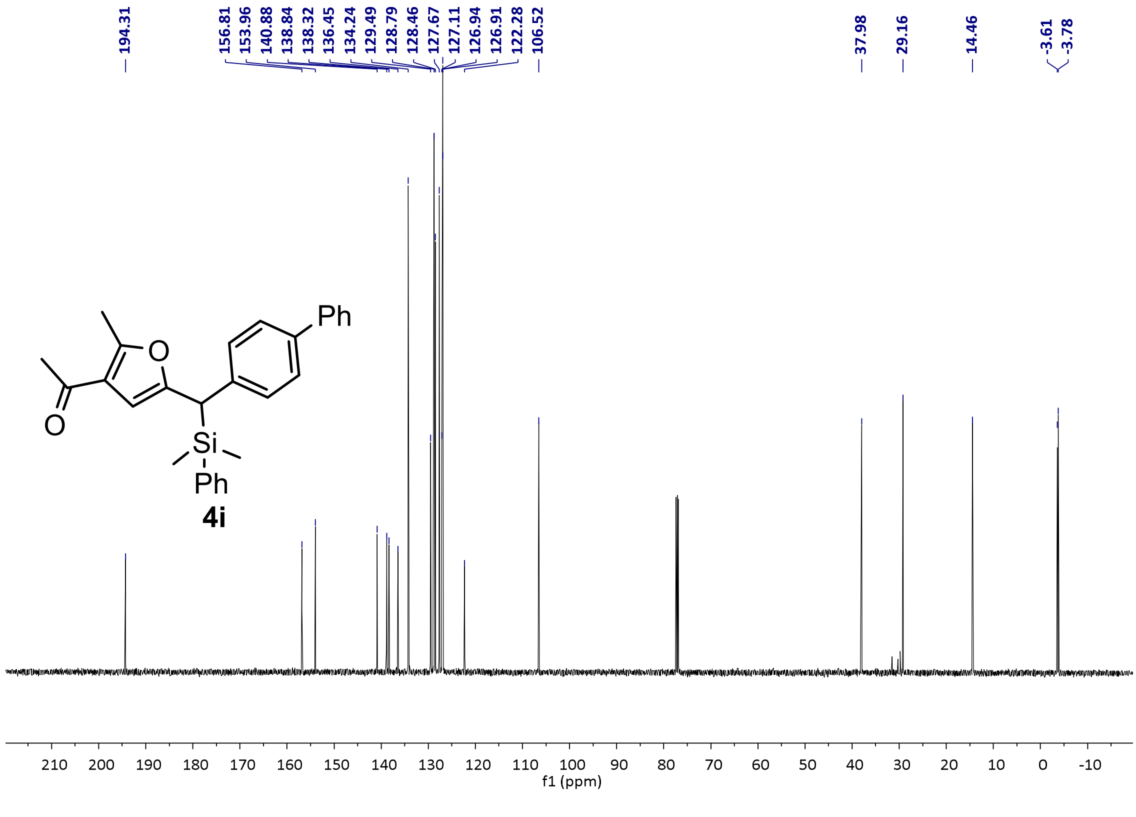

1-(5-((4-butylphenyl)(dimethyl(phenyl)silyl)methyl)-2-methylfuran-3-yl)ethan-1-one (4j)

^{1}H NMR

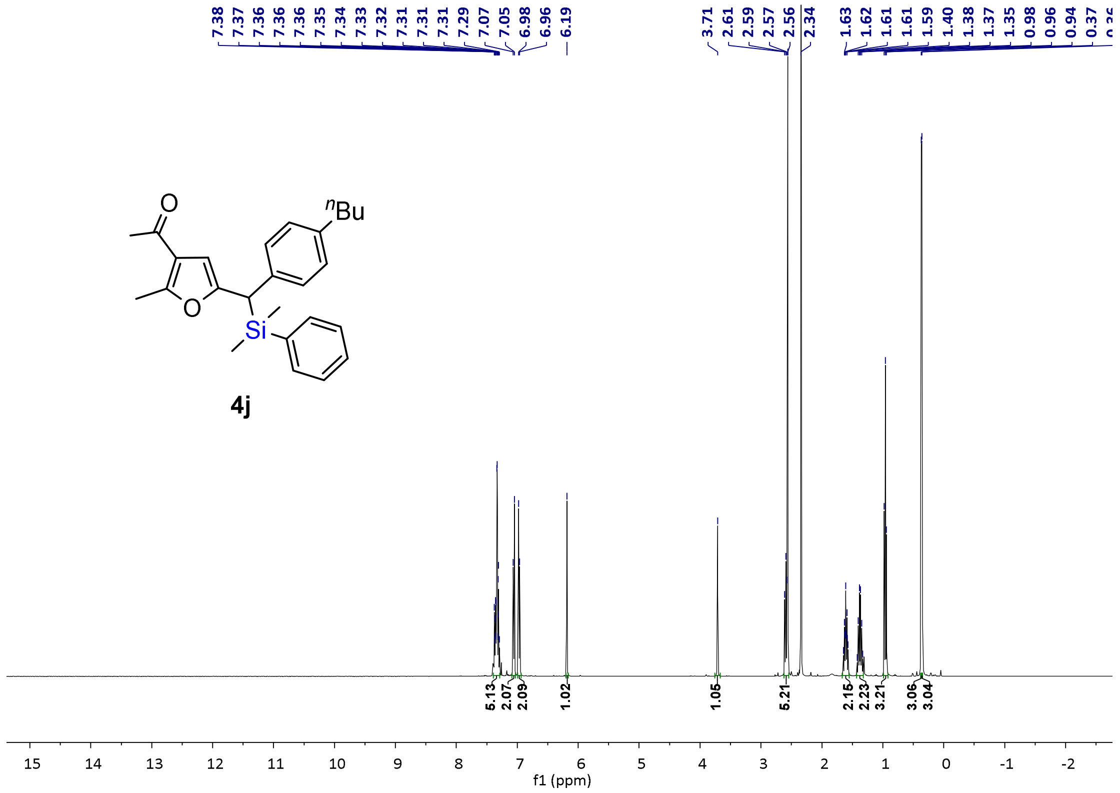

^{13}C NMR

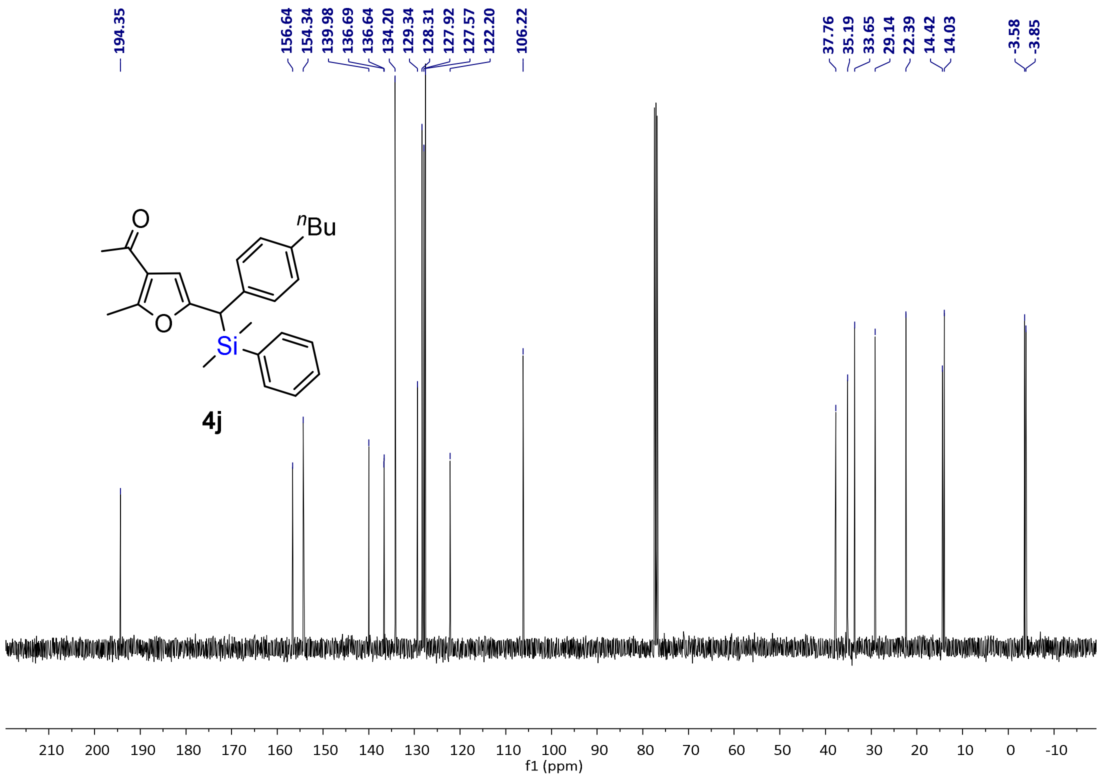

1-(5-((dimethyl(phenyl)silyl)(thiophen-3-yl)methyl)-2-methylfuran-3-yl)ethan-1-one (4k)

^{1}H NMR

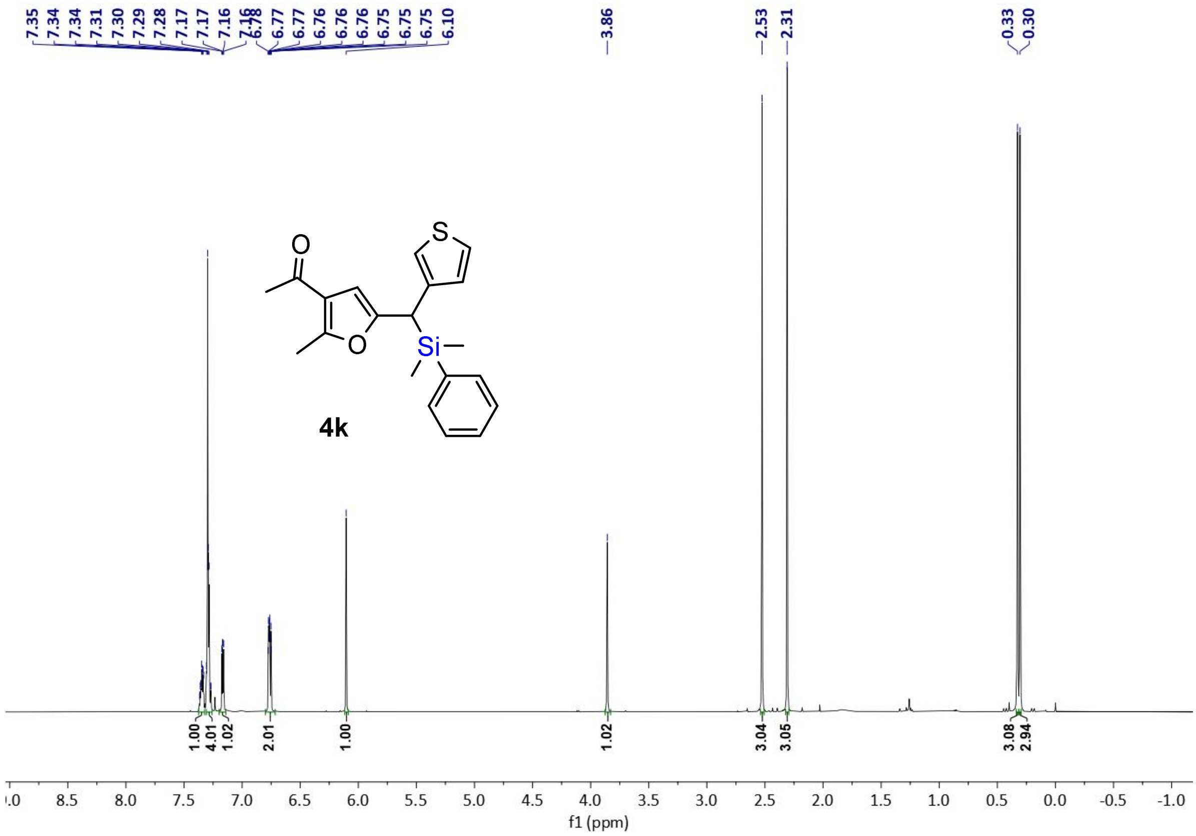
7.35 7.34 7.34 7.31 7.30 7.29 7.28 7.17 7.17 7.16 7.16 6.78 6.77 6.77 6.76 6.76 6.75 6.75 6.75 6.10
3.86
2.53 2.31
0.33 0.30
O
S
Si
O
4k
1.00 4.01 1.02 2.01 1.00 1.02 3.04 3.05 3.08 2.94
f1 (ppm)

^{13}C NMR

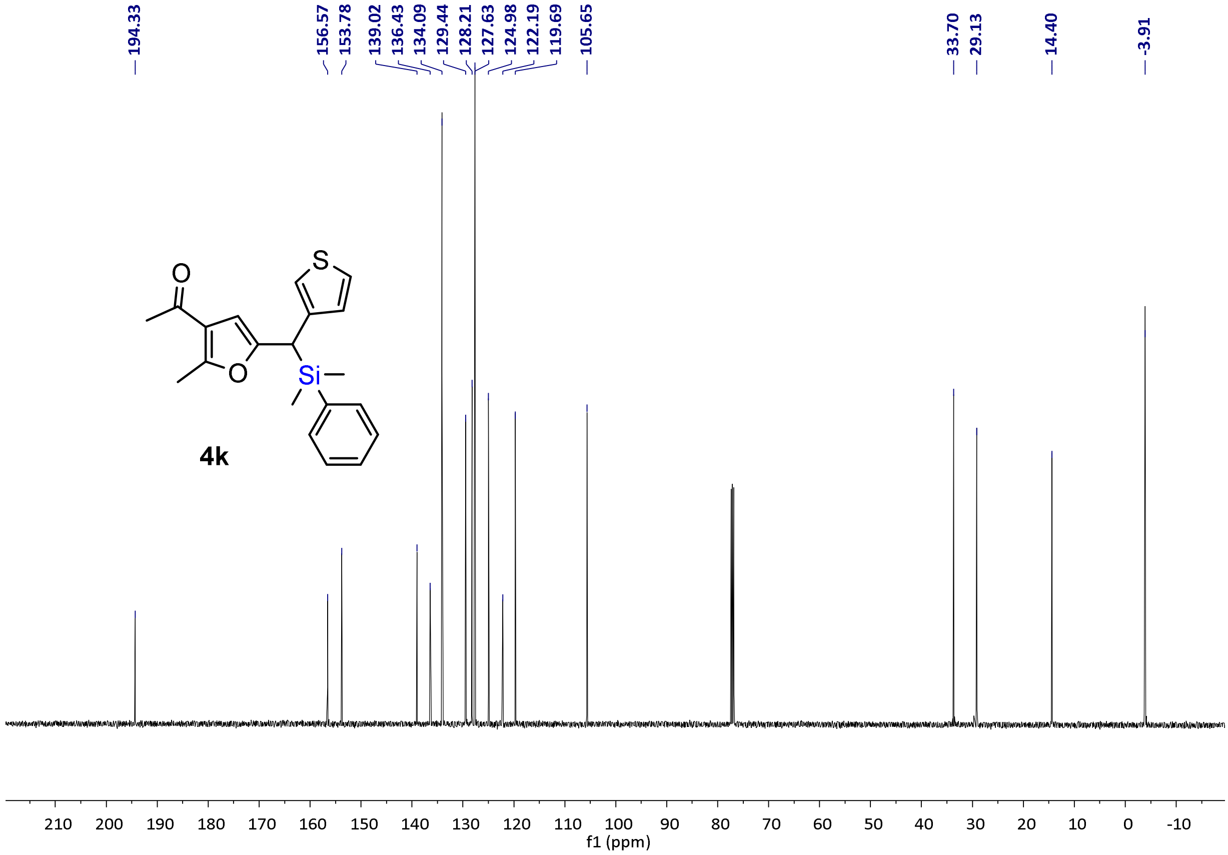
194.33
156.57 153.78 139.02 136.43 134.09 129.44 128.21 127.63 124.98 122.19 119.69 105.65
33.70 29.13 14.40 -3.91
O
S
Si
O
4k
f1 (ppm)

1-(5-(1-(dimethyl(phenyl)silyl)heptyl)-2-methylfuran-3-yl)ethan-1-one (4l)

^{1}H NMR

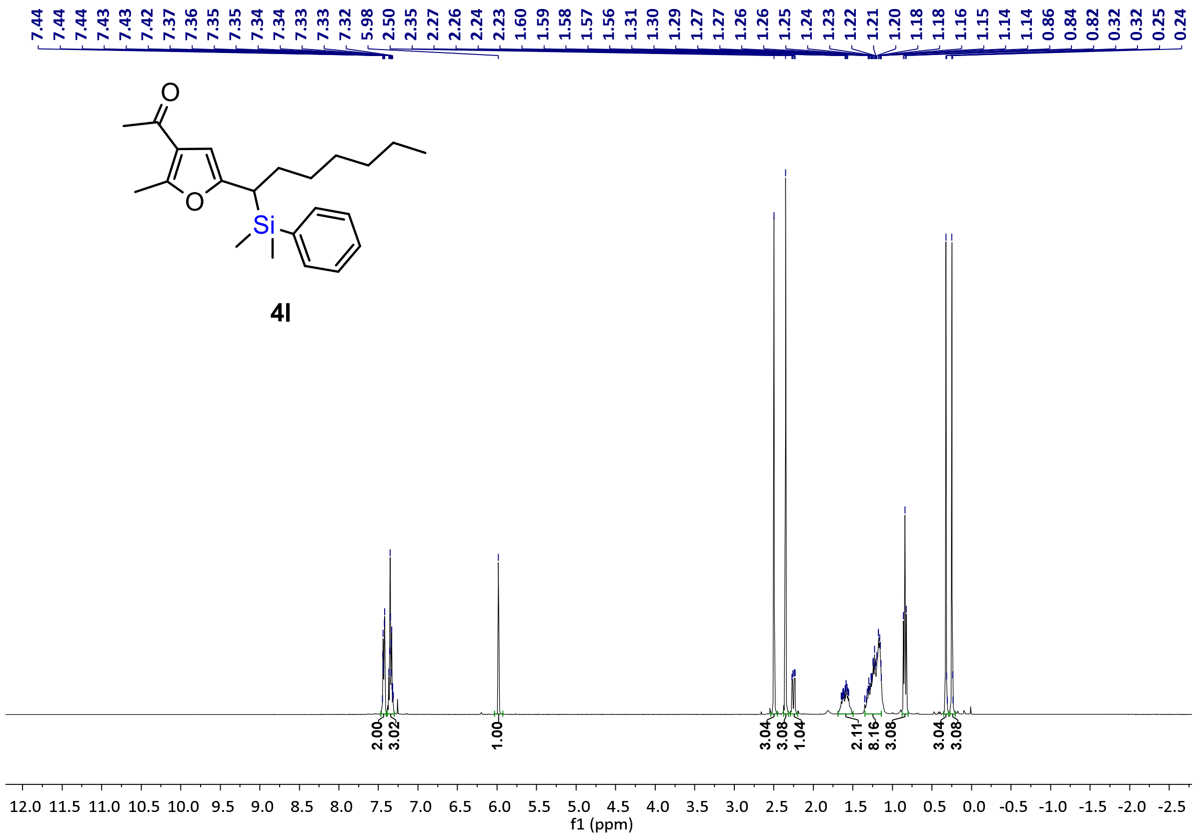

^{13}C NMR

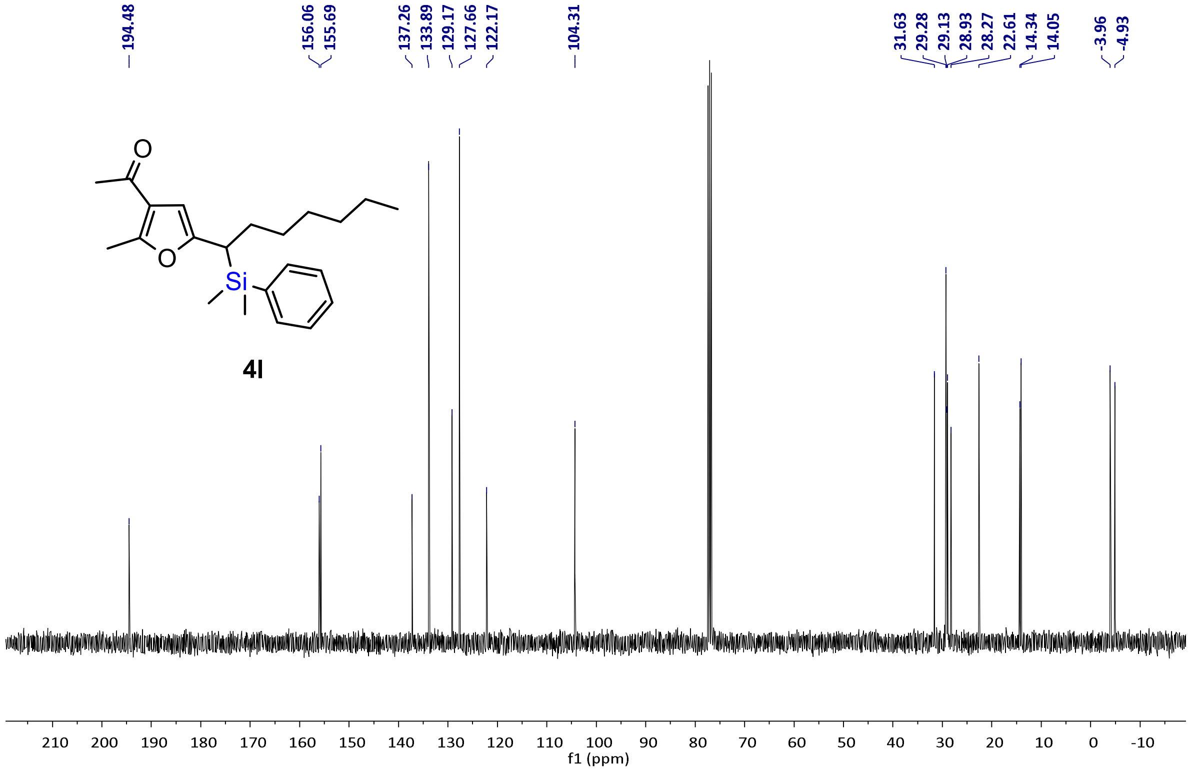

1-(5-((dimethyl(phenyl)silyl)(phenyl)methyl)-2-ethylfuran-3-yl)propan-1-one (4m)

^{1}H NMR

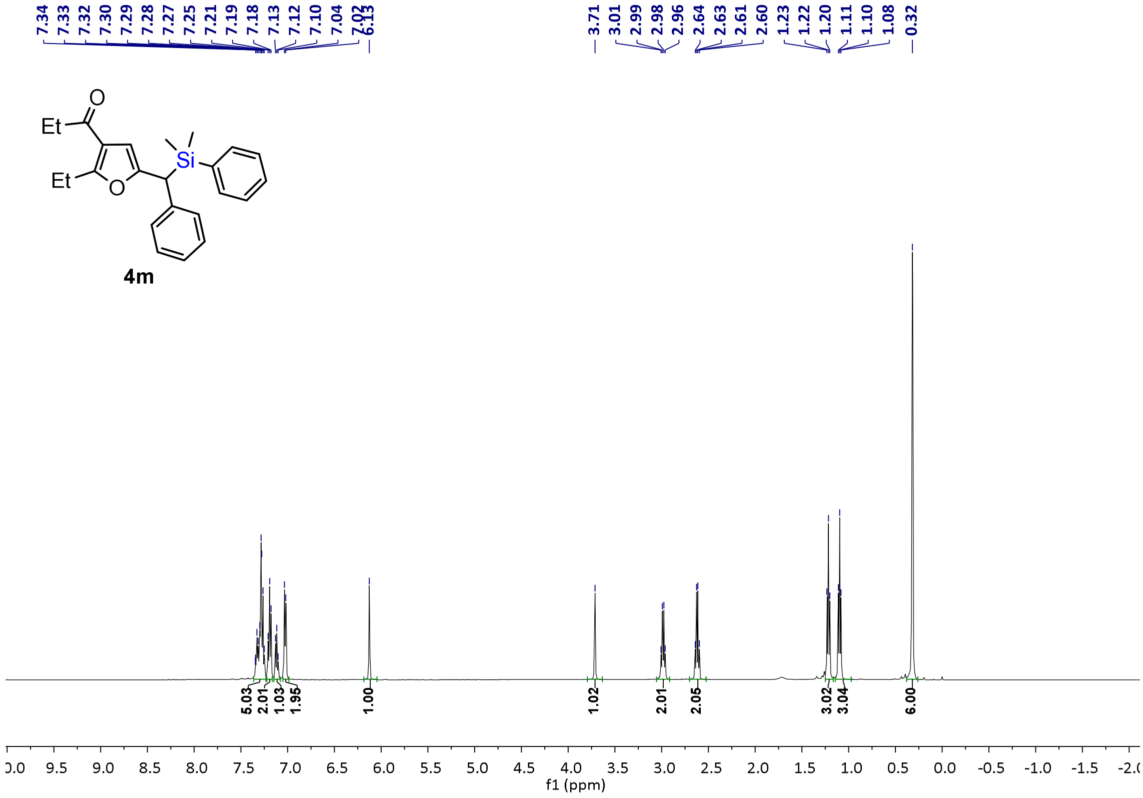

^{13}C NMR

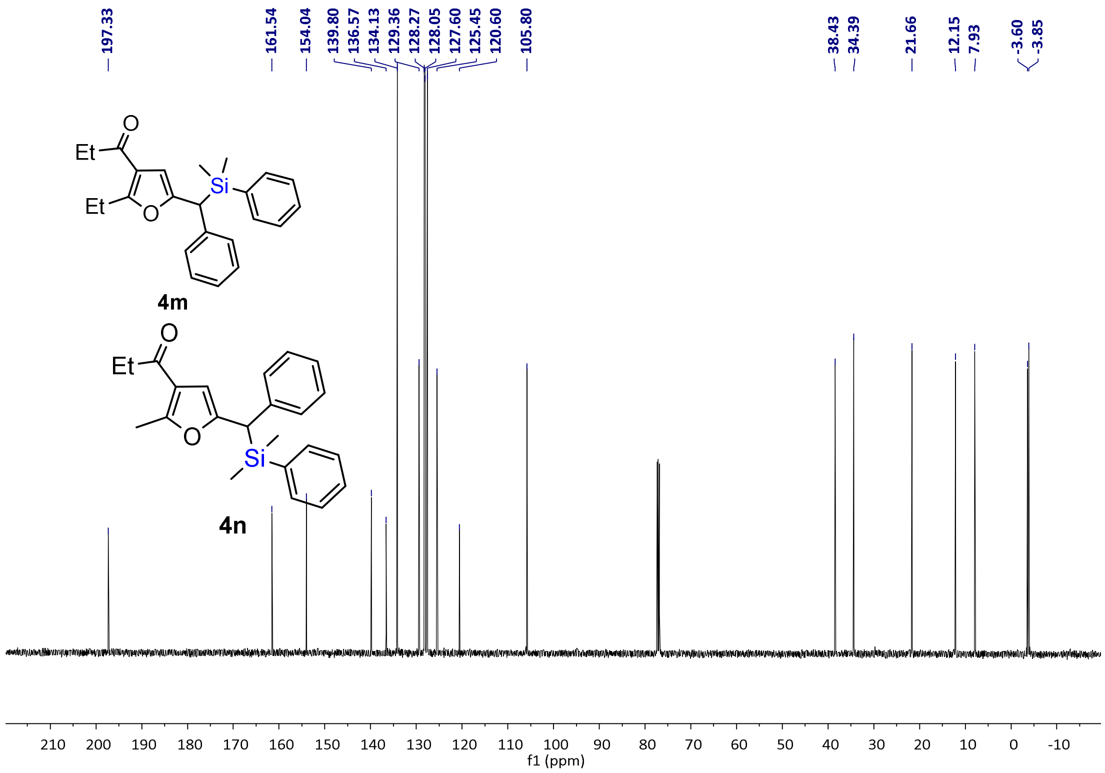

1-(5-((dimethyl(phenyl)silyl)(phenyl)methyl)-2-methylfuran-3-yl)propan-1-one (4n)

^{1}H NMR

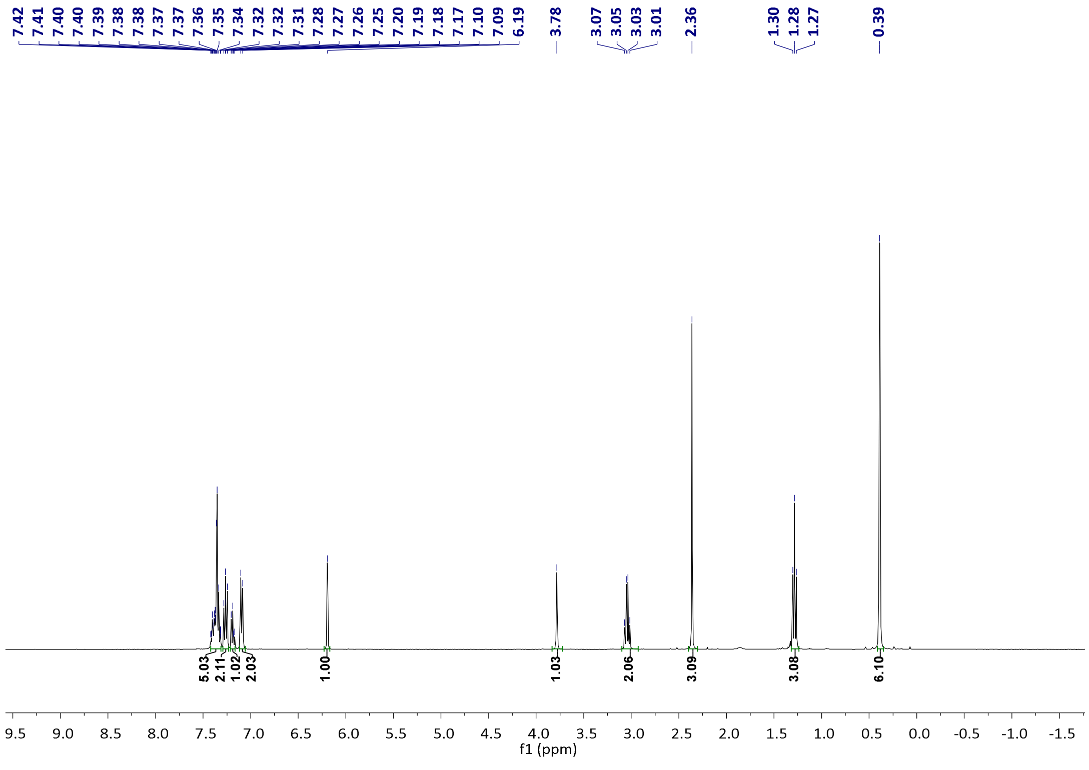

^{13}C NMR

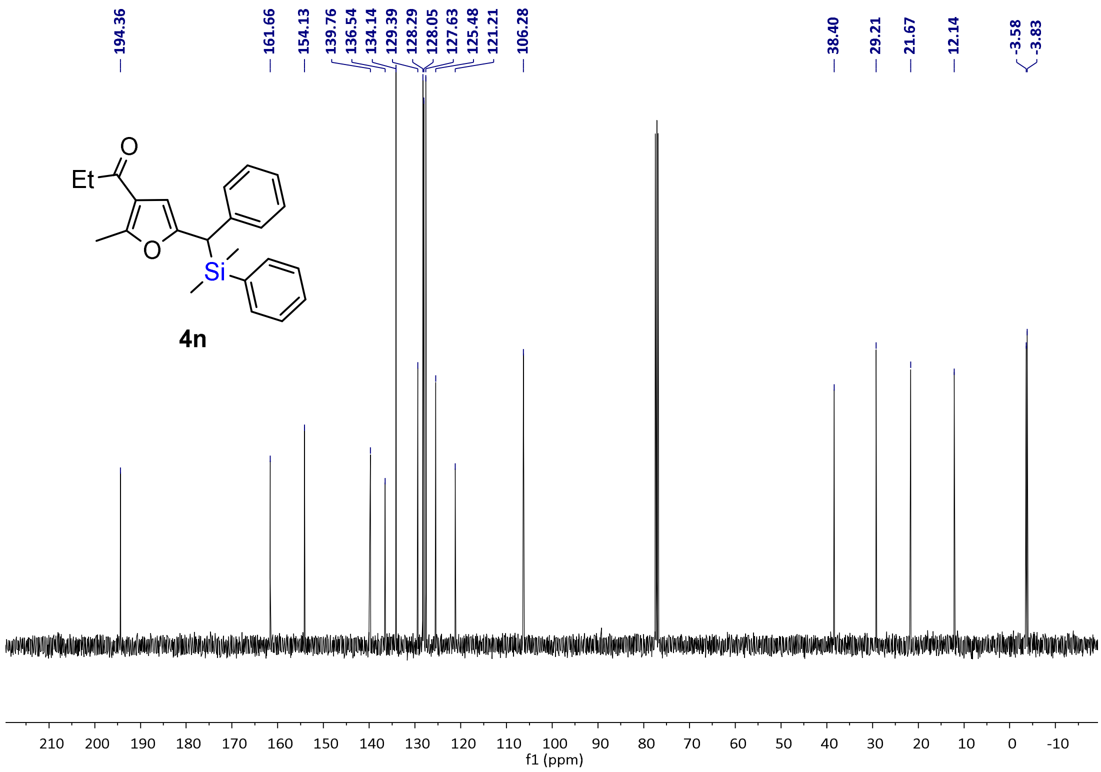

(5-((dimethyl(phenyl)silyl)(phenyl)methyl)-2-phenylfuran-3-yl)(phenyl)methanone (4o)

^{1}H NMR

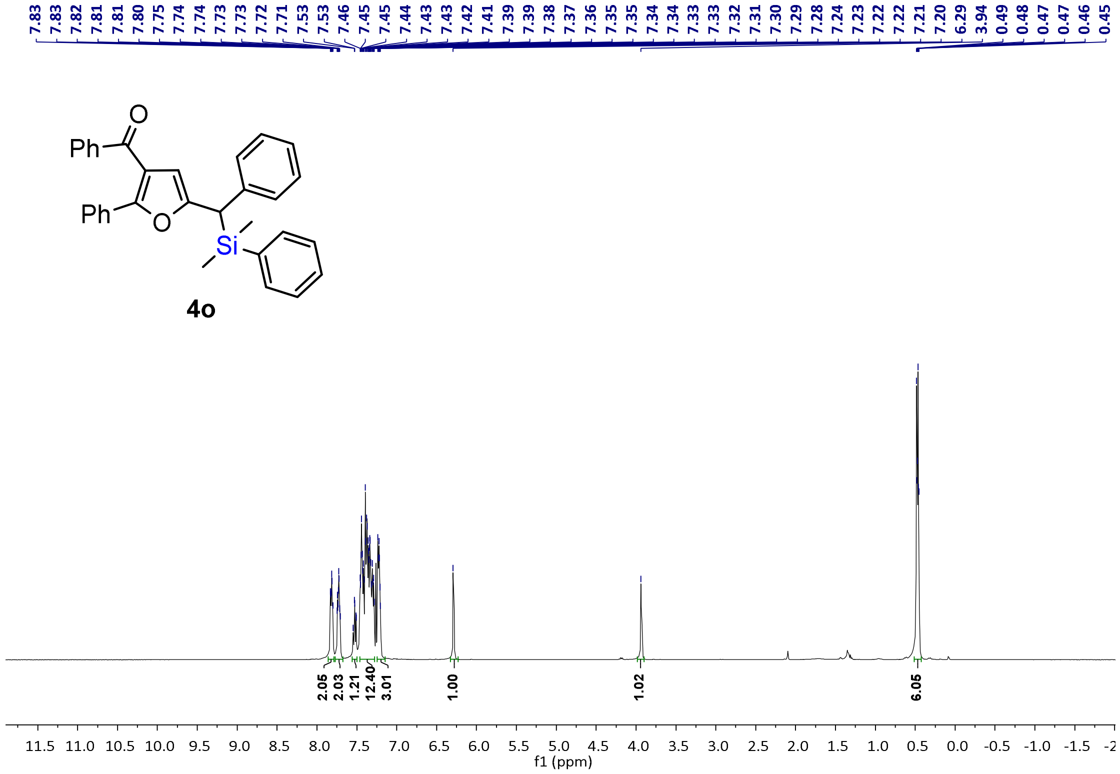

^{13}C NMR

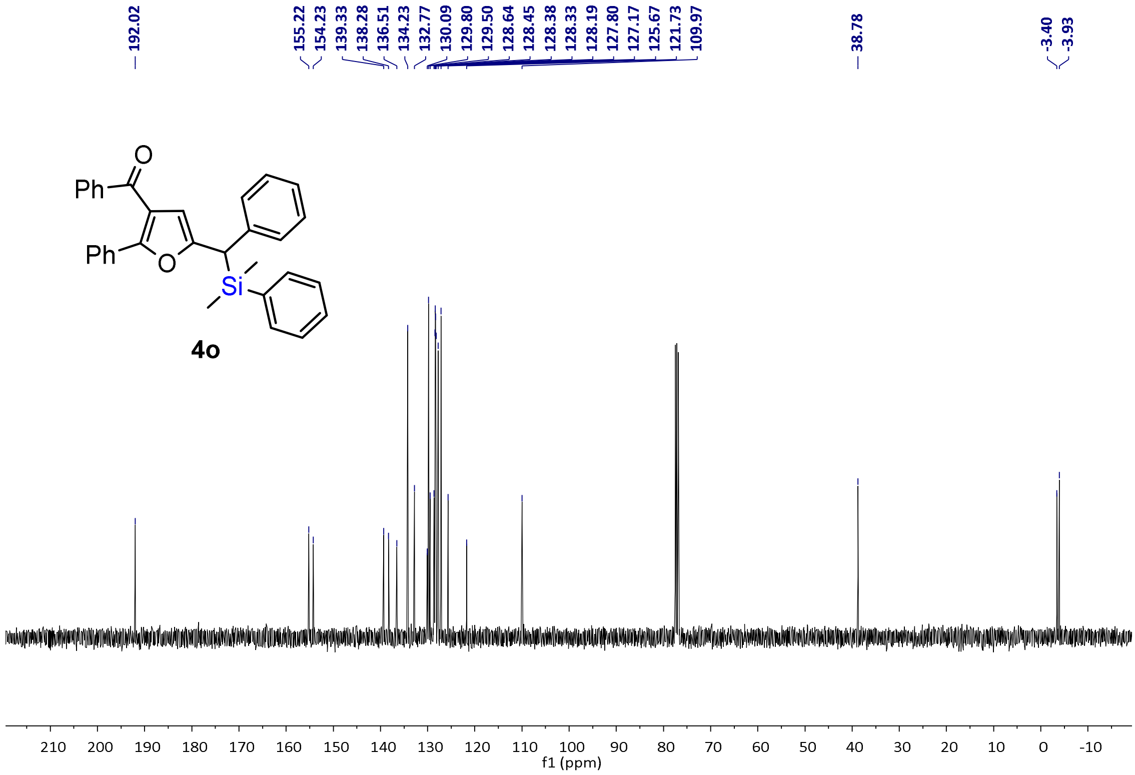

(5-(1-(dimethyl(phenyl)silyl)heptyl)-2-phenylfuran-3-yl)(phenyl)methanone (4p)

^{1}H NMR

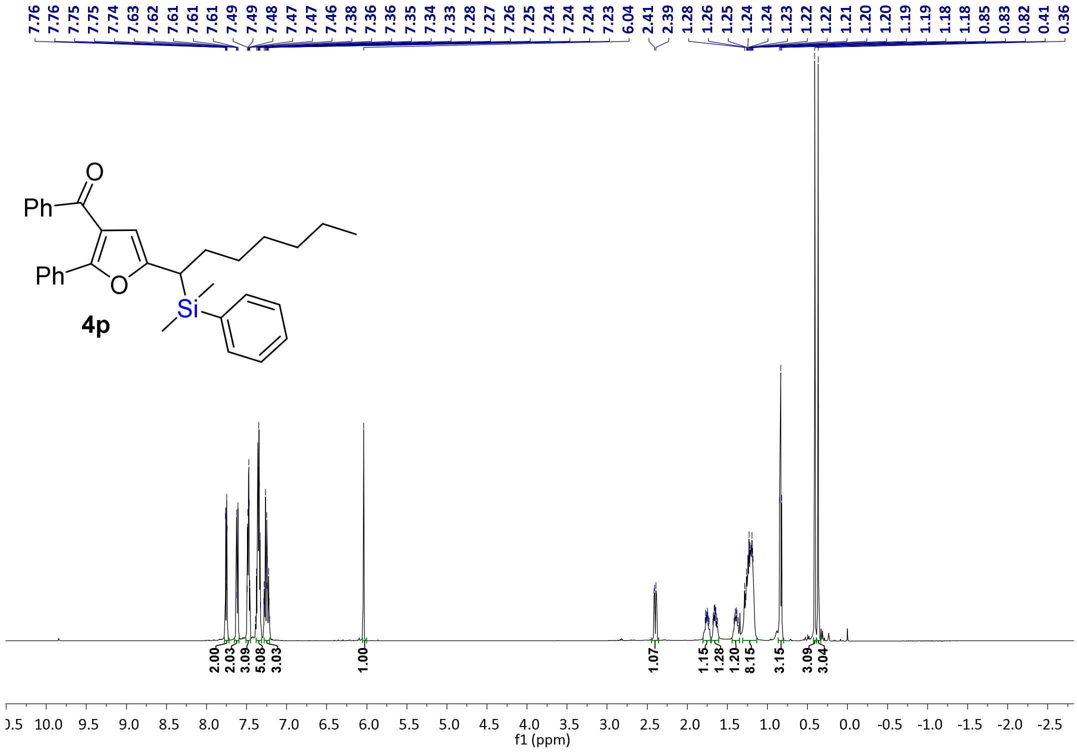

^{13}C NMR

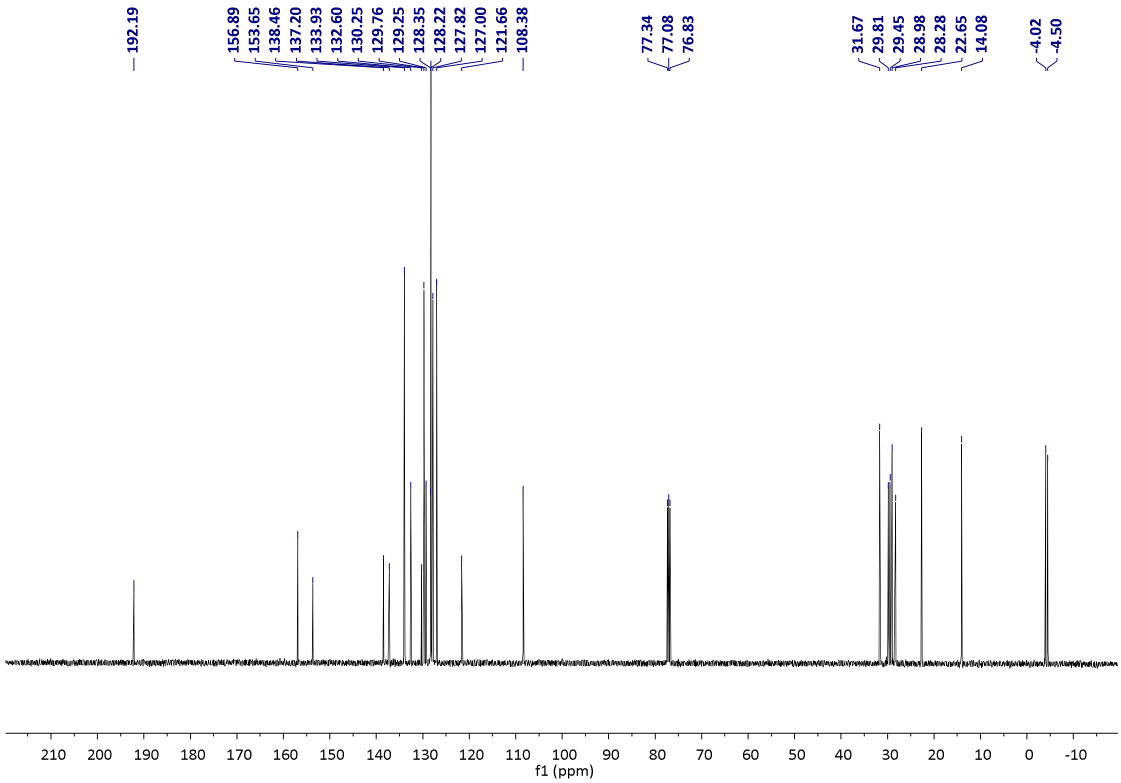

1-(5-(trimethylamine-boranyl(phenyl)methyl)-2-methylfuran-3-yl)ethan-1-one 6a)

^{1}H NMR

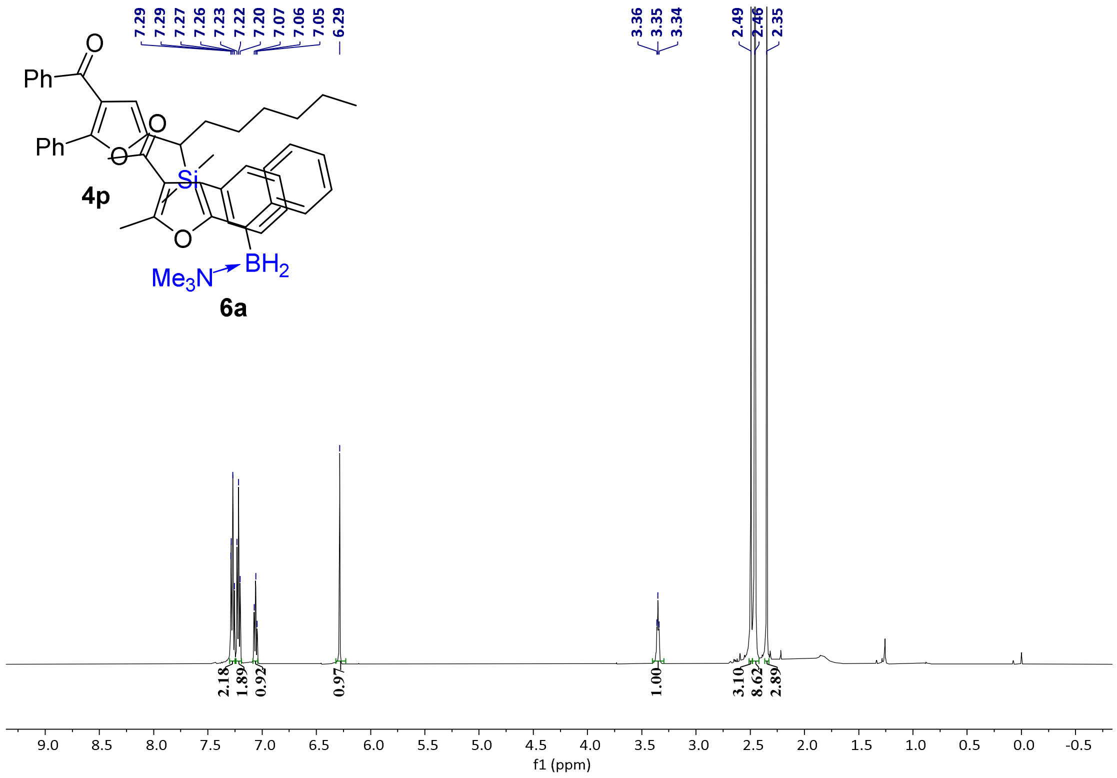

^{13}C NMR

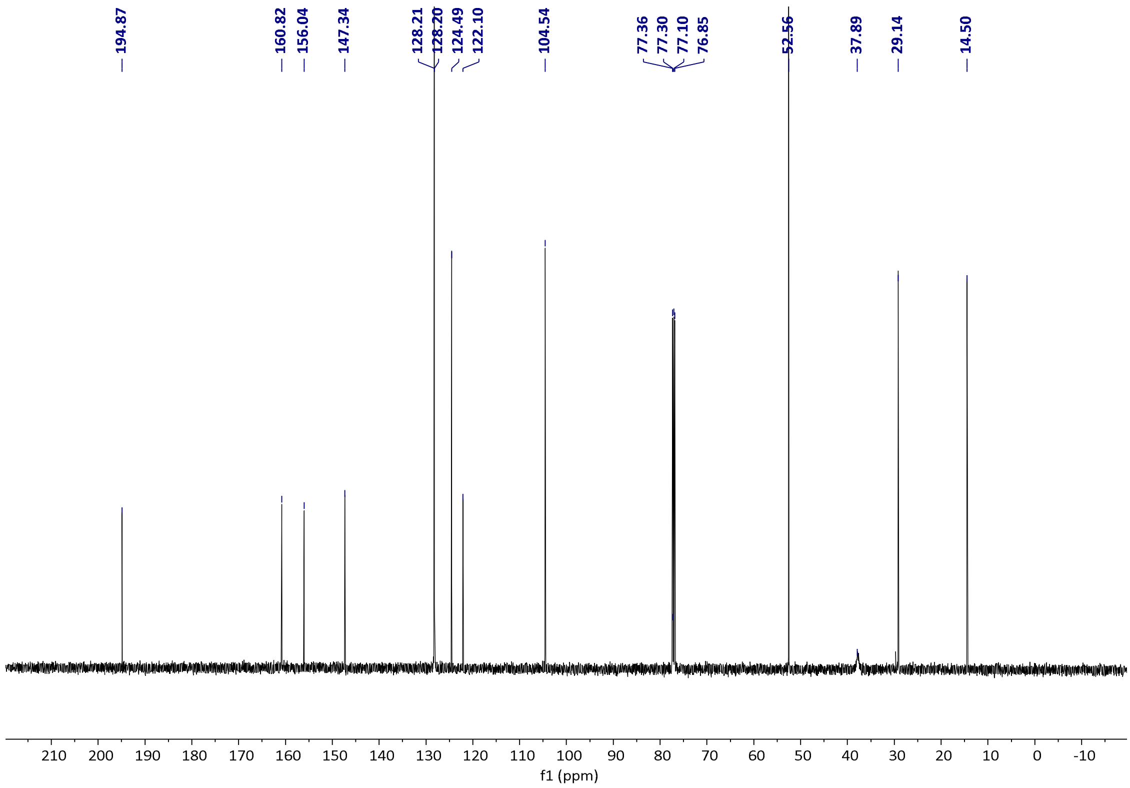

1-(5-(trimethylamine-boranyl(p-tolyl)methyl)-2-methylfuran-3-yl)ethan-1-one (6b)

^{1}H NMR

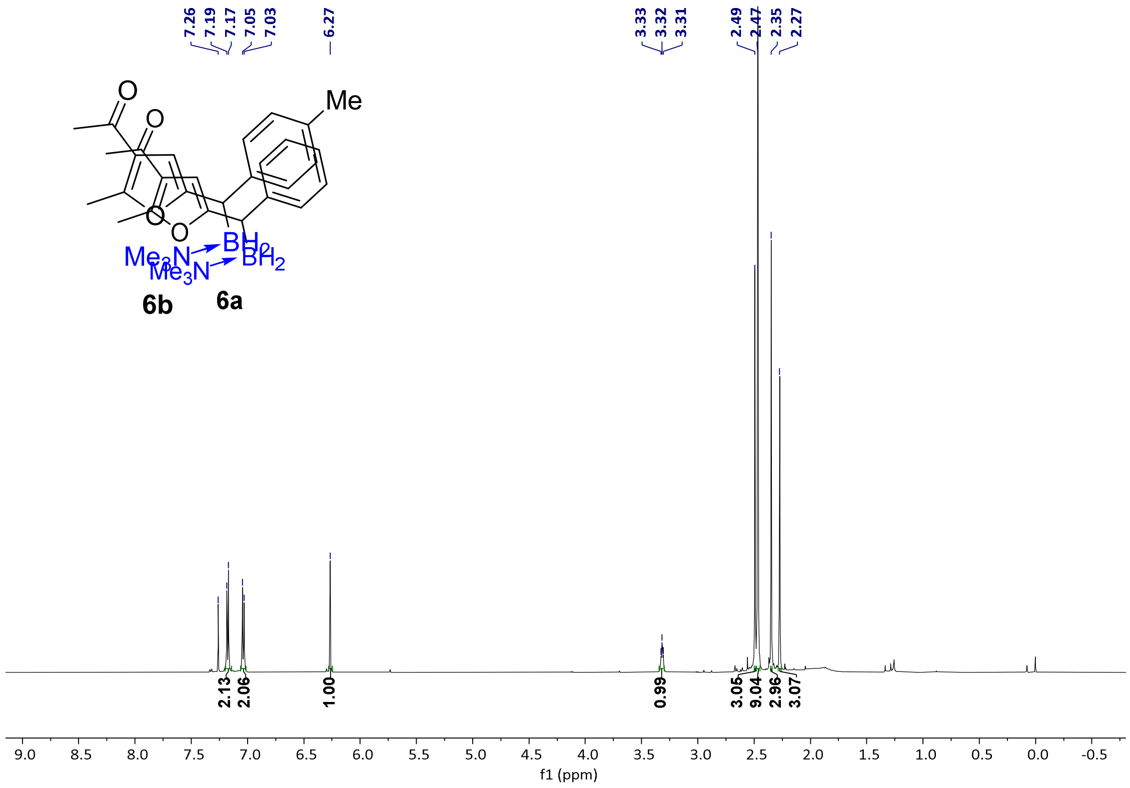

^{13}C NMR

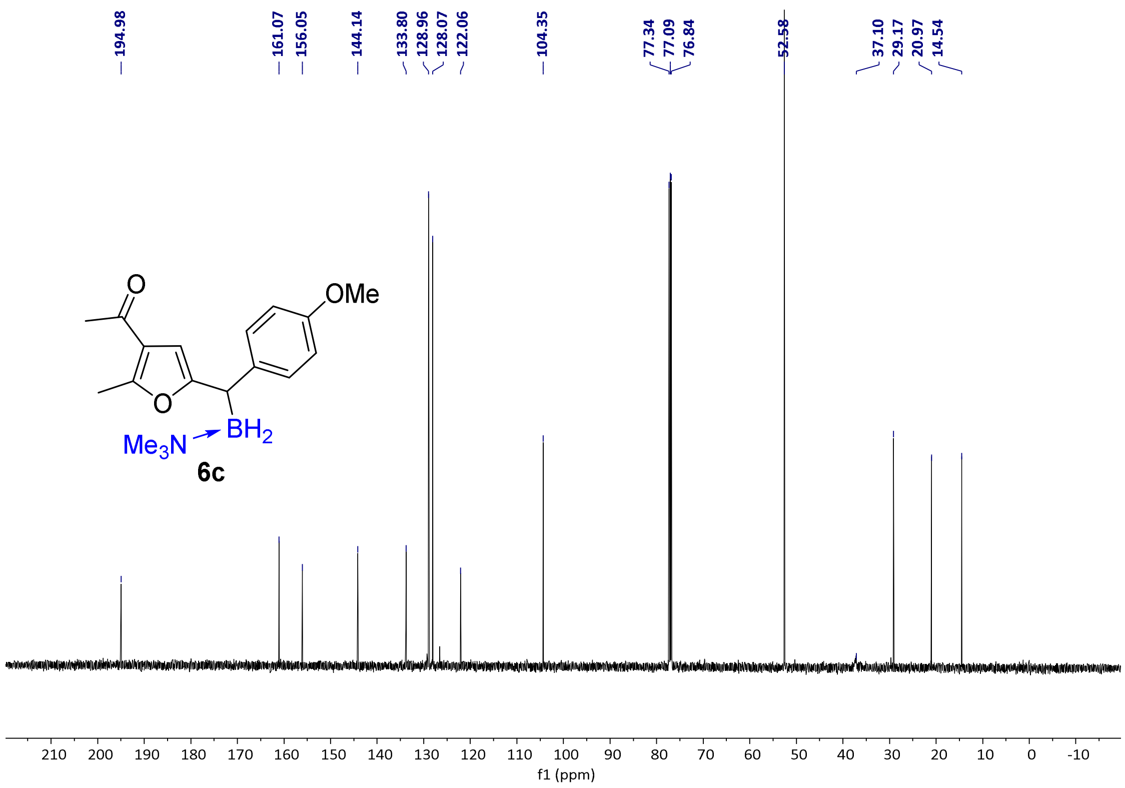

1-(5-(trimethylamine-boranyl(4-methoxyphenyl)methyl)-2-methylfuran-3-yl)ethan-1-one (6c)

^{1}H NMR

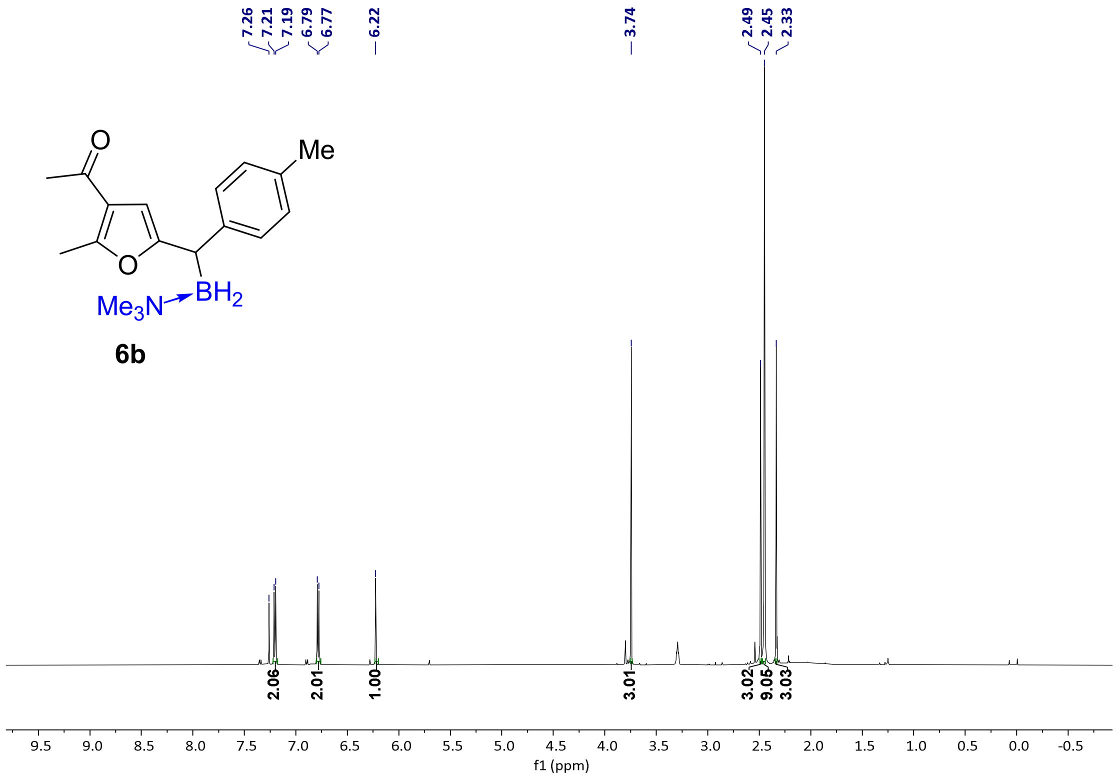

^{13}C NMR

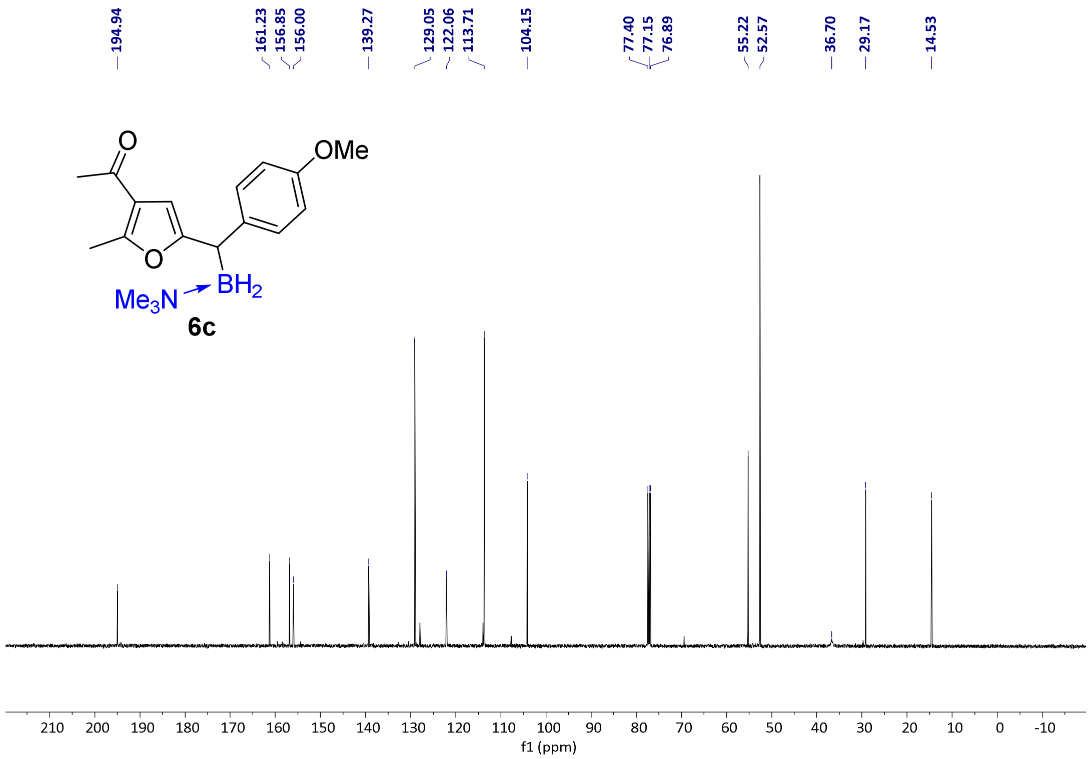

1-(5-(trimethylamine-boranyl(4-chlorophenyl)methyl)-2-methylfuran-3-yl)ethan-1-one (6d)

^{1}H NMR

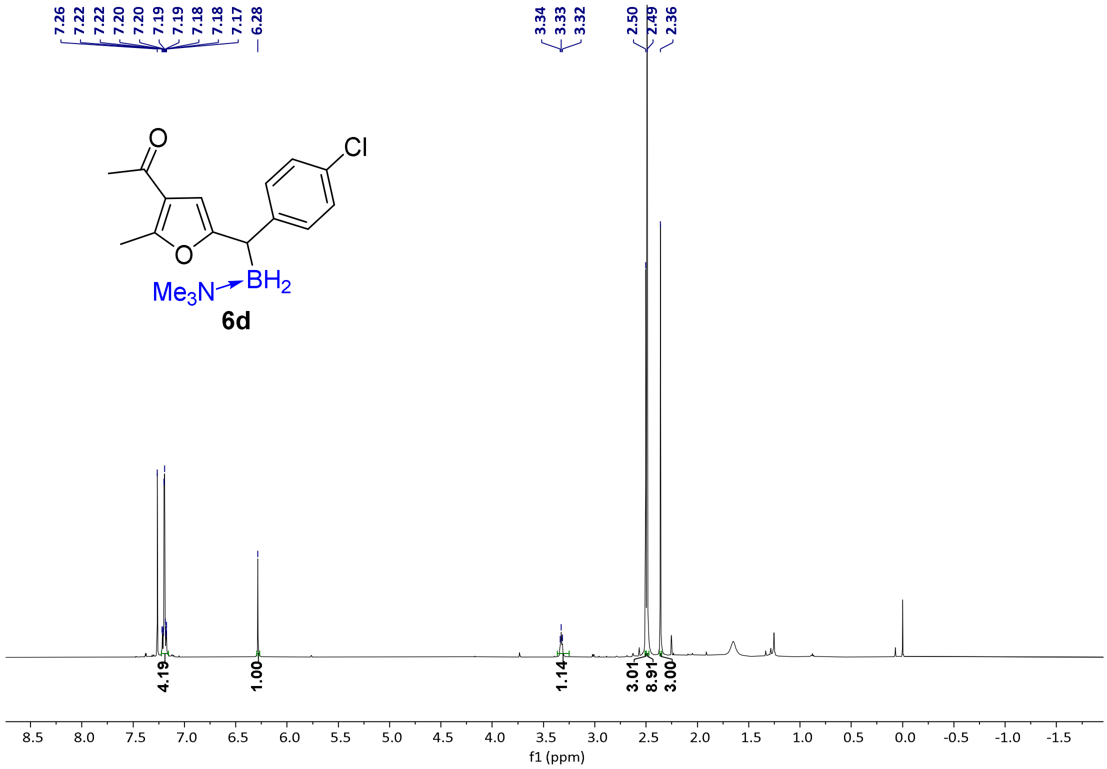

^{13}C NMR

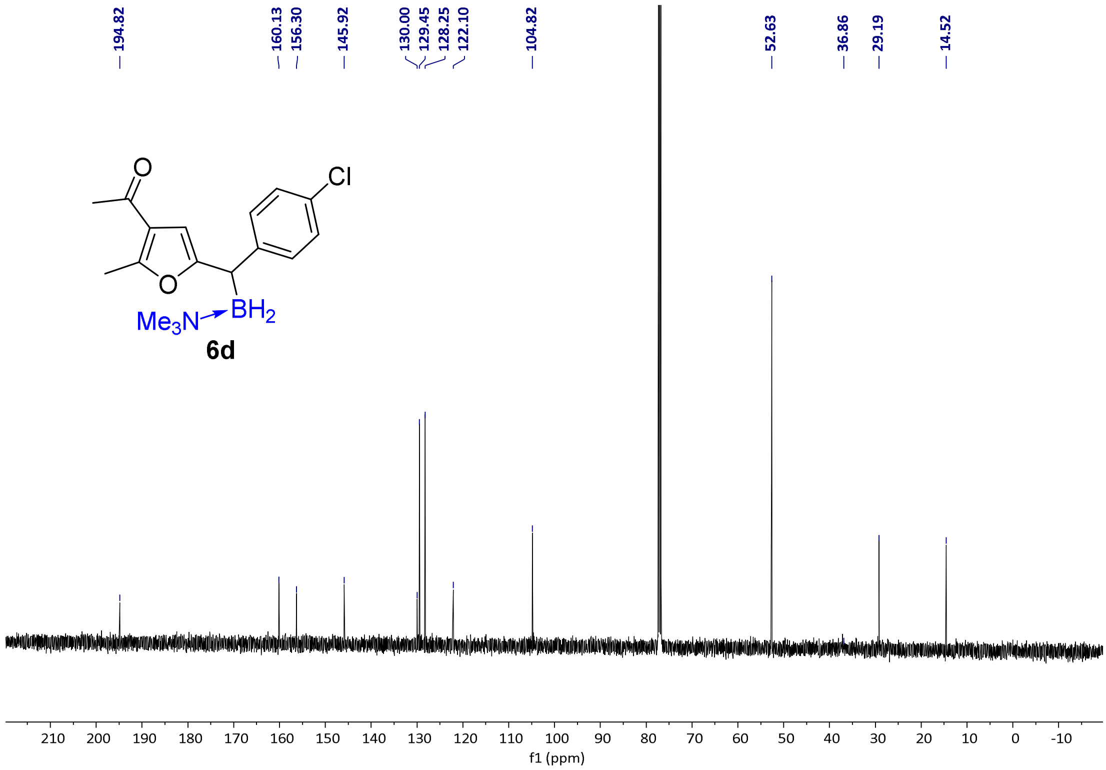

1-(5-(trimethylamine-boranyl(*m*-tolyl))methyl)-2-methylfuran-3-yl)ethan-1-one (6e)

^{1}H NMR

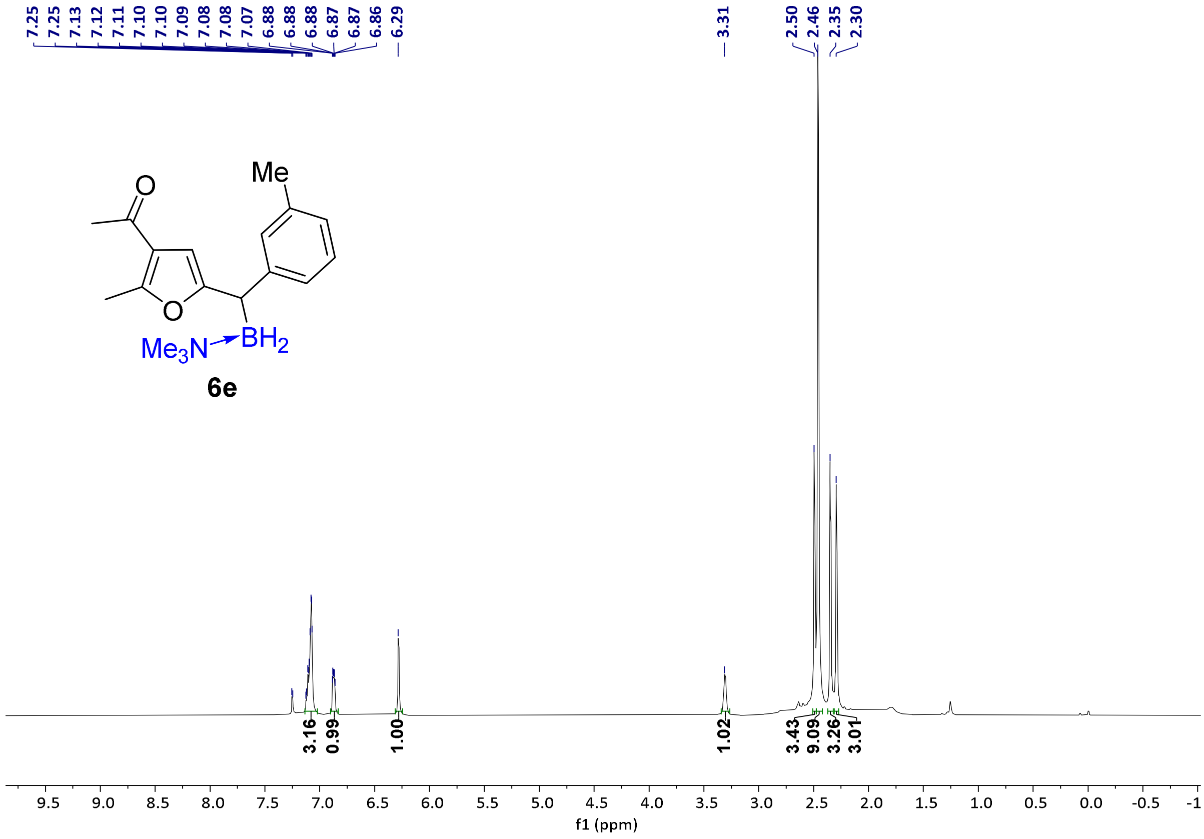

^{13}C NMR

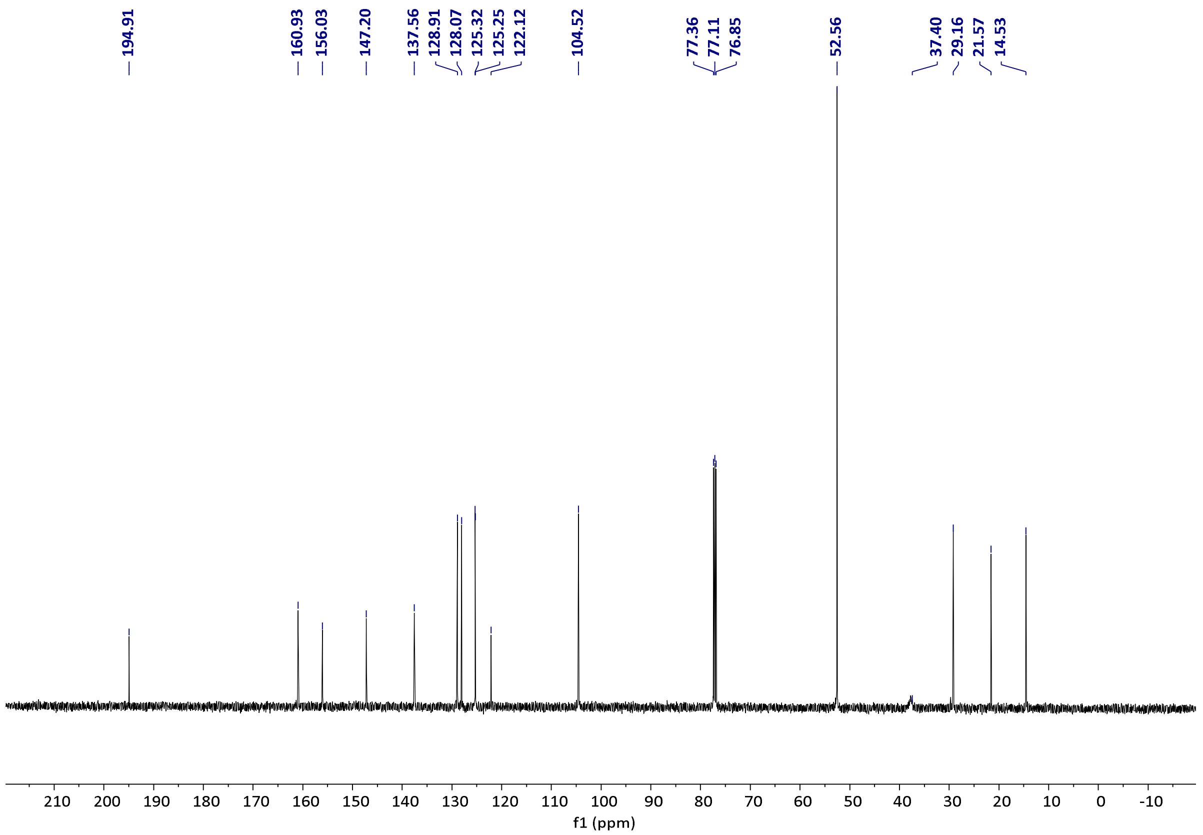

1-(5-(trimethylamine-boranyl(3-methoxyphenyl)methyl)-2-methylfuran-3-yl)ethan-1-one (6f)

^{1}H NMR

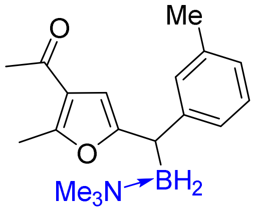

6e

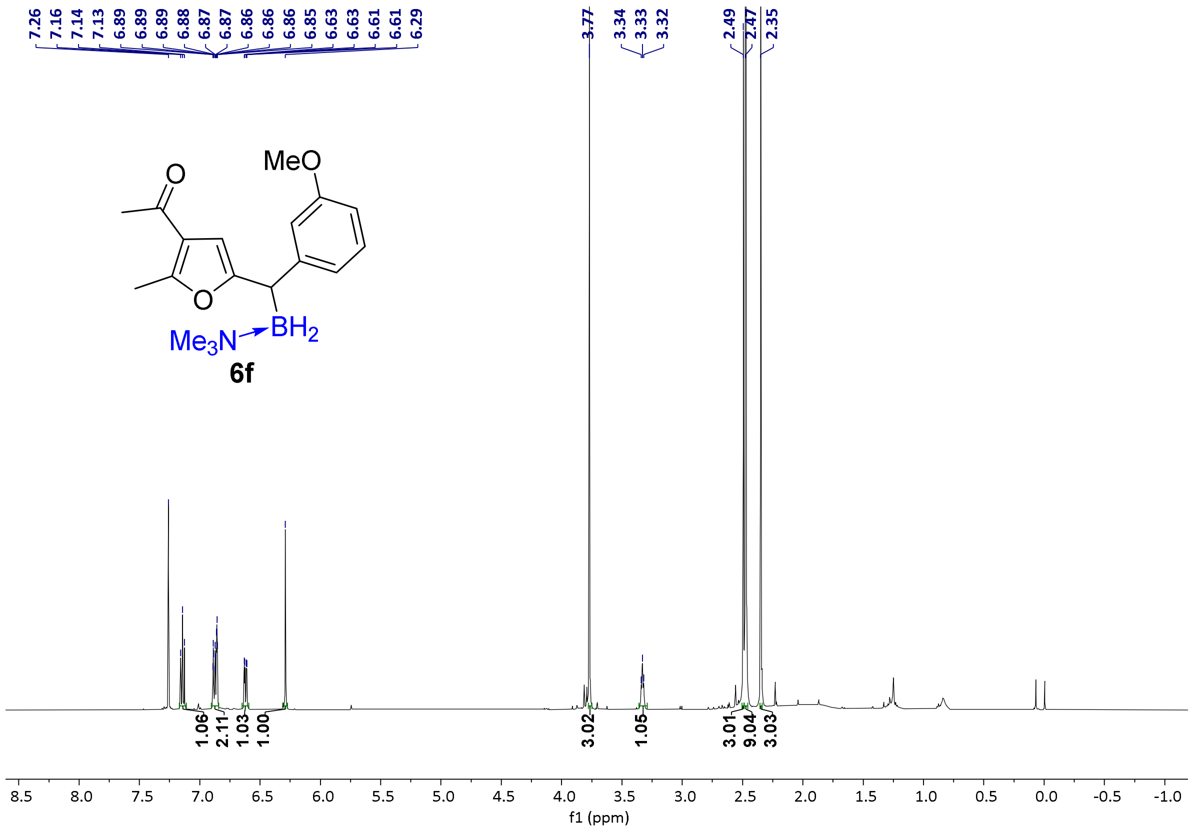

7.26 7.16 7.14 7.13 6.89 6.89 6.89 6.88 6.87 6.87 6.86 6.86 6.86 6.85 6.63 6.63 6.61 6.61 6.29
3.77 3.34 3.33 3.32
2.49 2.47 2.35
MeO
O
Me3N
BH2
6f
1.06 2.11 1.03 1.00
3.02 1.05
3.01 9.04 3.03
8.5 8.0 7.5 7.0 6.5 6.0 5.5 5.0 4.5 4.0 3.5 3.0 2.5 2.0 1.5 1.0 0.5 0.0 -0.5 -1.0
f1 (ppm)

^{13}C NMR

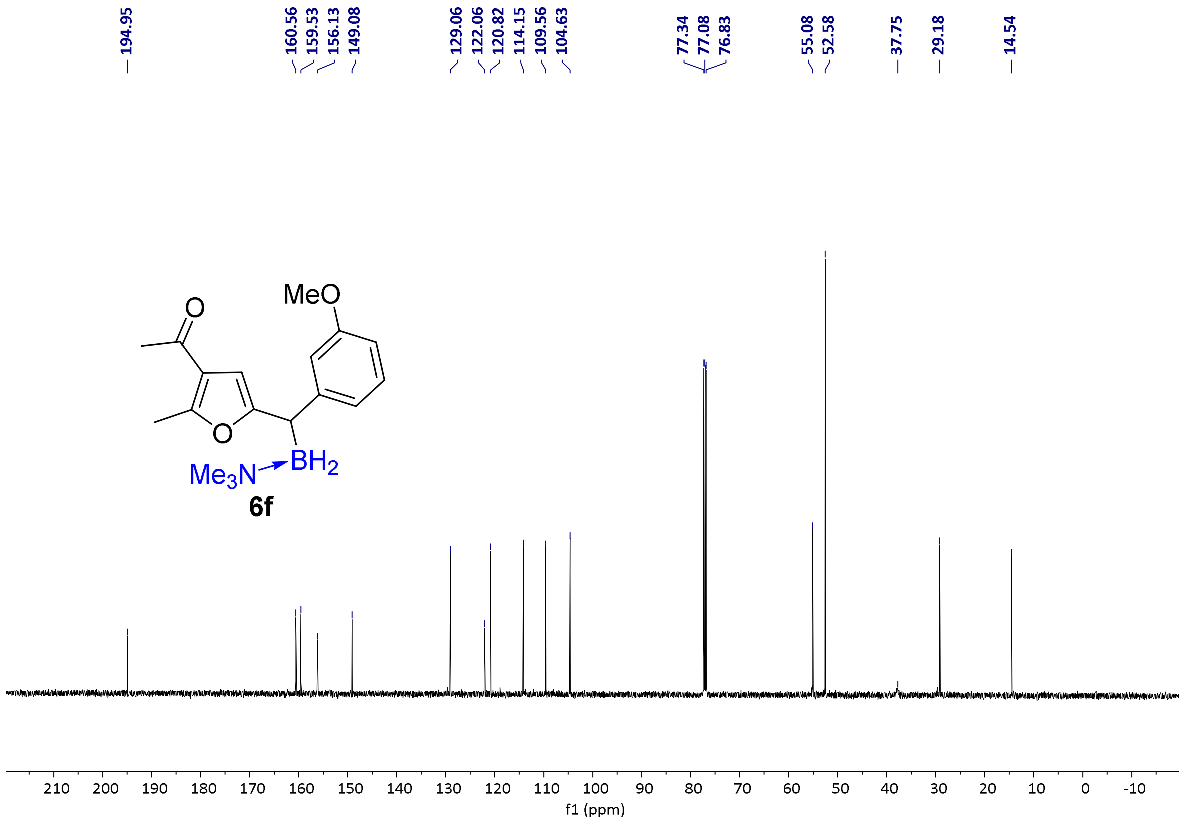

194.95
160.56 159.53 156.13 149.08
129.06 122.06 120.82 114.15 109.56 104.63
77.34 77.08 76.83
55.08 52.58
37.75 29.18 14.54
MeO
O
Me3N
BH2
6f
210 200 190 180 170 160 150 140 130 120 110 100 90 80 70 60 50 40 30 20 10 0 -10
f1 (ppm)

1-(5-(trimethylamine-boranyl(phenyl)methyl)-2-ethylfuran-3-yl)propan-1-one (6h)

^{1}H NMR

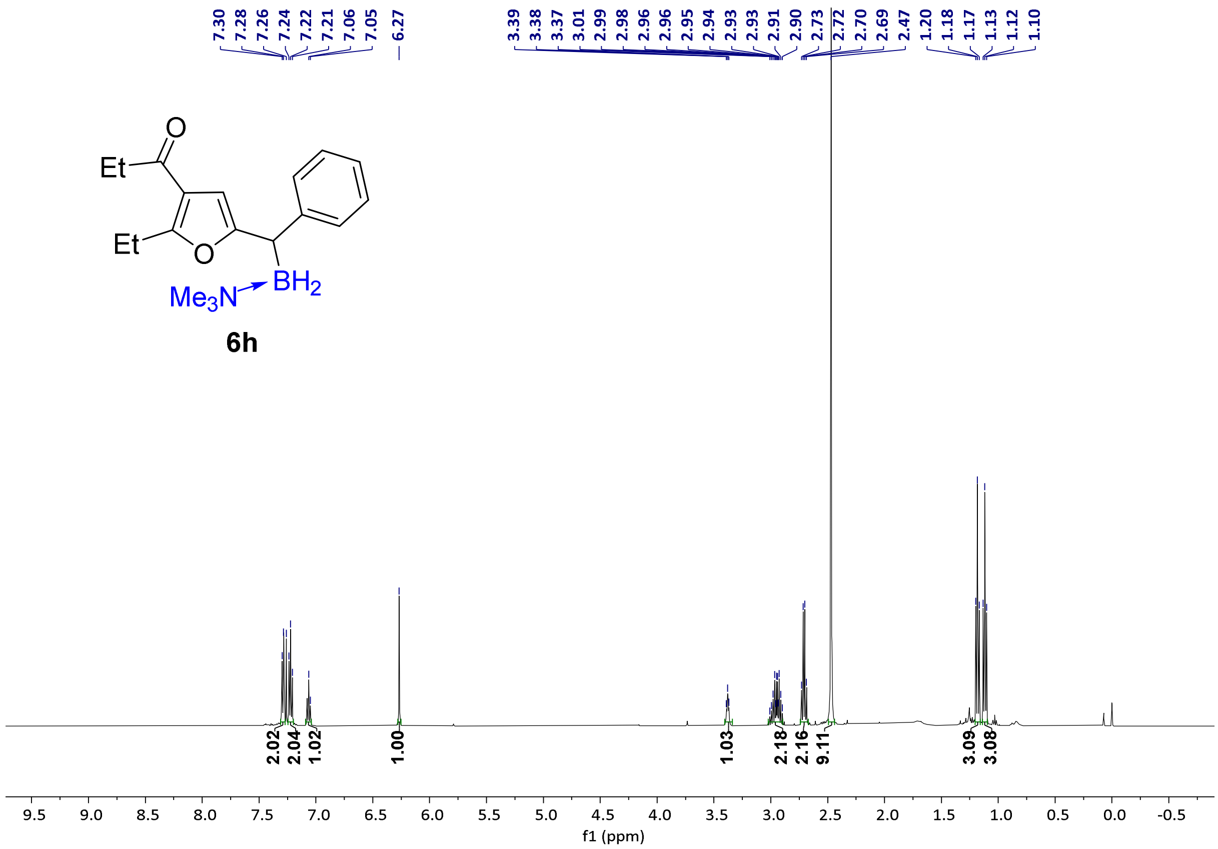

^{13}C NMR

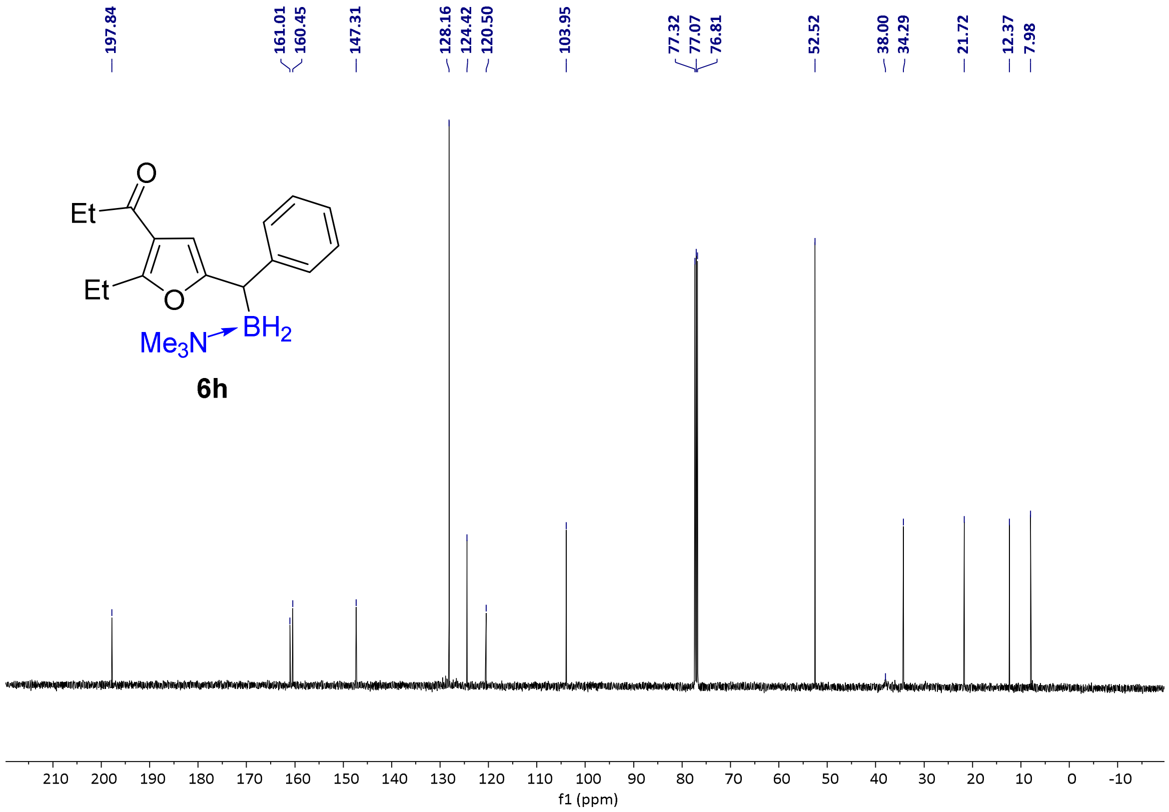

l(5-(trimethylamine-boranyl(phenyl)methyl)-2-phenylfuran-3-yl)(phenyl)methanone (6i)

^{1}H NMR

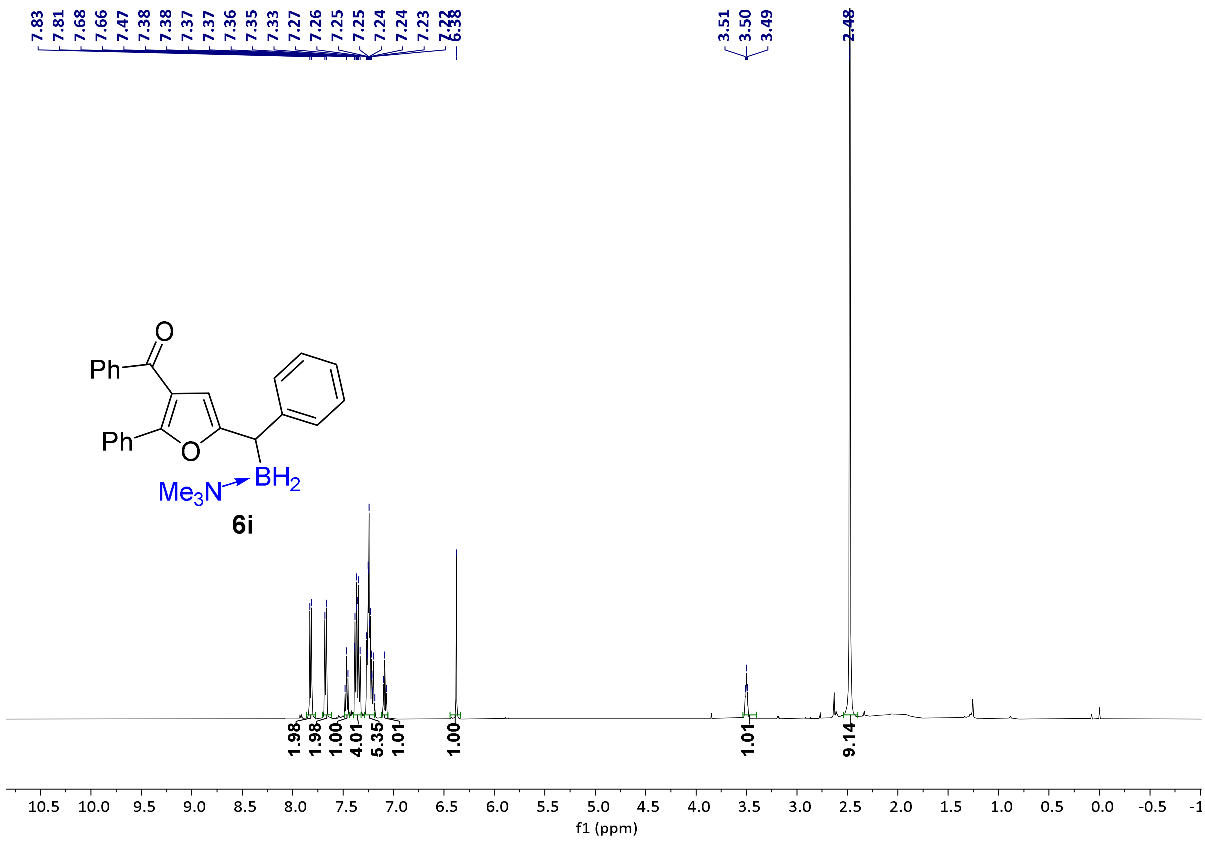
7.83
7.81
7.68
7.66
7.47
7.38
7.38
7.37
7.37
7.36
7.35
7.33
7.27
7.26
7.25
7.25
7.24
7.24
7.23
7.22
6.38
3.51
3.50
3.49
2.48
Ph
O
Ph
O
Me3N
BH2
6i
1.98
1.98
1.00
4.01
5.35
1.01
1.00
1.01
9.14
f1 (ppm)

^{13}C NMR

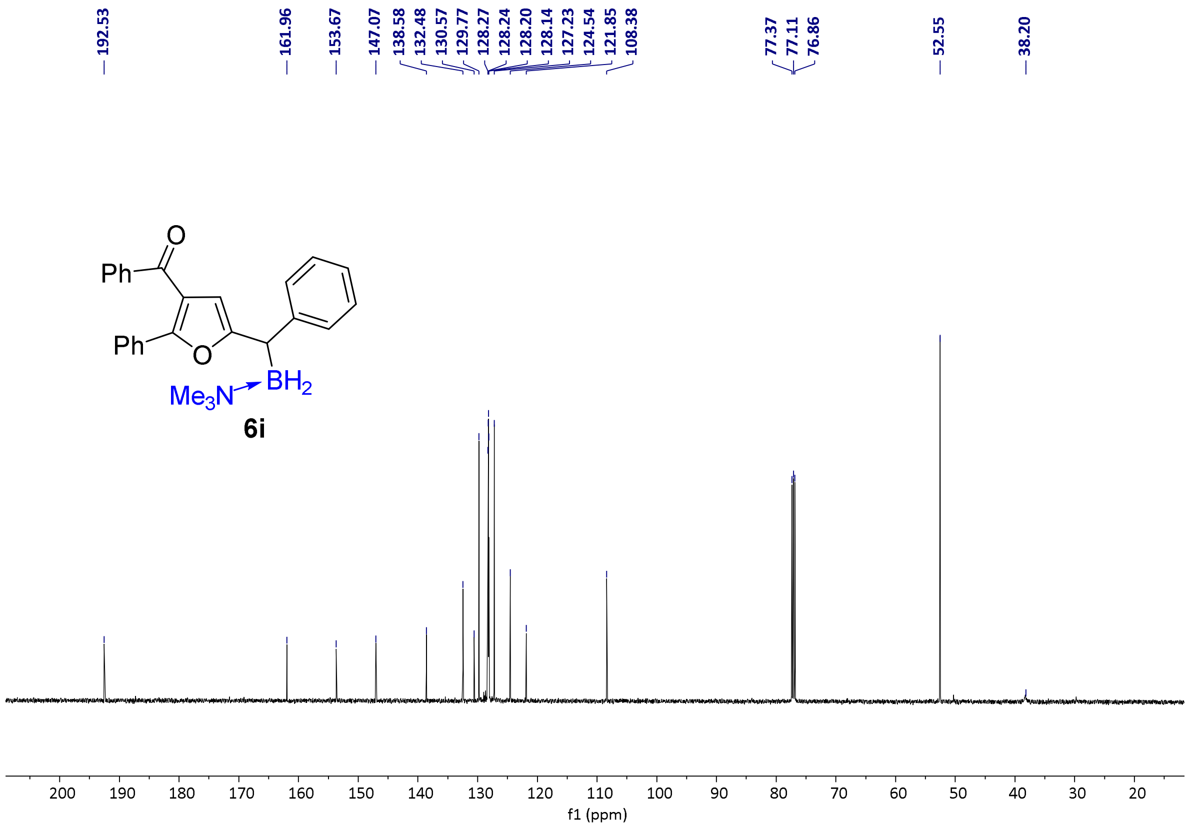
192.53
161.96
153.67
147.07
138.58
132.48
130.57
129.77
128.27
128.24
128.20
128.14
127.23
124.54
121.85
108.38
77.37
77.11
76.86
52.55
38.20
Ph
O
Ph
O
Me3N
BH2
6i
f1 (ppm)
